# Supplementary material for: Combined association of obesity and other cardiometabolic diseases with severe COVID-19 outcomes: a nationwide cross-sectional study of 21 773 Brazilian adult and elderly inpatients
Source: BMJ Open. 2021 Aug 9;11(8):e050739. doi: 10.1136/bmjopen-2021-050739 (PMC8354760; doi:10.1136/bmjopen-2021-050739)
Supplement: Supplementary data [file bmjopen-2021-050739supp001.pdf]

Suppl. Table 1. Characteristics of the study population and study samples included and excluded of the analysis for each severe COVID-19 outcome.

	Study population*		Mechanical ventilation				ICU admission				Death			
			Included		Excluded		Included		Excluded		Included		Excluded	
	n	%	n	%	n	%	n	%	n	%	n	%	n	%
Overall	21,773	100.0	19,904	91.4	1,869	8.6	20,636	94.8	1,137	5.2	16,508	75.8	5,265	24.2
**Sex**														
Female	9,742	44.7	8,905	44.7	837	44.8	9,227	44.7	515	45.3	7,379	44.7	2,363	44.9
Male	12,031	55.3	10,999	55.3	1,032	55.2	11,409	55.3	622	54.7	9,129	55.3	2,902	55.1
**Age (years)**														
20-39	1,976	9.1	1,808	9.1	168	9.0	1,890	9.2	86	7.6	1,445	8.8	531	10.1
40-59	6,872	31.6	6,267	31.5	605	32.4	6,524	31.6	348	30.6	5,120	31.0	1,752	33.3
60-79	9,355	43.0	8,546	42.9	809	43.3	8,827	42.8	528	46.4	7,062	42.8	2,293	43.6
>= 80	3,570	16.4	3,283	16.5	287	15.4	3,395	16.5	175	15.4	2,881	17.5	689	13.1
**Obesity**														
No	20,463	94.0	18,661	93.8	1,802	96.4	19,366	93.9	1,097	96.5	15,533	94.1	4,930	93.6
Yes	1,310	6.0	1,243	6.2	67	3.6	1,270	6.2	40	3.5	975	5.9	335	6.4
**Diabetes**														
No	13,058	60.0	11,925	59.9	1,133	60.6	12,401	60.1	657	57.8	9,925	60.1	3,133	59.5
Yes	8,715	40.0	7,979	40.1	736	39.4	8,235	39.9	480	42.2	6,583	39.9	2,132	40.5
**Cardiovascular disease**														
No	10,391	47.7	9,400	47.2	991	53.0	9,858	47.8	533	46.9	7,866	47.7	2,525	48.0
Yes	11,382	52.3	10,504	52.8	878	47.0	10,778	52.2	604	53.1	8,642	52.4	2,740	52.0
**Chronic pulmonary disease**														
No	20,387	93.6	18,630	93.6	1,757	94.0	19,306	93.6	1,081	95.1	15,422	93.4	4,965	94.3
Yes	1,386	6.4	1,274	6.4	112	6.0	1,330	6.5	56	4.9	1,086	6.6	300	5.7
**Asthma**														
No	20,658	94.9	18,897	94.9	1,761	94.2	19,571	94.8	1,087	95.6	15,666	94.9	4,992	94.8
Yes	1,115	5.1	1,007	5.1	108	5.8	1,065	5.2	50	4.4	842	5.1	273	5.2
**Chronic kidney disease**														
No	20,179	92.7	18,420	92.5	1,759	94.1	19,106	92.6	1,073	94.4	15,255	92.4	4,924	93.5
Yes	1,594	7.3	1,484	7.5	110	5.9	1,530	7.4	64	5.6	1,253	7.6	341	6.5
**Chronic hematologic disease**														
No	21,438	98.5	19,597	98.5	1,841	98.5	20,313	98.4	1,125	98.9	16,245	98.4	5,193	98.6
Yes	335	1.5	307	1.5	28	1.5	323	1.6	12	1.1	263	1.6	72	1.4
**Chronic neurological disease**														
No	20,459	94.0	18,700	94.0	1,759	94.1	19,377	93.9	1,082	95.2	15,460	93.7	4,999	95.0
Yes	1,314	6.0	1,204	6.1	110	5.9	1,259	6.1	55	4.8	1,048	6.4	266	5.1

**Chronic liver disease**																
No	21,418	98.4	19,581	98.4	1,837	98.3	20,300	98.4	1,118	98.3	16,218	98.2	5,200	98.8
Yes	355	1.6	323	1.6	32	1.7	336	1.6	19	1.7	290	1.8	65	1.2
**Immunosuppression**														
No	20,579	94.5	18,792	94.4	1,787	95.6	19,485	94.4	1,094	96.2	15,571	94.3	5,008	95.1
Yes	1,194	5.5	1,112	5.6	82	4.4	1,151	5.6	43	3.8	937	5.7	257	4.9

Suppl. Table 2. Combined association of obesity, diabetes, and/or cardiovascular disease with death in adult and elderly patients hospitalized with severe COVID-19, excluding the cases of chronic pulmonary diseases and immunosuppression.

	**Main exposure variable**	**Death***	
		PR	95%CI
	None	1.00	
**Adults**	OB	1.38	1.07-1.79
20-59 years	OB + DM and/or CVD	1.86	1.49-2.32
	DM and/or CVD	1.16	1.03-1.32
	None	1.00	
**Elders**	OB	1.64	0.99-2.82
≥ 60 years	OB + DM and/or CVD	1.43	1.08-1.90
	DM and/or CVD	1.05	0.94-1.17

OB: obesity (BMI≥30 kg/m^2), DM: diabetes mellitus, CVD: cardiovascular disease, ICU: intensive care unit, PR: prevalence ratio, 95%CI: 95% confidence interval.
[#] Adjusted for sex, age in years, asthma, kidney disease, hematologic disease, neurological disease, and liver disease.
* Adjusted logistic regression model for death in adults (n=5889) and elders (n=8676).

Suppl. Table 3. Independent association of degrees of obesity with death in hospitalized adults with severe COVID-19, excluding the cases of chronic pulmonary disease and immunosuppression.

**Main exposure variable**	Death*	
	PR	95%CI
No obesity (< 30 kg/m^2)	1.00	
Obesity class I (≥ 30-34.9 kg/m^2)	1.39	1.09-1.77
Obesity class II (≥ 35-39.9 kg/m^2)	1.48	1.10-1.99
Obesity class III (≥ 40 kg/m^2)	1.79	1.34-2.41

Degrees of obesity defined by the WHO cutoff points.
PR: prevalence ratio, 95%CI: 95% confidence interval.
* Adjusted logistic regression model for death (n=5889).
[#] Adjusted for sex, age in years, diabetes mellitus, cardiovascular disease, asthma, kidney disease, hematologic disease, neurological disease, and liver disease.
