# Supplementary material for: Combined association of obesity and other cardiometabolic diseases with severe COVID-19 outcomes: a nationwide cross-sectional study of 21 773 Brazilian adult and elderly inpatients
Source: BMJ Open. 2021 Aug 9;11(8):e050739. doi: 10.1136/bmjopen-2021-050739 (PMC8354760; doi:10.1136/bmjopen-2021-050739)
Supplement: Author's manuscript [file bmjopen-2021-050739.draft_revisions.pdf]

BMJ Open is committed to open peer review. As part of this commitment we make the peer review history of every article we publish publicly available.

When an article is published we post the peer reviewers' comments and the authors' responses online. We also post the versions of the paper that were used during peer review. These are the versions that the peer review comments apply to.

The versions of the paper that follow are the versions that were submitted during the peer review process. They are not the versions of record or the final published versions. They should not be cited or distributed as the published version of this manuscript.

BMJ Open is an open access journal and the full, final, typeset and author-corrected version of record of the manuscript is available on our site with no access controls, subscription charges or pay-per-view fees (http://bmjopen.bmj.com).

If you have any questions on BMJ Open's open peer review process please email info.bmjopen@bmj.com

BMJ Open

Combined association of obesity and other cardiometabolic diseases with severe COVID-19 outcomes in 21,773 Brazilian adult and elderly inpatients

Journal:	*BMJ Open*
Manuscript ID	bmjopen-2021-050739
Article Type:	Original research
Date Submitted by the Author:	01-Mar-2021

Complete List of Authors:	Silva, Natanael; Oswaldo Cruz Foundation, Centre for Data and Knowledge Integration for Health<br>Silva, Rita de Cássia; Federal University of Bahia, School of Nutrition<br>Ferreira, Andrêa; Federal University of Bahia, Institute of Collective Health<br>Teixeira, Camila; Federal University of Bahia, Institute of Collective Health<br>Rocha, Aline; Federal University of Bahia, School of Nutrition<br>Alves, Flávia Jôse; Federal University of Bahia, Institute of Collective Health<br>Falcão, Ila; Oswaldo Cruz Foundation, Centre for Data and Knowledge Integration for Health<br>Pinto, Elizabete; Federal University of Reconcavo da Bahia, Center for Health Sciences<br>Santos, Carlos Antônio; Oswaldo Cruz Foundation, Centre for Data and Knowledge Integration for Health<br>Fiaccone, Rosemeire; Federal University of Bahia, Institute of Mathematics and Statistics<br>Ichihara, Maria Yury; Oswaldo Cruz Foundation, Centre for Data and Knowledge Integration for Health<br>Paixão, Enny; London School of Hygiene and Tropical Medicine Faculty of Epidemiology and Population Health<br>Barreto, Mauricio; Oswaldo Cruz Foundation, Centre for Data and Knowledge Integration for Health; Federal University of Bahia, Institute of Collective Health

Keywords:	COVID-19, INFECTIOUS DISEASES, INTENSIVE & CRITICAL CARE, DIABETES & ENDOCRINOLOGY, NUTRITION & DIETETICS, EPIDEMIOLOGY

SCHOLARONE™
Manuscripts

I, the Submitting Author has the right to grant and does grant on behalf of all authors of the Work (as defined in the below author licence), an exclusive licence and/or a non-exclusive licence for contributions from authors who are: i) UK Crown employees; ii) where BMJ has agreed a CC-BY licence shall apply, and/or iii) in accordance with the terms applicable for US Federal Government officers or employees acting as part of their official duties; on a worldwide, perpetual, irrevocable, royalty-free basis to BMJ Publishing Group Ltd ("BMJ") its licensees and where the relevant Journal is co-owned by BMJ to the co-owners of the Journal, to publish the Work in this journal and any other BMJ products and to exploit all rights, as set out in our licence.

The Submitting Author accepts and understands that any supply made under these terms is made by BMJ to the Submitting Author unless you are acting as an employee on behalf of your employer or a postgraduate student of an affiliated institution which is paying any applicable article publishing charge ("APC") for Open Access articles. Where the Submitting Author wishes to make the Work available on an Open Access basis (and intends to pay the relevant APC), the terms of reuse of such Open Access shall be governed by a Creative Commons licence – details of these licences and which Creative Commons licence will apply to this Work are set out in our licence referred to above.

Other than as permitted in any relevant BMJ Author's Self Archiving Policies, I confirm this Work has not been accepted for publication elsewhere, is not being considered for publication elsewhere and does not duplicate material already published. I confirm all authors consent to publication of this Work and authorise the granting of this licence.

For peer review only

Combined association of obesity and other cardiometabolic diseases with severe COVID-19 outcomes in 21,773 Brazilian adult and elderly inpatients

Natanael J Silva, MSc[1,2]; Rita C Ribeiro-Silva, PhD[1,2,3]; Andrêa JF Ferreira, MSc[1,2,4]; Camila SS Teixeira, MSc[1,2,4]; Aline S Rocha, MSc[1,2,3]; Flávia Jôse O Alves, MSc[1,2,4]; Ila R Falcão, PhD[2]; Elizabete J Pinto, PhD[2,5]; Carlos Antônio ST Santos, PhD[1,2,6]; Rosemeire L Fiaccone, PhD[1,2,7]; Maria Yury T Ichihara, MD PhD[1,2]; Enny S Paixão, PhD[1,2,8]; Mauricio L Barreto, MD PhD[1,2,4]

[1] Rede CoVida, Salvador, BA, Brazil

[2] Centre for Data and Knowledge Integration for Health, Oswaldo Cruz Foundation, Salvador, BA, Brazil

[3] School of Nutrition, Federal University of Bahia, Salvador, BA, Brazil

[4] Institute of Collective Health, Federal University of Bahia, Salvador, BA, Brazil

[5] Center for Health Sciences, Federal University of Recôncavo da Bahia, Santo Antônio de Jesus, BA, Brazil

[6] Department of Exact Sciences, State University of Feira de Santana, Feira de Santana, BA, Brazil

[7] Institute of Mathematics and Statistics, Federal University of Bahia, Salvador, BA, Brazil

[8] Epidemiology and Population Health, London School of Hygiene and Tropical Medicine, London, UK

Corresponding author: Natanael J Silva. Centre for Data and Knowledge Integration for Health, Oswaldo Cruz Foundation. Edf. Tecnocentro, Sl 315. Rua Mundo, 121. Trobogy, Salvador, BA, 41745-715, Brazil. Phone number: +55 79 99839-3991. Email: <silva_natanael@hotmail.com>.

Keywords: Obesity, COVID-19, SARS-CoV-2, Hospitalization, Mortality.

Word count: 2,702

For peer review only

Abstract

Objectives: To investigate the combined association of obesity, diabetes mellitus (DM), and cardiovascular disease (CVD) with severe COVID-19 outcomes in adult and elderly inpatients.

Design: Cross-sectional study based on registry data from Brazil's influenza surveillance system (Sistema de Informação de Vigilância Epidemiológica da Gripe– SIVEP-Gripe).

Setting: Public and private hospitals across Brazil.

Participants: Eligible population included 21,942 inpatients aged ≥20 years with positive RT-PCR test for SARS-CoV-2 until Jun 9th, 2020.

Main outcome measures: Severe COVID-19 were non-invasive and invasive mechanical ventilation use, ICU admission, and death. Multivariate analyses were conducted separately for adults (20-59 years) and elders (≥60 years) to test the combined association of obesity (without and with DM and/or CVD) and degrees of obesity with each outcome.

Results: A sample of 8,848 adults and 12,925 elders were included. Among adults, obesity with DM and/or CVD showed higher prevalence of invasive (PR 3.76, 95%CI 2.82-5.01) and non-invasive mechanical ventilation use (2.06, 1.58-2.69), ICU admission (1.60, 1.40-1.83), and death (1.79, 1.45-2.21) compared with the group without obesity, DM, and CVD. Among elders, obesity alone (without DM and CVD) had the highest prevalence of ICU admission (1.40, 1.07-1.82) and death (1.67, 1.00-2.80). A dose-response association was observed between degrees of obesity and death in adults: class I 1.32 (1.05-1.66), class II 1.41 (1.06-1.87), and class III 1.77 (1.35-2.33).

Conclusions: The combined association of obesity, diabetes, and/or cardiovascular disease with severe COVID-19 outcomes may be stronger in adults than in elders. The study also supports an independent relationship of obesity with severe outcomes, including a dose-response association between degrees of obesity and death in adults. These findings suggest important implications for the clinical care of patients with obesity and severe COVID-19 and support the inclusion of people with obesity in the high-risk group for protection from SARS-CoV-2.

For peer review only

Article summary

Strengths and limitations of this study:

- This is the first study that describes the independent and combined relationship of obesity with COVID-19 severity in Brazil, one of the biggest epicenters of the pandemic worldwide.
- The study was based on registry data of a large nationwide sample of patients admitted, due to severe SARS-CoV-2 infection, to public and private hospitals across the country.
- The large sample size and data availability allowed us to analyze the combined association of obesity, diabetes and cardiovascular disease with severe COVID-19 outcomes, separately by age groups and controlled by important confounding variables, e.g. underlying comorbidities.
- The cross-sectional study design does not allow causal inference, and generalization of results must be cautious since only hospitalized cases of severe COVID-19 were included.
- As the study used routinely collected data, which has not been designed primarily for research purposes, it may bring well-known limitations related to missing, underestimation, and potential misclassification.

For peer review only

Introduction

The coronavirus disease 2019 (COVID-19) pandemic, caused by the severe acute respiratory syndrome coronavirus 2 (SARS-CoV-2), as of 16 February 2021, has already reached more than 108.8 million infected people and more than 2.4 million deaths in all continents.[1] Individuals with advanced age and chronic diseases, including cardiometabolic diseases, are considered groups at major risk for complications and severe illness from COVID-19.[2,3] Obesity has been shown as an independent risk factor for COVID-19 disease.[4-6] High body mass index (BMI) has been mentioned as a significant risk factor for COVID-19, according to early clinical reports from China,[7] Italy,[8] France,[9] Mexico,[10] and United States of America.[11] Several studies have demonstrated that obesity is leading to considerably worse COVID-19 outcomes, especially greater risk of hospital and intensive care unit (ICU) admission, invasive mechanical ventilation, and death.[11-14]

The COVID-19 pandemic is rapidly spreading worldwide, especially in the Americas, where obesity is already a prevalent and important public health problem.[15-16] Brazil is currently one of the biggest epicenters of the COVID-19 pandemic worldwide, with more than 9.8 million cases and 239 thousand deaths until Feb 16, 2021.[1] In 2018, the prevalence of adult overweight and obesity in Brazil was estimated at 55.7% and 19.8%, respectively.[17] This obesogenic profile of the Brazilian population contributes, among other factors, to the high prevalence of obesity-related diseases such as type 2 diabetes mellitus (DM) and cardiovascular diseases (CVD), in the country.[18] The fact that individuals with obesity also have more comorbidity diseases, which are either risk factors for COVID-19 severity and death, makes obesity particularly ominous in COVID-19 disease.[10-13]

Several characteristics that can influence the clinical evolution of individuals infected with COVID-19, such as obesity, have been independently documented.[5-6,19] However, evidence is yet unclear on the combined effect that obesity and obesity-related comorbidities play in COVID-19 severity, especially, in different age groups. We aimed in this study to investigate the combined association of obesity, diabetes, and cardiovascular disease with mechanical ventilation use, ICU admission, and

For peer review only

death in a large sample of adult and elderly patients hospitalized with COVID-19 in Brazil. We also explored the independent association between degrees of obesity and the mentioned outcomes.

Methods

Study Design and Population

This is a cross-sectional study based on registry data from SIVEP-Gripe (Sistema de Informação de Vigilância Epidemiológica da Gripe), an influenza surveillance system of Brazil's Ministry of Health. The study used the publicly available dataset of SIVEP-Gripe, which includes de-identified data on cases of severe acute respiratory syndrome across public and private hospitals in Brazil.[20] These data were obtained through the Rede CoVida's integrated data platform that has been built with official, open, and authorized data for the production of knowledge about the COVID-19 pandemic. Our study population was composed of patients aged 20 years or older, hospitalized for severe acute respiratory syndrome, with positive RT-PCR test for SARS-CoV-2, and final diagnosis for COVID-19 until Jun 9th, 2020. Only cases with complete data on demographic characteristics and comorbidities and plausible BMI values were included in the study.

The study was conducted according to the guidelines laid down in the Declaration of Helsinki. As the study exclusively used publicly available de-identified data, ethics approval by a research ethics committee and informed consent are waived per Resolution n. 466/2012 of the National Health Council of Brazil's Commission of Ethics in Research.

Exposure Variable

Obesity was defined as BMI equal to or greater than 30 kg/m^2, according to the cutoff points proposed by the World Health Organization[21] and the Pan American Health Organization[22] for adults and elders, respectively. BMI was calculated by health professionals in the hospital from directly measured or patient self-reported height and weight. Guidelines for the collection and analysis of anthropometric data in health services have been previously standardized by the Ministry of Health.[23]

For peer review only

BMI values <12 or >70 kg/m^2 were considered implausible and excluded.[24] The existence of diabetes and CVD were based on patient or family's report or medical diagnosis.

We created a polytomous four-category variable to evaluate the separate and combined exposure of obesity, diabetes and cardiovascular disease: none/reference (no existence of obesity, diabetes and cardiovascular disease), OB (only existence of obesity), OB + DM and/or CVD (existence of obesity with diabetes and/or cardiovascular disease), and DM and/or CVD (existence of diabetes and/or cardiovascular disease). We also analyzed obesity in adults according to the following degrees of severity[21]: no obesity (< 30 kg/m^2), obesity class I (≥ 30-34.9 kg/m^2), obesity class II (≥ 35-39.9 kg/m^2), and obesity class III (≥ 40 kg/m^2).

Outcome Variables

The severe COVID-19 outcomes were mechanical ventilation use, ICU admission, and death. Information on the use of mechanical ventilation by the patient was obtained and analyzed as a polytomous three-category variable (no use/ use of non-invasive ventilation/ use of invasive ventilation). ICU admission was obtained and analyzed as a dichotomous variable (no/ yes). Death was analyzed as a dichotomous variable based on the patient's endpoint outcome (cure/ death).

Covariates

Demographic and comorbidity information were selected as descriptive and confounding variables.[2] Age in years was calculated from birth and notification dates. Sex was obtained as a dichotomous variable (female/ male). The preexistence of each comorbidity was also obtained as a dichotomous variable (no/ yes): chronic pulmonary disease, asthma, chronic kidney disease, chronic hematologic disease, neurological disease, chronic liver disease, and immunodeficiency/ immunosuppression.

Statistical Analysis

All analyses were subdivided into adults (≥ 20 and < 60 years) and elders (≥ 60 years). For descriptive analyses, absolute and relative frequencies were calculated for the demographic and comorbidity variables according to the main exposure variable. Multinomial logistic regression models were

For peer review only

conducted to test the association of obesity (without and with diabetes and/or CVD) with non-invasive and invasive mechanical ventilation use. To test the association of this exposure variable with ICU admission and death, simple logistic regression models were performed. Same models were analyzed considering the degree of obesity as the main exposure variable for adults. Crude and adjusted estimates were interpreted based on the prevalence ratio (PR) and 95% confidence intervals (95%CI). Adjusted models included the following list of confounding variables: sex, age (years), and the preexistence of chronic pulmonary disease, asthma, kidney disease, hematologic disease, neurological disease, liver disease, and immunodeficiency/ immunosuppression. All analyses were performed using Stata version 15.1 (Stata Corporation, College Station, USA) and R software version 3.6.1 (R Foundation for Statistical Computing, Austria) with the package "prLogistic".

Patient and Public Involvement

As the study exclusively used publicly available de-identified data, it was not possible to involve patients or the public in the design, or conduct, or reporting, or dissemination plans of our research.

Results

During the study period, 21,942 individuals registered in the SIVEP-Gripe were ≥ 20 years old, hospitalized, tested positive for SARS-CoV-2, and had complete demographic and comorbidity information (**Figure 1**). Of these, 169 (0.8%) were excluded due to implausible values of BMI. Of the 21,773 individuals included in the study, 8,848 (40.3%) were adults aged between 20-59 years, and 12,925 (59.6%) were elders aged 60 years or older. Since some patients were still hospitalized on the study endpoint date, information for some outcomes were incomplete. The study population included in the analysis of each outcome were 8,075 adults and 11,829 elders for mechanical ventilation, 8,414 adults and 12,222 for ICU admission, and 6,565 adults and 9,943 elders for death.

The prevalence of obesity was 9.7% in adults and 3.5% in elders. The frequency of obesity without and with DM and/or CVD was respectively 4.6% and 5.1% in adults and 0.7% and 2.8% in elders. Non-invasive and invasive mechanical ventilation were respectively required by 45.0% and 21.2%

For peer review only

of adults and 47.0% and 30.0% of elders. ICU admission was needed by 35.4% of adults and 43.6% of elders. Death occurred in 31.1% of adult and 63.0% of elderly patients (**Table 1-2**).

In the adjusted analyses, adults with obesity alone (without DM and CVD) had an increased prevalence of invasive (PR 2.69, 95%CI 1.98-3.65) and non-invasive mechanical ventilation need (PR 2.13, 95%CI 1.64-2.78), ICU admission (PR 1.31, 95%CI 1.13-1.53), and death (PR 1.33, 95%CI 1.05-1.69) when compared with the group without obesity, DM, and CVD. Obesity with DM and/or CVD presented an even higher prevalence of invasive mechanical ventilation (PR 3.76, 95%CI 2.82-5.01) and non-invasive ventilation use (PR 2.06, 95%CI 1.58-2.69), ICU admission (PR 1.60, 95%CI 1.40-1.83), and death in adults (PR 1.79, 95%CI 1.45-2.21) (**Table 3**).

Among elders, obesity without DM and CVD increased independently the prevalence of ICU admission by 40% (95%CI 1.07-1.82) and death by 67% (1.00-2.80). Obesity with DM and/or CVD increased the prevalence of invasive mechanical ventilation need (PR 1.66, 95%CI 1.22-2.27), ICU admission (PR 1.37, 95%CI 1.19-1.59), and death (PR 1.39, 95%CI 1.07-1.80) (**Table 3**).

In the analyses by the degree of obesity, we did not observe much difference in the prevalence of adverse outcomes, except for the prevalence of death that increased with the severity of obesity: Class I 1.32 (95%CI 1.05-1.66), Class II 1.41 (1.06-1.87), and Class III 1.77 (1.35-2.33) (**Table 4**).

Discussion

This is the first study that describe the relationship of obesity and COVID-19 in Brazil, based on a large nationwide sample of adults and elders tested positive for SARS-CoV-2 and admitted to public and private hospitals. Our results highlights that obesity with DM and/or CVD had higher rates of invasive mechanical ventilation use, ICU admission, and death in adults, while obesity alone (without DM and CVD) presented higher rates of ICU admission and death among elders. The study also supports the independent association of obesity with the analyzed outcomes and a dose-response association between degrees of obesity and death in adults.

For peer review only

Some mechanisms related to the role of obesity and related diseases in worsening the clinical condition of patients affected by SARS-CoV-2 have been pointed out: i) greater body weight causes less elasticity of the chest wall and less total compliance of the respiratory system, leading to a restriction of the ventilation and the excursion of the diaphragm, making difficult the airway management in patients with obesity;[25] ii) obesity is associated with sleep apnea syndrome and chronic obstructive pulmonary disease, which lead to surfactant dysfunction and impede the proper functioning of the airways;[26] iii) obesity is a metabolic and inflammatory disease, which is associated with the development or worsening of other chronic and endocrine comorbidities (e.g. type 2 diabetes, hypertension, dyslipidemia and CVD) that can modify innate and adaptive immune responses, making the immune system more vulnerable to infections and less responsive to antivirals and antimicrobial drugs;[16] iv) glycemic decompensation, common in patients with obesity, is associated with impaired ventilation function.[26]

It is important to note that the COVID-19 pandemic imposes a double burden of disease, especially among the elderly individuals, since the prevalence of diabetes, hypertension, cardiovascular diseases, and other comorbidities associated with COVID-19 severity increases with age.[3,27] However, our study suggests that obesity combined with diabetes and/or cardiovascular disease may offer higher risk of COVID-19 severity for adults although the overall prevalence of diseases and rates of ICU admission and mortality were higher in elders. Obesity alone seemed to provide higher risk of severe outcomes, especially death, in elders.

Few studies to-date have explored the combined and additional effect of obesity on COVID-19 severity.[13,28] A study investigated the patterns of multimorbidity among fatal cases of COVID-19 in Colombia.[28] Similar to our study, the authors found that obesity alone or with other diseases was associated with a higher risk of COVID-19 fatality among young people. Furthermore, a population-based study in Mexico observed that the addition of obesity to any number of comorbidities significantly increased the risk of COVID-19 lethality.[13] Using a causally ordered mediation analysis,

For peer review only

this study also found that 49.5% of the effect of diabetes on COVID-19 lethality was mediated by obesity, particularly in early-onset cases < 40 years of age.

Other studies also suggest that obesity is independently associated with severe outcomes of COVID-19, regardless of age and other associated comorbidities.[11-14] A large study in Mexico[13] showed that patients with obesity had higher rates of ICU admission and were more likely to be intubated in relation to patients without obesity. This study also found a five-fold increased risk of mortality due to COVID-19 in patients with obesity.[13] In a hospital-based study in France, it was observed that BMI > 35 kg/m^2 was associated with the need for invasive mechanical ventilation.[14]

Few studies to-date have similarly found a dose-response association between degrees of obesity and COVID-19 death.[29] Based on care records of 17,278,392 UK adults, the study showed that the risk of COVID-19 death increases independently with the degree of obesity: 30-34.9 kg/m^2 (HR 1.05), 35-39.9 kg/m^2 (1.40), and ≥40 kg/m^2 (2.66).[29] Other studies have evidenced the association of obesity with COVID-19 complications and death among adults.[12,30] A hospital-based study in New York City showed that morbid obesity (BMI ≥ 40 kg/m^2) is strongly and independently associated with death in hospitalized patients younger than 50 years.[30] Another study in New York City found a similar dose-response relationship between degrees of obesity and acute and critical care.[12] Patients less than 60 years old with BMI between 30 and 34.9 kg/m^2 (obesity class I) were 2.0 and 1.8 times more likely to be respectively admitted for acute care (general hospital admission) and critical care (ICU admission or invasive ventilator) compared to individuals with BMI < 30 kg/m^2. Patients of the same age group with BMI ≥ 35 kg/m^2 (obesity class II and III) showed 2.2 and 3.6 more chances of being hospitalized for acute and critical care, respectively.[12]

Strengths and limitations

One of the greatest strengths of the study was the use of SIVEP-Gripe dataset. Because severe acute respiratory syndrome is a condition of compulsory notification in both public and private hospitals,[31] we have a nationwide representative sample of patients hospitalized for severe COVID-19 in Brazil. In addition, the large sample sizes allowed us to analyze adults and elders separately, as well as the

For peer review only

degrees of obesity which dose-response association with death was evidenced. The availability of important confounding variables (sex, age, and preexisting comorbidities) to control the estimated associations, as well as hospital outcomes and mortality of COVID-19, was another differential of the study. Only patients with positive RT-PCR test for SARS-CoV-2 and final diagnosis for COVID-19 were included which gives greater precision on the studied population.

The study also has some limitations that must be considered. Because this is a cross-sectional study, a causal association cannot be inferred. As we used routinely collected data, which has not been designed primarily for research purposes, it may bring well-known limitations related to missing, underestimation, and potential misclassification. Obesity prevalence may have been underestimated due to the completeness of obesity and BMI data (42%). Previous studies using SIVEP-Gripe data have also found a low prevalence of obesity in this population.[32,33] Better routine collection of height and weight data is still needed in clinical practice. Also, we believe that health professionals have adopted more the one method to collect weight and height information for BMI calculation, such as the patient's self-report and direct measure. Therefore, in addition to BMI which implausible values were checked and excluded, the classification of obesity was also confirmed from a dichotomous variable on the presence of obesity (no/yes). Although it is known that BMI does not distinguish between fat and lean body mass, and thus may lead to misclassification bias, BMI has been shown as a strong predictor of excess body fat and has been widely used in epidemiological studies.[15] Data on ethnicity/race was very incomplete, and thus was not included in the analysis. Additional studies are needed to further explore the relationship between socioeconomic characteristics and obesity in severe disease. Finally, the generalization of results must be cautious since the study included only hospitalized cases of COVID-19.

Conclusions

The combined association of obesity, diabetes, and/or cardiovascular disease with severe COVID-19 outcomes, especially ICU admission and death, may be stronger in adult than in elderly inpatients. The study also supports an independent relationship of obesity with the severe outcomes, including a

For peer review only

dose-response association between degrees of obesity and death in adults. These findings suggest important implications for the clinical care of patients with obesity and severe COVID-19 and support the inclusion of people with obesity, independently of other preexisting comorbidities and age, in the high-risk group for protection from SARS-CoV-2 infection.

Acknowledgments

The authors thank the members of Rede CoVida's Epidemiology & Information Group for the work of identifying and collecting data related to COVID-19.

Author Contributions

NJS, RCRS, and RLF designed the study and analysis strategy. NJS, CASTS, and MYTI obtained, documented, and described the data. AJFF, CSST, ASR, FJOA, and IRF carried out the literature search. NJS and EJP performed the data analysis. NJS, RCRS, AJFF, CSST, ASR, FJOA, IRF, ESP and MLB contributed to data interpretation. NJS, AJFF, CSST, ASR, FJOA, and IRF drafted the manuscript. RCRS, ESP, MYTI, and MLB critically revised the manuscript. All authors read and approved the final manuscript.

Funding

All authors are affiliated to the Centre for Data and Knowledge Integration for Health (CIDACS) that is funded and supported by MCTI/ CNPq/ MS/ SCTIE/ Decit/ Bill & Melinda Gates Foundation's GCE Brazil (OPP1142172), Wellcome Trust (202912/Z/16/Z), the Brazilian Health Surveillance Secretariat, Ministry of Health, Bahia State, Research Support Foundation of the State of Bahia (FAPESB), the Research and Project Funding Agency (FINEP), and the Secretariat of Science and Technology of the State of Bahia (SECTI). Dr Paixão is a fellow supported by the Wellcome Trust (213589/Z/18/Z).

Competing Interests

None declared.

Patient Consent for Publication

Not required.

For peer review only

Data Availability Statement

Data is freely available without restriction at https://opendatasus.saude.gov.br/dataset/bd-srag-2020.

Code book and analytic code will be made available upon request from the corresponding author.

For peer review only

References

1. World Health Organization. WHO Coronavirus Disease (COVID-19) Dashboard. https://covid19.who.int/. Accessed February 16, 2020.
2. Centers for Disease Control and Prevention. Coronavirus Disease 2019 (COVID-19). People Who Are at Increased Risk for Severe Illness. https://www.cdc.gov/coronavirus/2019-ncov/need-extra-precautions/people-at-increased-risk.html. Accessed July 21, 2020.
3. Liu H, Chen S, Liu M, Nie H, Lu H. Comorbid Chronic Diseases are Strongly Correlated with Disease Severity among COVID-19 Patients: A Systematic Review and Meta-Analysis. *Aging Dis*. 2020;11(3):668-678. doi:10.14336/ad.2020.0502.
4. Alberca RW, Oliveira LM, Branco ACCC, Pereira NZ, Sato MN. Obesity as a risk factor for COVID-19: an overview [published online June 15, 2020]. *Crit Rev Food Sci Nutr*. doi:10.1080/10408398.2020.1775546.
5. Sattar N, McInnes IB, McMurray JJV. Obesity Is a Risk Factor for Severe COVID-19 Infection: Multiple Potential Mechanisms. *Circulation*. 2020;142(1):4-6. doi:10.1161/circulationaha.120.047659.
6. Caussy C, Wallet F, Laville M, Disse E. Obesity is Associated with Severe Forms of COVID-19. *Obesity (Silver Spring)*. 2020;28(7):1175. doi:10.1002/oby.22842.
7. Li Q, Guan X, Wu P, et al. Early Transmission Dynamics in Wuhan, China, of Novel Coronavirus-Infected Pneumonia. *N Engl J Med*. 2020;382(13):1199-1207. doi:10.1056/nejmoa2001316.
8. Grasselli G, Zangrillo A, Zanella A, et al. Baseline Characteristics and Outcomes of 1591 Patients Infected with SARS-CoV-2 Admitted to ICUs of the Lombardy Region, Italy. *JAMA*. 2020;323(16):1574-1581. doi:10.1001/jama.2020.5394.
9. Caussy C, Pattou F, Wallet F, et al. Prevalence of obesity among adult inpatients with COVID-19 in France. *Lancet Diabetes Endocrinol*. 2020;8(7):562-564. doi:10.1016/s2213-8587(20)30160-1.
10. Hernández-Garduño E. Obesity is the comorbidity more strongly associated for Covid-19 in Mexico. A case-control study. *Obes Res Clin Pract*. 2020;14(4):375-379. doi:10.1016/j.orcp.2020.06.001.
11. Hajifathalian K, Kumar S, Newberry C, et al. Obesity is associated with worse outcomes in COVID-19: Analysis of Early Data From New York City. *Obesity (Silver Spring)*. 2020;28(9):1606-1612. doi:10.1002/oby.22923.
12. Lighter J, Phillips M, Hochman S, et al. Obesity in patients younger than 60 years is a risk factor for Covid-19 hospital admission. *Clin Infect Dis*. 2020;71(15):896-897. doi:10.1093/cid/ciaa415.
13. Bello-Chavolla OY, Bahena-López JP, Antonio-Villa NE, et al. Predicting Mortality Due to SARS-CoV-2: A Mechanistic Score Relating Obesity and Diabetes to COVID-19 Outcomes in Mexico. *J Clin Endocrinol Metab*. 2020;105(8):dgaa346. doi:10.1210/clinem/dgaa346.
14. Simonnet A, Chetboun M, Poissy J, et al. High Prevalence of Obesity in Severe Acute Respiratory Syndrome Coronavirus-2 (SARS-CoV-2) Requiring Invasive Mechanical Ventilation. *Obesity (Silver Spring)*. 2020;28(7):1195-1199. doi:10.1002/oby.22831.
15. NCD Risk Factor Collaboration (NCD-RisC). Worldwide trends in body-mass index, underweight, overweight, and obesity from 1975 to 2016: a pooled analysis of 2416 population-based measurement studies in 128.9 million children, adolescents, and adults. *Lancet*. 2017;390(10113):2627-2642. doi:10.1016/s0140-6736(17)32129-3.
16. GBD 2015 Obesity Collaborators. Health Effects of Overweight and Obesity in 195 Countries over 25 Years. *N Engl J Med*. 2017;377(1):13-27. doi:10.1056/nejmoa1614362.
17. Brasil. Ministério da Saúde. Vigitel Brasil 2018: vigilância de fatores de risco e proteção para doenças crônicas por inquérito telefônico. Brasília, DF: Ministério da Saúde; 2019. https://portalarquivos2.saude.gov.br/images/pdf/2019/julho/25/vigitel-brasil-2018.pdf. Accessed July 10, 2020.

1

18. GBD 2016 Brazil Collaborators. Burden of disease in Brazil, 1990-2016: a systematic subnational analysis for the Global Burden of Disease Study 2016. *Lancet*. 2018;392(10149):760-775. doi:10.1016/s0140-6736(18)31221-2.
19. Flint SW, Tahrani AA. COVID-19 and obesity-lack of clarity, guidance, and implications for care. *Lancet Diabetes Endocrinol*. 2020;8(6):474-475. doi:10.1016/s2213-8587(20)30156-x.
20. Brasil. Ministério da Saúde. Severe Acute Respiratory Syndrome dataset - including COVID-19 data. 2020. https://opendatasus.saude.gov.br/dataset/bd-srag-2020. Accessed July 11, 2020.
21. World Health Organization. Physical status: the use and interpretation of anthropometry. Geneva: WHO; 1995. https://apps.who.int/iris/handle/10665/37003. Accessed July 11, 2020.
22. Organización Panamericana de la Salud. División de Promoción y Protección de la Salud (HPP). Encuesta sobre Salud, Bienestar y Envejecimiento (SABE) en América Latina el Caribe: Informe Preliminar (Internet). In: XXXVI Reunión del Comité asesor de investigaciones em Salud; 9-11 jun 2001; Kingston: OPAS, 2002. http://envejecimiento.csic.es/documentos/documentos/paho-salud-01.pdf. Accessed July 11, 2020.
23. Brasil. Ministério da Saúde. Guidelines for the Collection and Analysis of Anthropometric Data in Health Services. Technical Standard for the Food and Nutrition Surveillance System. Brasília, DF: Ministério da Saúde; 2011.
24. Cheng FW, Gao X, Mitchell DC, et al. Body mass index and all-cause mortality among older adults. *Obesity (Silver Spring)*. 2016;24(10):2232-39. doi:10.1002/oby.21612.
25. Honce R, Schultz-Cherry S. Impact of Obesity on Influenza A Virus Pathogenesis, Immune Response, and Evolution. *Front Immunol*. 2019;10:1071. doi:10.3389/fimmu.2019.01071.
26. Puig-Domingo M, Marazuela M, Giustina A. COVID-19 and endocrine diseases. A statement from the European Society of Endocrinology. *Endocrine*. 2020;68(1):2-5. doi:10.1007/s12020-020-02294-5.
27. Docherty AB, Harrison EM, Green CA, et al. Features of 20 133 UK patients in hospital with covid-19 using the ISARIC WHO Clinical Characterisation Protocol: prospective observational cohort study. *BMJ*. 2020;369:m1985. doi:10.1136/bmj.m1985.
28. Fernández-Niño JA, Guerra-Gómez JA, Idrovo AJ. Multimorbidity patterns among COVID-19 deaths: proposal for the construction of etiological models. *Rev Panam Salud Publica*. 2020;44:e166. doi:10.26633/RPSP.2020.166.
29. Williamson EJ, Walker AJ, Bhaskaran K, et al. Factors associated with COVID-19-related death using OpenSAFELY. *Nature*. 2020;584(7821):430-436. doi:10.1038/s41586-020-2521-4.
30. Klang E, Kassim G, Soffer S, et al. Morbid Obesity as an Independent Risk Factor for COVID-19 Mortality in Hospitalized Patients Younger than 50. *Obesity (Silver Spring)*. 2020;28(9):1595-1599. doi:10.1002/oby.22913.
31. Cantarino L, Merchan-Hamann E. Influenza in Brazil: surveillance pathways. *J Infect Dev Ctries*. 2016;10(1):13-23. doi:10.3855/jidc.7135.
32. de Souza WM, Buss LF, Candido DDS, et al. Epidemiological and clinical characteristics of the COVID-19 epidemic in Brazil. *Nat Hum Behav*. 2020;4(8):856-865. doi:10.1038/s41562-020-0928-4.
33. Baqui P, Bica I, Marra V, Ercole A, van der Schaar M. Ethnic and regional variations in hospital mortality from COVID-19 in Brazil: a cross-sectional observational study. *Lancet Glob Health*. 2020;8(8):e1018-e1026. doi: 10.1016/S2214-109X(20)30285-0.

Figure legends

Figure 1. Selection of the study population from SIVEP-Gripe.

SIVEP-Gripe: Influenza Epidemiological Surveillance Information System.

For peer review only

Table 1. Demographic characteristics, comorbidities, hospitalization outcomes and death according to the combined exposure of obesity, diabetes, and/or cardiovascular diseases in adults with severe COVID-19.

	Total		None		OB		OB + DM and/or CVD		DM and/or CVD	
	n	%	n	%	n	%	n	%	n	%
Overall	8848	100.0	3161	35.7	409	4.6	452	5.1	4826	54.6
**Sex**										
Female	3774	42.7	1511	40.0	165	4.4	199	5.3	1899	50.3
Male	5074	57.4	1650	32.5	244	4.8	253	5.0	2927	57.7
**Age**										
< 40 years	1976	22.3	1064	53.9	188	9.5	102	5.2	622	31.5
≥ 40 years	6872	77.7	2097	30.5	221	3.2	350	5.1	4204	61.2
**Diabetes mellitus**										
No	5839	66.0	3161	54.1	409	7.0	193	3.3	2076	35.6
Yes	3009	34.0	..	..	..	..	259	8.6	2750	91.4
**Cardiovascular disease**										
No	5263	59.5	3161	60.1	409	7.8	112	2.1	1581	30.0
Yes	3585	40.5	..	..	..	..	340	9.5	3245	90.5
**Chronic pulmonary disease**										
No	8502	96.1	2969	34.9	388	4.6	435	5.1	4710	55.4
Yes	346	3.9	192	55.5	21	6.1	17	4.9	116	33.5
**Asthma**										
No	8184	92.5	2728	33.3	383	4.7	414	5.1	4659	56.9
Yes	664	7.5	433	65.2	26	3.9	38	5.7	167	25.2
**Chronic kidney disease**										
No	8297	93.8	2958	35.7	399	4.8	434	5.2	4506	54.3
Yes	551	6.2	203	36.8	10	1.8	18	3.3	320	58.1
**Chronic hematologic disease**										
No	8710	98.4	3081	35.4	406	4.7	445	5.1	4778	54.9
Yes	138	1.6	80	58.0	3	2.2	7	5.1	48	34.8
**Chronic neurological disease**										
No	8588	97.1	3014	35.1	406	4.7	442	5.2	4726	55.0
Yes	260	2.9	147	56.5	3	1.2	10	3.9	100	38.5
**Chronic liver disease**										
No	8684	98.2	3083	35.5	406	4.7	443	5.1	4752	54.7
Yes	164	1.9	78	47.6	3	1.8	9	5.5	74	45.1
**Immunosuppression**										
No	8276	93.5	2777	33.6	393	4.8	440	5.3	4666	56.4
Yes	572	6.5	384	67.1	16	2.8	12	2.1	160	28.0
**Mechanical ventilation***										
No	2727	33.8	1144	42.0	93	3.4	88	3.2	1402	51.4
Non-invasive	3634	45.0	1178	32.4	192	5.3	190	5.2	2074	57.1
Invasive	1714	21.2	529	30.9	101	5.9	150	8.8	934	54.5
**ICU admission***										
No	5438	64.6	2025	37.2	235	4.3	222	4.1	2956	54.4
Yes	2976	35.4	1007	33.8	163	5.5	216	7.3	1590	53.4
**Death***										
No	4525	68.9	1699	37.6	211	4.7	200	4.4	2415	53.4
Yes	2040	31.1	640	31.4	92	4.5	140	6.9	1168	57.3

OB: obesity (BMI≥30 kg/m^2), DM: diabetes mellitus, CVD: cardiovascular disease, ICU: intensive care unit.
* Mechanical ventilation (n=8075), ICU admission (n=8414), and death (n=6565).

For peer review only

Table 2. Demographic characteristics, comorbidities, hospitalization outcomes and death according to the combined exposure of obesity, diabetes, and/or cardiovascular diseases in elders with severe COVID-19.

	Total		None		OB		OB + DM and/or CVD		DM and/or CVD	
	n	%	n	%	n	%	n	%	n	%
Overall	12925	100.0	2837	21.9	91	0.7	358	2.8	9639	74.6
**Sex**										
Female	5968	46.2	1232	20.6	52	0.9	209	3.5	4475	75.0
Male	6957	53.8	1605	23.1	39	0.6	149	2.1	5164	74.2
**Age**										
< 80 years	9355	72.4	2011	21.5	77	0.8	309	3.3	6958	74.4
≥ 80 years	3570	27.6	826	23.1	14	0.4	49	1.4	2681	75.1
**Diabetes mellitus**										
No	7219	55.9	2837	39.3	91	1.3	125	1.7	4166	57.7
Yes	5706	44.2	..	..	..	..	233	4.1	5473	95.9
**Cardiovascular disease**										
No	5128	39.7	2837	55.3	91	1.8	58	1.1	2142	41.8
Yes	7797	60.3	..	..	..	..	300	3.9	7497	96.2
**Chronic pulmonary disease**										
No	11885	92.0	2494	21.0	85	0.7	325	2.7	8981	75.6
Yes	1040	8.1	343	33.0	6	0.6	33	3.2	658	63.3
**Asthma**										
No	12474	96.5	2687	21.5	90	0.7	336	2.7	9361	75.0
Yes	451	3.5	150	33.3	1	0.2	22	4.9	278	61.6
**Chronic kidney disease**										
No	11882	91.9	2608	22.0	85	0.7	311	2.6	8878	74.7
Yes	1043	8.1	229	22.0	6	0.6	47	4.5	761	73.0
**Chronic hematologic disease**										
No	12728	98.5	2751	21.6	91	0.7	354	2.8	9532	74.9
Yes	197	1.5	86	43.7	0	0.0	4	2.0	107	54.3
**Chronic neurological disease**										
No	11871	91.9	2511	21.2	89	0.8	338	2.9	8933	75.3
Yes	1054	8.2	326	30.9	2	0.2	20	1.9	706	67.0
**Chronic liver disease**										
No	12734	98.5	2777	21.8	87	0.7	353	2.8	9517	74.7
Yes	191	1.5	60	31.4	4	2.1	5	2.6	122	63.9
**Immunosuppression**										
No	12303	95.2	2558	20.8	87	0.7	342	2.8	9316	75.7
Yes	622	4.8	279	44.9	4	0.6	16	2.6	323	51.9
**Mechanical ventilation***										
No	2725	23.0	626	23.0	18	0.7	70	2.6	2011	73.8
Non-invasive	5557	47.0	1164	21.0	38	0.7	141	2.5	4214	75.8
Invasive	3547	30.0	767	21.6	29	0.8	133	3.8	2618	73.8
**ICU admission***										
No	6898	56.4	1578	22.9	41	0.6	168	2.4	5111	74.1
Yes	5324	43.6	1107	20.8	44	0.8	181	3.4	3992	75.0
**Death***										
No	3684	37.1	823	22.3	21	0.6	95	2.6	2745	74.5
Yes	6259	63.0	1407	22.5	43	0.7	173	2.8	4636	74.1

OB: obesity (BMI≥30 kg/m^2), DM: diabetes mellitus, CVD: cardiovascular disease, ICU: intensive care unit.
* Mechanical ventilation (n=11829), ICU admission (n=12222), and death (n=9943).

For peer review only

Table 3. Combined association of obesity, diabetes, and/or cardiovascular disease with non-invasive and invasive mechanical ventilation use, intensive care unit admission, and death in adult and elderly patients hospitalized with severe COVID-19.

	Main exposure variable	Non-invasive mechanical ventilation*				Invasive mechanical ventilation*			
		Crude model		Adjusted model #		Crude model		Adjusted model #	
		PR	95%CI	PR	95%CI	PR	95%CI	PR	95%CI
	None	1.00		1.00		1.00		1.00	
**Adults**	OB	2.00	1.54-2.60	2.13	1.64-2.78	2.35	1.74-3.17	2.69	1.98-3.65
20-59 years	OB + DM and/or CVD	2.10	1.61-2.73	2.06	1.58-2.69	3.69	2.78-4.89	3.76	2.82-5.01
	DM and/or CVD	1.44	1.29-1.60	1.35	1.20-1.51	1.44	1.26-1.64	1.32	1.14-1.52
	None	1.00		1.00		1.00		1.00	
**Elders**	OB	1.14	0.64-2.01	1.22	0.69-2.16	1.31	0.72-2.39	1.43	0.78-2.61
≥ 60 years	OB + DM and/or CVD	1.08	0.80-1.47	1.15	0.84-1.55	1.55	1.14-2.11	1.66	1.22-2.27
	DM and/or CVD	1.13	1.01-1.26	1.14	1.01-1.27	1.06	0.94-1.20	1.10	0.97-1.24
		**ICU admission****				**Death*****			
		Crude model		Adjusted model #		Crude model		Adjusted model #	
		PR	95%CI	PR	95%CI	PR	95%CI	PR	95%CI
	None	1.00		1.00		1.00		1.00	
**Adults**	OB	1.23	1.08-1.40	1.31	1.13-1.53	1.11	0.92-1.33	1.33	1.05-1.69
20-59 years	OB + DM and/or CVD	1.48	1.33-1.65	1.60	1.40-1.83	1.50	1.30-1.74	1.79	1.45-2.21
	DM and/or CVD	1.05	0.99-1.12	1.03	0.95-1.12	1.19	1.10-1.29	1.16	1.03-1.30
	None	1.00		1.00		1.00		1.00	
**Elders**	OB	1.26	1.02-1.55	1.40	1.07-1.82	1.06	0.89-1.27	1.67	1.00-2.80
≥ 60 years	OB + DM and/or CVD	1.26	1.13-1.41	1.37	1.19-1.59	1.02	0.93-1.12	1.39	1.07-1.80
	DM and/or CVD	1.06	1.01-1.12	1.11	1.04-1.18	1.00	0.96-1.03	1.05	0.95-1.16

OB: obesity (BMI≥30 kg/m^2), DM: diabetes mellitus, CVD: cardiovascular disease, ICU: intensive care unit.
* Crude and adjusted multinomial logistic regression models for mechanical ventilation use in adults (n=8075) and elders (n=11829).
** Crude and adjusted logistic regression models for ICU admission in adults (n= 8414) and elders (n=12222).
*** Crude and adjusted logistic regression models for death in adults (n=6565) and elders (n=9943).
# Adjusted for sex, age in years, pulmonary disease, asthma, kidney disease, hematologic disease, neurological disease, liver disease, and immunosuppression.

For peer review only

Table 4. Independent association of degrees of obesity with non-invasive and invasive mechanical ventilation, intensive care unit admission, and death in hospitalized adults with severe COVID-19.

Main exposure variable	Non-invasive mechanical ventilation*				Invasive mechanical ventilation*			
	Crude model		Adjusted model #		Crude model		Adjusted model #	
	PR	95%CI	PR	95%CI	PR	95%CI	PR	95%CI
No obesity (< 30 kg/m²)	1.00		1.00		1.00		1.00	
Obesity class I (≥ 30-34.9 kg/m²)	1.78	1.35-2.33	1.91	1.45-2.51	2.59	1.93-3.47	3.00	2.22-4.05
Obesity class II (≥ 35-39.9 kg/m²)	1.44	1.04-2.00	1.58	1.14-2.19	2.10	1.47-2.99	2.47	1.72-3.54
Obesity class III (≥ 40 kg/m²)	1.70	1.19-2.44	1.88	1.31-2.69	2.51	1.71-3.70	3.00	2.03-4.45
	ICU admission**				Death**			
	Crude model		Adjusted model #		Crude model		Adjusted model #	
	PR	95%CI	PR	95%CI	PR	95%CI	PR	95%CI
No obesity (< 30 kg/m²)	1.00		1.00		1.00		1.00	
Obesity class I (≥ 30-34.9 kg/m²)	1.31	1.17-1.47	1.42	1.23-1.64	1.11	0.94-1.31	1.32	1.05-1.66
Obesity class II (≥ 35-39.9 kg/m²)	1.34	1.16-1.54	1.46	1.23-1.74	1.16	0.95-1.42	1.41	1.06-1.87
Obesity class III (≥ 40 kg/m²)	1.32	1.14-1.54	1.45	1.20-1.74	1.33	1.10-1.59	1.77	1.35-2.33

Degrees of obesity defined by the WHO cutoff points.
* Crude and adjusted multinomial logistic regression models for mechanical ventilation use (n=8075).
** Crude and adjusted logistic regression models for ICU admission (n=8414) and mortality (n=6565).
# Adjusted for sex, age in years, diabetes mellitus, cardiovascular disease, pulmonary disease, asthma, kidney disease, hematologic disease, neurological disease, liver disease, and immunosuppression.

For peer review only

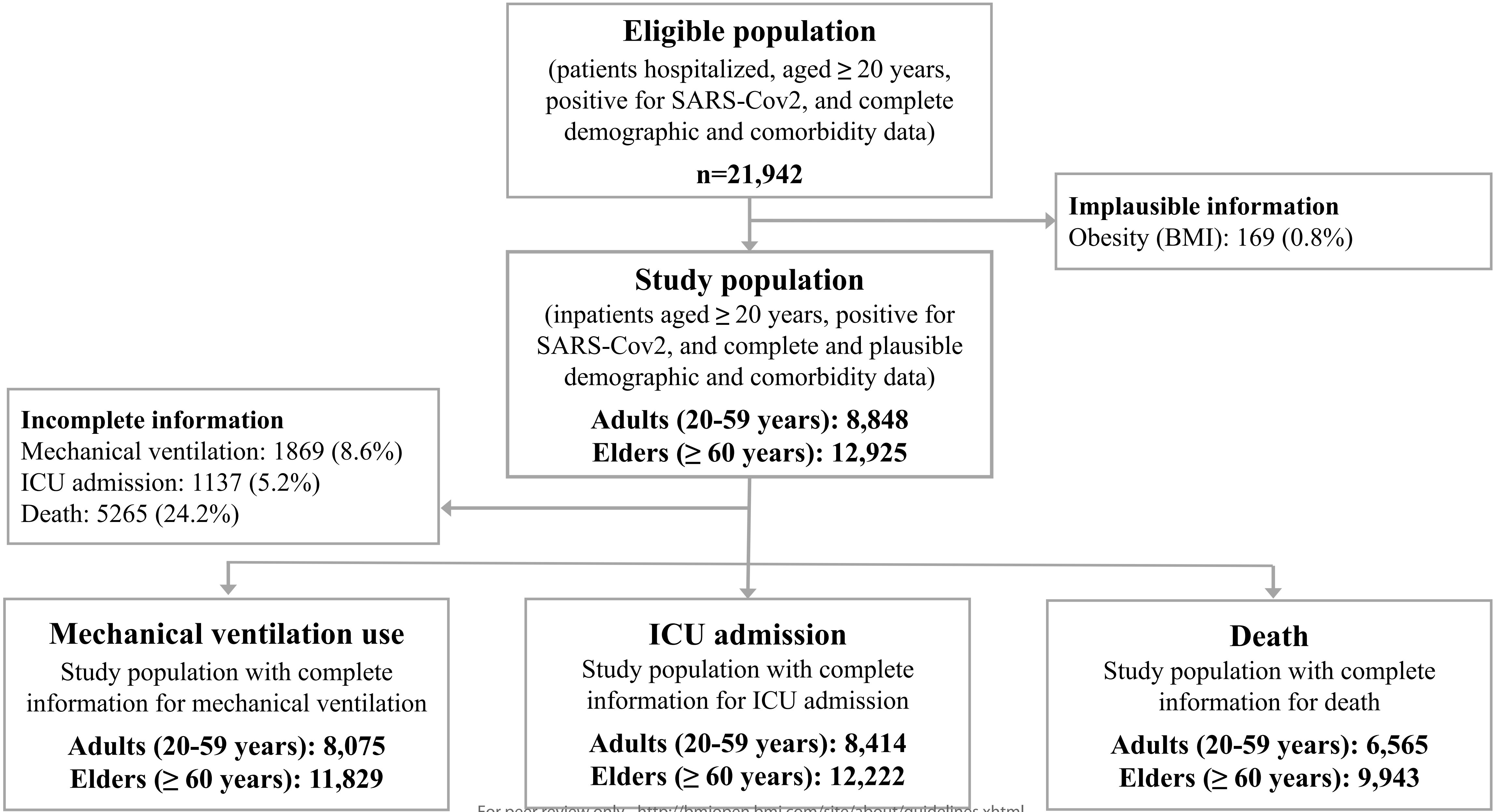

For peer review only - http://bmjopen.bmj.com/site/about/guidelines.xhtml

The RECORD statement – checklist of items, extended from the STROBE statement, that should be reported in observational studies using routinely collected health data.

	Item No.	STROBE items	Location in manuscript where items are reported	RECORD items	Location in manuscript where items are reported
**Title and abstract**					
	1	(a) Indicate the study's design with a commonly used term in the title or the abstract (b) Provide in the abstract an informative and balanced summary of what was done and what was found		RECORD 1.1: The type of data used should be specified in the title or abstract. When possible, the name of the databases used should be included.<br><br>RECORD 1.2: If applicable, the geographic region and timeframe within which the study took place should be reported in the title or abstract.<br><br>RECORD 1.3: If linkage between databases was conducted for the study, this should be clearly stated in the title or abstract.	Page 2<br>Lines 23-24<br><br>Page 2<br>Line 25<br><br>N/A
**Introduction**					
Background rationale	2	Explain the scientific background and rationale for the investigation being reported			Pages 4-5<br>Lines 79-82
Objectives	3	State specific objectives, including any prespecified hypotheses			Pages 4-5<br>Lines 82-85
**Methods**					
Study Design	4	Present key elements of study design early in the paper			Page 5<br>Line 88
Setting	5	Describe the setting, locations, and relevant dates, including periods of recruitment, exposure, follow-up, and data collection			Page 5<br>Lines 90-91, 95

For peer review only

Participants	6	*(a) Cohort study* - Give the eligibility criteria, and the sources and methods of selection of participants. Describe methods of follow-up<br>*Case-control study* - Give the eligibility criteria, and the sources and methods of case ascertainment and control selection. Give the rationale for the choice of cases and controls<br>*Cross-sectional study* - Give the eligibility criteria, and the sources and methods of selection of participants<br><br>*(b) Cohort study* - For matched studies, give matching criteria and number of exposed and unexposed<br>*Case-control study* - For matched studies, give matching criteria and the number of controls per case		RECORD 6.1: The methods of study population selection (such as codes or algorithms used to identify subjects) should be listed in detail. If this is not possible, an explanation should be provided.<br><br>RECORD 6.2: Any validation studies of the codes or algorithms used to select the population should be referenced. If validation was conducted for this study and not published elsewhere, detailed methods and results should be provided.<br><br>RECORD 6.3: If the study involved linkage of databases, consider use of a flow diagram or other graphical display to demonstrate the data linkage process, including the number of individuals with linked data at each stage.	Page 5<br>Lines 93-97
Variables	7	Clearly define all outcomes, exposures, predictors, potential confounders, and effect modifiers. Give diagnostic criteria, if applicable.		RECORD 7.1: A complete list of codes and algorithms used to classify exposures, outcomes, confounders, and effect modifiers should be provided. If these cannot be reported, an explanation should be provided.	Pages 5-6<br>Lines 102-128
Data sources/ measurement	8	For each variable of interest, give sources of data and details of methods of assessment (measurement).<br>Describe comparability of assessment methods if there is more than one group			Pages 5-6<br>Lines 102-128

Bias	9	Describe any efforts to address potential sources of bias			Pages 10-11<br>Lines 228-254
Study size	10	Explain how the study size was arrived at			Page 5<br>Lines 88-97
Quantitative variables	11	Explain how quantitative variables were handled in the analyses. If applicable, describe which groupings were chosen, and why			Pages 5-6<br>Lines 103-116
Statistical methods	12	(a) Describe all statistical methods, including those used to control for confounding<br>(b) Describe any methods used to examine subgroups and interactions<br>(c) Explain how missing data were addressed<br>(d) *Cohort study* - If applicable, explain how loss to follow-up was addressed<br>*Case-control study* - If applicable, explain how matching of cases and controls was addressed<br>*Cross-sectional study* - If applicable, describe analytical methods taking account of sampling strategy<br>(e) Describe any sensitivity analyses			Pages 6-7<br>Lines 130-142
Data access and cleaning methods		..		RECORD 12.1: Authors should describe the extent to which the investigators had access to the database population used to create the study population.	Page 5<br>Lines 90-93

For peer review only

				RECORD 12.2: Authors should provide information on the data cleaning methods used in the study.	
Linkage		..		RECORD 12.3: State whether the study included person-level, institutional-level, or other data linkage across two or more databases. The methods of linkage and methods of linkage quality evaluation should be provided.	N/A
**Results**					
Participants	13	(a) Report the numbers of individuals at each stage of the study (*e.g.*, numbers potentially eligible, examined for eligibility, confirmed eligible, included in the study, completing follow-up, and analysed)<br>(b) Give reasons for non-participation at each stage.<br>(c) Consider use of a flow diagram		RECORD 13.1: Describe in detail the selection of the persons included in the study (*i.e.,* study population selection) including filtering based on data quality, data availability and linkage. The selection of included persons can be described in the text and/or by means of the study flow diagram.	Page 7<br>Lines 147-154
Descriptive data	14	(a) Give characteristics of study participants (*e.g.*, demographic, clinical, social) and information on exposures and potential confounders<br>(b) Indicate the number of participants with missing data for each variable of interest<br>(c) *Cohort study* - summarise follow-up time (*e.g.*, average and total amount)			Page 7<br>Lines 152-156
Outcome data	15	*Cohort study* - Report numbers of outcome events or summary measures over time<br>*Case-control study* - Report numbers in each exposure			Pages 7-8<br>Lines 157-159

For peer review only

		category, or summary measures of exposure<br>*Cross-sectional study* - Report numbers of outcome events or summary measures			
Main results	16	(a) Give unadjusted estimates and, if applicable, confounder-adjusted estimates and their precision (e.g., 95% confidence interval). Make clear which confounders were adjusted for and why they were included<br>(b) Report category boundaries when continuous variables were categorized<br>(c) If relevant, consider translating estimates of relative risk into absolute risk for a meaningful time period			Pages 8<br>Lines 160-170
Other analyses	17	Report other analyses done—e.g., analyses of subgroups and interactions, and sensitivity analyses			Page 8<br>Lines 171-173
**Discussion**					
Key results	18	Summarise key results with reference to study objectives			Page 8<br>Lines 177-181
Limitations	19	Discuss limitations of the study, taking into account sources of potential bias or imprecision. Discuss both direction and magnitude of any potential bias		RECORD 19.1: Discuss the implications of using data that were not created or collected to answer the specific research question(s). Include discussion of misclassification bias, unmeasured confounding, missing data, and changing eligibility over time, as they pertain to the study being reported.	Page 11<br>Lines 238-254
Interpretation	20	Give a cautious overall interpretation of results considering objectives,			Pages 9-10<br>Lines 182-227

For peer review only

		limitations, multiplicity of analyses, results from similar studies, and other relevant evidence			
Generalisability	21	Discuss the generalisability (external validity) of the study results			Page 11 Lines 253-254
**Other Information**					
Funding	22	Give the source of funding and the role of the funders for the present study and, if applicable, for the original study on which the present article is based			Page 12 Lines 273-279
Accessibility of protocol, raw data, and programming code		..		RECORD 22.1: Authors should provide information on how to access any supplemental information such as the study protocol, raw data, or programming code.	Page 13 Lines 284-286

*Reference: Benchimol EI, Smeeth L, Guttmann A, Harron K, Moher D, Petersen I, Sørensen HT, von Elm E, Langan SM, the RECORD Working Committee. The REporting of studies Conducted using Observational Routinely-collected health Data (RECORD) Statement. *PLoS Medicine* 2015; in press.

*Checklist is protected under Creative Commons Attribution (CC BY) license.

BMJ Open

Combined association of obesity and other cardiometabolic diseases with severe COVID-19 outcomes: a nationwide cross-sectional study of 21,773 Brazilian adult and elderly inpatients

Journal:	*BMJ Open*
Manuscript ID	bmjopen-2021-050739.R1
Article Type:	Original research
Date Submitted by the Author:	15-May-2021
Complete List of Authors:	Silva, Natanael; Oswaldo Cruz Foundation, Centre for Data and Knowledge Integration for Health<br>Silva, Rita de Cássia; Federal University of Bahia, School of Nutrition; Oswaldo Cruz Foundation, Centre for Data and Knowledge Integration for Health<br>Ferreira, Andrêa; Federal University of Bahia, Institute of Collective Health; Oswaldo Cruz Foundation, Centre for Data and Knowledge Integration for Health<br>Teixeira, Camila; Federal University of Bahia, Institute of Collective Health; Oswaldo Cruz Foundation, Centre for Data and Knowledge Integration for Health<br>Rocha, Aline; Federal University of Bahia, School of Nutrition; Oswaldo Cruz Foundation, Centre for Data and Knowledge Integration for Health<br>Alves, Flávia Jôse; Federal University of Bahia, Institute of Collective Health; Oswaldo Cruz Foundation, Centre for Data and Knowledge Integration for Health<br>Falcão, Ila; Oswaldo Cruz Foundation, Centre for Data and Knowledge Integration for Health<br>Pinto, Elizabete; Federal University of Reconcavo da Bahia, Center for Health Sciences; Oswaldo Cruz Foundation, Centre for Data and Knowledge Integration for Health<br>Santos, Carlos Antônio; Oswaldo Cruz Foundation, Centre for Data and Knowledge Integration for Health; State University of Feira de Santana, Department of Exact Sciences<br>Fiaccone, Rosemeire; Federal University of Bahia, Institute of Mathematics and Statistics; Oswaldo Cruz Foundation, Centre for Data and Knowledge Integration for Health<br>Ichihara, Maria Yury; Oswaldo Cruz Foundation, Centre for Data and Knowledge Integration for Health<br>Paixão, Enny; London School of Hygiene and Tropical Medicine Faculty of Epidemiology and Population Health; Oswaldo Cruz Foundation, Centre for Data and Knowledge Integration for Health<br>Barreto, Mauricio; Oswaldo Cruz Foundation, Centre for Data and Knowledge Integration for Health; Federal University of Bahia, Institute of Collective Health
**Primary Subject Heading**:	Epidemiology

Secondary Subject Heading:	Infectious diseases, Nutrition and metabolism
Keywords:	COVID-19, INFECTIOUS DISEASES, INTENSIVE & CRITICAL CARE, DIABETES & ENDOCRINOLOGY, NUTRITION & DIETETICS, EPIDEMIOLOGY

SCHOLARONE™ Manuscripts

I, the Submitting Author has the right to grant and does grant on behalf of all authors of the Work (as defined in the below author licence), an exclusive licence and/or a non-exclusive licence for contributions from authors who are: i) UK Crown employees; ii) where BMJ has agreed a CC-BY licence shall apply, and/or iii) in accordance with the terms applicable for US Federal Government officers or employees acting as part of their official duties; on a worldwide, perpetual, irrevocable, royalty-free basis to BMJ Publishing Group Ltd ("BMJ") its licensees and where the relevant Journal is co-owned by BMJ to the co-owners of the Journal, to publish the Work in this journal and any other BMJ products and to exploit all rights, as set out in our licence.

The Submitting Author accepts and understands that any supply made under these terms is made by BMJ to the Submitting Author unless you are acting as an employee on behalf of your employer or a postgraduate student of an affiliated institution which is paying any applicable article publishing charge ("APC") for Open Access articles. Where the Submitting Author wishes to make the Work available on an Open Access basis (and intends to pay the relevant APC), the terms of reuse of such Open Access shall be governed by a Creative Commons licence – details of these licences and which Creative Commons licence will apply to this Work are set out in our licence referred to above.

Other than as permitted in any relevant BMJ Author's Self Archiving Policies, I confirm this Work has not been accepted for publication elsewhere, is not being considered for publication elsewhere and does not duplicate material already published. I confirm all authors consent to publication of this Work and authorise the granting of this licence.

For peer review only

Combined association of obesity and other cardiometabolic diseases with severe COVID-19 outcomes: a nationwide cross-sectional study of 21,773 Brazilian adult and elderly inpatients

Natanael J Silva, MSc[1,2]; Rita C Ribeiro-Silva, PhD[1,2,3]; Andrêa JF Ferreira, MSc[1,2,4]; Camila SS Teixeira, MSc[1,2,4]; Aline S Rocha, MSc[1,2,3]; Flávia Jôse O Alves, MSc[1,2,4]; Ila R Falcão, PhD[2]; Elizabete J Pinto, PhD[2,5]; Carlos Antônio ST Santos, PhD[1,2,6]; Rosemeire L Fiaccone, PhD[1,2,7]; Maria Yury T Ichihara, MD PhD[1,2]; Enny S Paixão, PhD[1,2,8]; Mauricio L Barreto, MD PhD[1,2,4]

[1] Rede CoVida, Salvador, BA, Brazil

[2] Centre for Data and Knowledge Integration for Health, Oswaldo Cruz Foundation, Salvador, BA, Brazil

[3] School of Nutrition, Federal University of Bahia, Salvador, BA, Brazil

[4] Institute of Collective Health, Federal University of Bahia, Salvador, BA, Brazil

[5] Center for Health Sciences, Federal University of Recôncavo da Bahia, Santo Antônio de Jesus, BA, Brazil

[6] Department of Exact Sciences, State University of Feira de Santana, Feira de Santana, BA, Brazil

[7] Institute of Mathematics and Statistics, Federal University of Bahia, Salvador, BA, Brazil

[8] Epidemiology and Population Health, London School of Hygiene and Tropical Medicine, London, UK

Corresponding author: Natanael J Silva. Centre for Data and Knowledge Integration for Health, Oswaldo Cruz Foundation. Edf. Tecnocentro, Sl 315. Rua Mundo, 121. Trobogy, Salvador, BA, 41745-715, Brazil. Phone number: +55 79 99839-3991. Email: <silva_natanael@hotmail.com>.

Keywords: Obesity, COVID-19, SARS-CoV-2, Hospitalization, Mortality.

Word count: 3,152

For peer review only

Abstract

Objectives: To investigate the combined association of obesity, diabetes mellitus (DM), and cardiovascular disease (CVD) with severe COVID-19 outcomes in adult and elderly inpatients.

Design: Cross-sectional study based on registry data from Brazil's influenza surveillance system.

Setting: Public and private hospitals across Brazil.

Participants: Eligible population included 21,942 inpatients aged ≥20 years with positive RT-PCR test for SARS-CoV-2 until Jun 9th, 2020.

Main outcome measures: Severe COVID-19 outcomes were non-invasive and invasive mechanical ventilation use, ICU admission, and death. Multivariate analyses were conducted separately for adults (20-59 years) and elders (≥60 years) to test the combined association of obesity (without and with DM and/or CVD) and degrees of obesity with each outcome.

Results: A sample of 8,848 adults and 12,925 elders were included. Among adults, obesity with DM and/or CVD showed higher prevalence of invasive (PR 3.76, 95%CI 2.82-5.01) and non-invasive mechanical ventilation use (2.06, 1.58-2.69), ICU admission (1.60, 1.40-1.83), and death (1.79, 1.45-2.21) compared with the group without obesity, DM, and CVD. In elders, obesity alone (without DM and CVD) had the highest prevalence of ICU admission (1.40, 1.07-1.82) and death (1.67, 1.00-2.80). In both age groups, obesity alone and combined with DM and/or CVD showed higher prevalence in all outcomes than DM and/or CVD. A dose-response association was observed between obesity and death in adults: class I 1.32 (1.05-1.66), class II 1.41 (1.06-1.87), and class III 1.77 (1.35-2.33).

Conclusions: The combined association of obesity, diabetes, and/or CVD with severe COVID-19 outcomes may be stronger in adults than in elders. Obesity alone and combined with DM and/or CVD had more impact on the risk of COVID-19 severity than DM and/or CVD in both age groups. The study also supports an independent relationship of obesity with severe outcomes, including a dose-response association between degrees of obesity and death in adults.

For peer review only

Article summary

Strengths and limitations of this study:

- This is the first study that describes the independent and combined relationship of obesity with COVID-19 severity in Brazil, one of the biggest epicenters of the pandemic worldwide.
- The study was based on registry data of a large nationwide sample of patients admitted, due to severe SARS-CoV-2 infection, to public and private hospitals across the country.
- The large sample size and data availability allowed us to analyze the combined association of obesity, diabetes and cardiovascular disease with severe COVID-19 outcomes, separately by age groups and controlled by important confounding variables, e.g. underlying comorbidities.
- The cross-sectional study design does not allow causal inference, and generalization of results must be cautious since only hospitalized cases of severe COVID-19 were included.
- As the study used routinely collected data, which has not been designed primarily for research purposes, it may bring well-known limitations related to missing, underestimation, and potential misclassification.

For peer review only

Introduction

The coronavirus disease 2019 (COVID-19) pandemic, caused by the severe acute respiratory syndrome coronavirus 2 (SARS-CoV-2), as of 13 May 2021, has already reached more than 160 million infected people and more than 3.3 million deaths in all continents.[1] Individuals with advanced age and chronic diseases, including cardiometabolic diseases, are considered groups at major risk for complications and severe illness from COVID-19.[2,3] Obesity has been shown as an independent risk factor for COVID-19 disease.[4-6] High body mass index (BMI) has been mentioned as a significant risk factor for COVID-19, according to early clinical reports from China,[7] Italy,[8] France,[9] Mexico,[10] and United States of America.[11] Several studies have demonstrated that obesity is leading to considerably worse COVID-19 outcomes, especially greater risk of hospital and intensive care unit (ICU) admission, invasive mechanical ventilation, and death.[11-14]

The COVID-19 pandemic is rapidly spreading worldwide, especially in the Americas, where obesity is already a prevalent and important public health problem.[15-16] Brazil is currently one of the biggest epicenters of the COVID-19 pandemic worldwide, with more than 15.2 million cases and 425 thousand deaths until May 13, 2021.[1] In 2018, the prevalence of adult overweight and obesity in Brazil was estimated at 55.7% and 19.8%, respectively.[17] This obesogenic profile of the Brazilian population contributes, among other factors, to the high prevalence of obesity-related diseases such as type 2 diabetes mellitus (DM) and cardiovascular diseases (CVD), in the country.[18] The fact that individuals with obesity also have more comorbidity diseases, which are either risk factors for COVID-19 severity and death, makes obesity particularly ominous in COVID-19 disease.[10-13]

Several characteristics that can influence the clinical evolution of individuals infected with COVID-19, such as obesity, have been independently documented.[5-6,19] However, evidence is yet unclear on the combined effect that obesity and obesity-related comorbidities play in COVID-19 severity, especially, in different age groups. We aimed in this study to investigate the combined association of obesity, diabetes, and cardiovascular disease with mechanical ventilation use, ICU admission, and

For peer review only

death in a large sample of adult and elderly patients hospitalized with COVID-19 in Brazil. We also explored the independent association between degrees of obesity and the mentioned outcomes.

Methods

Study Design and Population

This is a cross-sectional study based on registry data from SIVEP-Gripe (Sistema de Informação de Vigilância Epidemiológica da Gripe), an influenza surveillance system of Brazil's Ministry of Health. The study used the publicly available dataset of SIVEP-Gripe, which includes de-identified data on cases of severe acute respiratory syndrome across public and private hospitals in Brazil.[20] These data were obtained through the Rede CoVida's integrated data platform that has been built with official, open, and authorized data for the production of knowledge about the COVID-19 pandemic.

Our study population was composed of patients aged 20 years or older, hospitalized for severe acute respiratory syndrome, with positive RT-PCR test for SARS-CoV-2, and final diagnosis for COVID-19 until Jun 9th, 2020. Only cases with complete data on demographic characteristics and comorbidities and plausible BMI values were included in the study.

Exposure Variable

Obesity was defined as BMI equal to or greater than 30 kg/m^2, according to the cutoff points proposed by the World Health Organization[21] and the Pan American Health Organization[22] for adults and elders, respectively. BMI was calculated by health professionals in the hospital from directly measured or patient self-reported height and weight. Guidelines for the collection and analysis of anthropometric data in health services have been previously standardized by the Ministry of Health.[23] BMI values <12 or >70 kg/m^2 were considered implausible and excluded.[24] Information on the existence of diabetes and any chronic cardiovascular disease was obtained from dichotomous questions (yes/no), which were answered based on patient or family's report or medical diagnosis.

We created a polytomous four-category variable to evaluate the separate and combined exposure of obesity, diabetes and cardiovascular disease: none/reference (no existence of obesity, diabetes and

For peer review only

cardiovascular disease), OB (only existence of obesity), OB + DM and/or CVD (existence of obesity with diabetes and/or cardiovascular disease), and DM and/or CVD (existence of diabetes and/or cardiovascular disease). We also analyzed obesity in adults according to the following degrees of severity based on WHO reference[21]: no obesity (<30 kg/m^2), obesity class I (≥ 30-34.9 kg/m^2), obesity class II (≥ 35-39.9 kg/m^2), and obesity class III (≥ 40 kg/m^2). Due to the unavailability of BMI cutoff points to classify the degree of obesity in elders, this analysis was only performed for adults.

Outcome Variables

The severe COVID-19 outcomes were mechanical ventilation use, ICU admission, and death. Information on the use of mechanical ventilation by the patient was obtained and analyzed as a polytomous three-category variable (no use/ use of non-invasive ventilation/ use of invasive ventilation). ICU admission was obtained and analyzed as a dichotomous variable (no/ yes). Death was analyzed as a dichotomous variable based on the patient's endpoint outcome (cure/ death).

Covariates

Demographic and comorbidity information were selected as descriptive and confounding variables.[2] Age in years was calculated from birth and notification dates. Sex was obtained as a dichotomous variable (female/ male). The preexistence of each comorbidity was also obtained as a dichotomous variable (no/ yes): chronic pulmonary disease, asthma, chronic kidney disease, chronic hematologic disease, neurological disease, chronic liver disease, and immunodeficiency/ immunosuppression.

Statistical Analysis

All analyses were subdivided into adults (≥ 20 and < 60 years) and elders (≥ 60 years). For descriptive analyses, absolute and relative frequencies were calculated for the demographic and comorbidity variables according to the main exposure variable. Multinomial logistic regression models were conducted to test the association of obesity (without and with diabetes and/or CVD) with non-invasive and invasive mechanical ventilation use. To test the association of this exposure variable with ICU admission and death, simple logistic regression models were performed. Same models were analyzed

For peer review only

considering the degree of obesity as the main exposure variable for adults. Crude and adjusted estimates were interpreted based on the prevalence ratio (PR) and 95% confidence intervals (95%CI). These estimates were obtained from logistic models using delta method, function 'prLogisticDelta', which is implemented in R and available in the package 'prLogistic'. Adjusted models included the following list of confounding variables: sex, age (years), and the preexistence of chronic pulmonary disease, asthma, kidney disease, hematologic disease, neurological disease, liver disease, and immunodeficiency/ immunosuppression. The models that tested the degrees of obesity were also adjusted for DM and CVD. All analyses were performed using Stata version 15.1 (Stata Corporation, College Station, USA) and R version 3.6.1 (R Foundation for Statistical Computing, Austria).

Patient and Public Involvement

As the study exclusively used publicly available de-identified data, it was not possible to involve patients or the public in the design, or conduct, or reporting, or dissemination plans of our research.

Results

During the study period, 21,942 individuals registered in the SIVEP-Gripe were ≥ 20 years old, hospitalized, tested positive for SARS-CoV-2, and had complete demographic and comorbidity information (**Figure 1**). Of these, 169 (0.8%) were excluded due to implausible values of BMI. Of the 21,773 individuals included in the study, 8,848 (40.3%) were adults aged between 20-59 years, and 12,925 (59.6%) were elders aged 60 years or older. Since some patients were still hospitalized on the study endpoint date, information for some outcomes were incomplete. The study samples included in the analysis of each outcome were 8,075 adults and 11,829 elders for mechanical ventilation, 8,414 adults and 12,222 for ICU admission, and 6,565 adults and 9,943 elders for death. Based on demographic and clinical characteristics, the analytical samples in each outcome were very similar to the overall study population and the excluded samples (**Supplementary Table 1**).

The prevalence of obesity was 9.7% in adults and 3.5% in elders. The frequency of obesity without and with DM and/or CVD was respectively 4.6% and 5.1% in adults and 0.7% and 2.8% in elders.

For peer review only

Non-invasive and invasive mechanical ventilation were respectively required by 45.0% and 21.2% of adults and 47.0% and 30.0% of elders. ICU admission was needed by 35.4% of adults and 43.6% of elders. Death occurred in 31.1% of adult and 63.0% of elderly patients (**Table 1-2**).

In the adjusted analyses for adults, obesity alone (without DM and CVD) was associated with an increased prevalence of invasive (PR 2.69, 95%CI 1.98-3.65) and non-invasive mechanical ventilation need (PR 2.13, 95%CI 1.64-2.78), ICU admission (PR 1.31, 95%CI 1.13-1.53), and death (PR 1.33, 95%CI 1.05-1.69) when compared with the group without obesity, DM, and CVD. Obesity with DM and/or CVD was associated with an even higher prevalence of invasive mechanical ventilation (PR 3.76, 95%CI 2.82-5.01) and non-invasive ventilation use (PR 2.06, 95%CI 1.58-2.69), ICU admission (PR 1.60, 95%CI 1.40-1.83), and death in adults (PR 1.79, 95%CI 1.45-2.21). The subgroup of adults with DM and/or CVD showed in general the lowest prevalence ratios for all analyzed outcomes than the subgroups with the presence of obesity alone or combined (**Table 3**).

Among elders, obesity without DM and CVD increased independently the prevalence of ICU admission by 40% (95%CI 1.07-1.82) and death by 67% (1.00-2.80). To a lesser extent, obesity with DM and/or CVD was also associated with an increased prevalence of invasive mechanical ventilation need (PR 1.66, 95%CI 1.22-2.27), ICU admission (PR 1.37, 95%CI 1.19-1.59), and death (PR 1.39, 95%CI 1.07-1.80). Elders with DM and/or CVD had the lowest prevalence ratios for the analyzed outcomes than the subgroups of elders with obesity alone or combined (**Table 3**).

In the analyses by the degree of obesity, we did not observe much difference in the prevalence of adverse outcomes, except for the prevalence of death that increased with the severity of obesity: Class I 1.32 (95%CI 1.05-1.66), Class II 1.41 (1.06-1.87), and Class III 1.77 (1.35-2.33) (**Table 4**).

Discussion

This is the first study that describe the relationship of obesity and COVID-19 in Brazil, based on a large nationwide sample of adults and elders tested positive for SARS-CoV-2 and admitted to public and private hospitals. Our results highlights that obesity with DM and/or CVD was associated with

For peer review only

higher rates of invasive mechanical ventilation use, ICU admission, and death in adults, while obesity alone (without DM and CVD) was associated with higher rates of ICU admission and death among elders. In both age groups, obesity alone and obesity combined with DM and/or CVD had more impact on the risk of all severe COVID-19 outcomes than the subgroup with DM and/or CVD. The study also supports the independent association of obesity with the analyzed outcomes and a dose-response association between degrees of obesity and death in adults.

Some mechanisms related to the role of obesity and related diseases in worsening the clinical condition of patients affected by SARS-CoV-2 have been pointed out: i) greater body weight causes less elasticity of the chest wall and less total compliance of the respiratory system, leading to a restriction of the ventilation and the excursion of the diaphragm, making difficult the airway management in patients with obesity;[25] ii) obesity is associated with sleep apnea syndrome and chronic obstructive pulmonary disease, which lead to surfactant dysfunction and impede the proper functioning of the airways;[26] iii) obesity is a metabolic and inflammatory disease, which is associated with the development or worsening of other chronic and endocrine comorbidities (e.g. type 2 diabetes, hypertension, dyslipidemia and CVD) that can modify innate and adaptive immune responses, making the immune system more vulnerable to infections and less responsive to antivirals and antimicrobial drugs;[16] iv) glycemic decompensation, common in patients with obesity, is associated with impaired ventilation function.[26]

It is important to note that the COVID-19 pandemic imposes a double burden of disease, especially among the elderly individuals, since the prevalence of diabetes, hypertension, cardiovascular diseases, and other comorbidities associated with COVID-19 severity increases with age.[3,27] However, our study suggests that obesity combined with diabetes and/or cardiovascular disease may offer higher risk of COVID-19 severity for adults although the overall prevalence of diseases and rates of ICU admission and mortality were higher in elders. Obesity alone seemed to provide higher risk of severe outcomes, especially death, in elders.

For peer review only

Few studies to-date have explored the combined and additional effect of obesity on COVID-19 severity.[13,28] A study investigated the patterns of multimorbidity among fatal cases of COVID-19 in Colombia.[28] Similar to our study, the authors found that obesity alone or with other diseases was associated with a higher risk of COVID-19 fatality among young people. Furthermore, a population-based study in Mexico observed that the addition of obesity to any number of comorbidities significantly increased the risk of COVID-19 lethality.[13] Using a causally ordered mediation analysis, this study also found that 49.5% of the effect of diabetes on COVID-19 lethality was mediated by obesity, particularly in early-onset cases < 40 years of age.

Other studies also suggest that obesity is independently associated with severe outcomes of COVID-19, regardless of age and other associated comorbidities.[11-14] A large study in Mexico[13] showed that patients with obesity had higher rates of ICU admission and were more likely to be intubated in relation to patients without obesity. This study also found a five-fold increased risk of mortality due to COVID-19 in patients with obesity.[13] In a hospital-based study in France, it was observed that BMI > 35 kg/m^2 was associated with the need for invasive mechanical ventilation.[14]

Few studies to-date have similarly found a dose-response association between degrees of obesity and COVID-19 death.[29] Based on care records of 17,278,392 UK adults, the study showed that the risk of COVID-19 death increases independently with the degree of obesity: 30-34.9 kg/m^2 (HR 1.05), 35-39.9 kg/m^2 (1.40), and $\geq$40 kg/m^2 (2.66).[29] Other studies have evidenced the association of obesity with COVID-19 complications and death among adults.[12,30] A hospital-based study in New York City showed that morbid obesity (BMI ≥ 40 kg/m^2) is strongly and independently associated with death in hospitalized patients younger than 50 years.[30] Another study in New York City found a similar dose-response relationship between degrees of obesity and acute and critical care.[12] Patients less than 60 years old with BMI between 30 and 34.9 kg/m^2 (obesity class I) were 2.0 and 1.8 times more likely to be respectively admitted for acute care (general hospital admission) and critical care (ICU admission or invasive ventilator) compared to individuals with BMI < 30 kg/m^2. Patients of the same

For peer review only

age group with BMI ≥ 35 kg/m^2 (obesity class II and III) showed 2.2 and 3.6 more chances of being hospitalized for acute and critical care, respectively.[12]

Strengths and limitations

One of the greatest strengths of the study was the use of SIVEP-Gripe dataset. Because severe acute respiratory syndrome is a condition of compulsory notification in both public and private hospitals,[31] we have a nationwide representative sample of patients hospitalized for severe COVID-19 in Brazil. In addition, the large sample sizes allowed us to analyze adults and elders separately, as well as the degrees of obesity which dose-response association with death was evidenced. The availability of important confounding variables (sex, age, and preexisting comorbidities) to control the estimated associations, as well as hospital outcomes and mortality of COVID-19, was another differential of the study. Only patients with positive RT-PCR test for SARS-CoV-2 and final diagnosis for COVID-19 were included which gives greater precision on the studied population. The availability and use of data from health surveillance systems may be a lesson from Brazil that other countries can learn for obtaining routine and timely data to guide health systems and research in preparing and responding to pandemics before and during their course.

The study also has some limitations that must be considered. Because this is a cross-sectional study, a causal association cannot be inferred. As we used routinely collected data, which has not been designed primarily for research purposes, it may bring well-known limitations related to missing, underestimation, and potential misclassification. Obesity prevalence may have been underestimated due to the completeness of obesity and BMI data. Previous studies using SIVEP-Gripe data have also found a low prevalence of obesity in this population.[32,33] Better routine collection of height and weight data is still needed in clinical practice. Also, we believe that health professionals have adopted more the one method to collect weight and height information for BMI calculation, such as the patient's self-report and direct measure. Therefore, in addition to BMI which implausible values were checked and excluded, the classification of obesity was also confirmed from a dichotomous variable on the presence of obesity (no/yes). Although it is known that BMI does not distinguish between fat and

For peer review only

lean body mass, and thus may lead to misclassification bias, BMI has been shown as a strong predictor of excess body fat and has been widely used in epidemiological studies.[15] Information for some outcomes were incomplete because some patients were still hospitalized on the study endpoint date. However, that did not represent a potential selection bias to our study. The analytical samples in each outcome had similar demographic and clinical characteristics than the overall study population and the excluded samples (Supplementary Table 1). Data on ethnicity/race was very incomplete, and thus was not included in the analysis. Additional studies are needed to further explore the relationship between socioeconomic characteristics and obesity in severe disease. Finally, the generalization of results must be cautious since the study included only hospitalized cases of COVID-19.

Conclusions

The combined association of obesity, diabetes, and/or cardiovascular disease with severe COVID-19 outcomes, especially ICU admission and death, may be stronger in adult than in elderly inpatients. In both age groups, obesity alone and obesity combined with DM and/or CVD had more impact on the risk of all severe COVID-19 outcomes than the subgroup with DM and/or CVD. The study also supports an independent relationship of obesity with the severe outcomes, including a dose-response association between degrees of obesity and death in adults. These findings suggest important implications for the clinical care of patients with obesity and severe COVID-19, such as the increased need of critical care and higher risk of death among these patients. Our study also supports the inclusion of people with obesity, independently of other preexisting comorbidities and age, in the high-risk and vaccine priority groups for protection from SARS-CoV-2 infection.

Acknowledgments

The authors thank the members of Rede CoVida's Epidemiology & Information Group for the work of identifying and collecting data related to COVID-19.

Author Contributions

For peer review only

NJS, RCRS, and RLF designed the study and analysis strategy. NJS, CASTS, and MYTI obtained, documented, and described the data. AJFF, CSST, ASR, FJOA, and IRF carried out the literature search. NJS and EJP performed the data analysis. NJS, RCRS, AJFF, CSST, ASR, FJOA, IRF, ESP and MLB contributed to data interpretation. NJS, AJFF, CSST, ASR, FJOA, and IRF drafted the manuscript. RCRS, ESP, MYTI, and MLB critically revised the manuscript. All authors read and approved the final manuscript.

Funding

All authors are affiliated to the Centre for Data and Knowledge Integration for Health (CIDACS) that is funded and supported by MCTI/ CNPq/ MS/ SCTIE/ Decit/ Bill & Melinda Gates Foundation's GCE Brazil (OPP1142172), Wellcome Trust (202912/Z/16/Z), the Brazilian Health Surveillance Secretariat, Ministry of Health, Bahia State, Research Support Foundation of the State of Bahia (FAPESB), the Research and Project Funding Agency (FINEP), and the Secretariat of Science and Technology of the State of Bahia (SECTI). Dr Paixão is a fellow supported by the Wellcome Trust (213589/Z/18/Z).

Competing Interests

None declared.

Patient Consent for Publication

Not required.

Data Availability Statement

Data is freely available without restriction at https://opendatasus.saude.gov.br/dataset/bd-srag-2020. Code book and analytic code will be made available upon request from the corresponding author.

Ethics Statement

The study was conducted according to the guidelines laid down in the Declaration of Helsinki. As the study exclusively used publicly available de-identified data, ethics approval by a research ethics committee and informed consent are waived per Resolution n. 466/2012 of the National Health Council of Brazil's Commission of Ethics in Research.

For peer review only

References

1. World Health Organization. WHO Coronavirus Disease (COVID-19) Dashboard. https://covid19.who.int/. Accessed May 13, 2020.
2. Centers for Disease Control and Prevention. Coronavirus Disease 2019 (COVID-19). People Who Are at Increased Risk for Severe Illness. https://www.cdc.gov/coronavirus/2019-ncov/need-extra-precautions/people-at-increased-risk.html. Accessed July 21, 2020.
3. Liu H, Chen S, Liu M, Nie H, Lu H. Comorbid Chronic Diseases are Strongly Correlated with Disease Severity among COVID-19 Patients: A Systematic Review and Meta-Analysis. *Aging Dis*. 2020;11(3):668-678. doi:10.14336/ad.2020.0502.
4. Alberca RW, Oliveira LM, Branco ACCC, Pereira NZ, Sato MN. Obesity as a risk factor for COVID-19: an overview [published online June 15, 2020]. *Crit Rev Food Sci Nutr*. doi:10.1080/10408398.2020.1775546.
5. Sattar N, McInnes IB, McMurray JJV. Obesity Is a Risk Factor for Severe COVID-19 Infection: Multiple Potential Mechanisms. *Circulation*. 2020;142(1):4-6. doi:10.1161/circulationaha.120.047659.
6. Caussy C, Wallet F, Laville M, Disse E. Obesity is Associated with Severe Forms of COVID-19. *Obesity (Silver Spring)*. 2020;28(7):1175. doi:10.1002/oby.22842.
7. Li Q, Guan X, Wu P, et al. Early Transmission Dynamics in Wuhan, China, of Novel Coronavirus-Infected Pneumonia. *N Engl J Med*. 2020;382(13):1199-1207. doi:10.1056/nejmoa2001316.
8. Grasselli G, Zangrillo A, Zanella A, et al. Baseline Characteristics and Outcomes of 1591 Patients Infected with SARS-CoV-2 Admitted to ICUs of the Lombardy Region, Italy. *JAMA*. 2020;323(16):1574-1581. doi:10.1001/jama.2020.5394.
9. Caussy C, Pattou F, Wallet F, et al. Prevalence of obesity among adult inpatients with COVID-19 in France. *Lancet Diabetes Endocrinol*. 2020;8(7):562-564. doi:10.1016/s2213-8587(20)30160-1.
10. Hernández-Garduño E. Obesity is the comorbidity more strongly associated for Covid-19 in Mexico. A case-control study. *Obes Res Clin Pract*. 2020;14(4):375-379. doi:10.1016/j.orcp.2020.06.001.
11. Hajifathalian K, Kumar S, Newberry C, et al. Obesity is associated with worse outcomes in COVID-19: Analysis of Early Data From New York City. *Obesity (Silver Spring)*. 2020;28(9):1606-1612. doi:10.1002/oby.22923.
12. Lighter J, Phillips M, Hochman S, et al. Obesity in patients younger than 60 years is a risk factor for Covid-19 hospital admission. *Clin Infect Dis*. 2020;71(15):896-897. doi:10.1093/cid/ciaa415.
13. Bello-Chavolla OY, Bahena-López JP, Antonio-Villa NE, et al. Predicting Mortality Due to SARS-CoV-2: A Mechanistic Score Relating Obesity and Diabetes to COVID-19 Outcomes in Mexico. *J Clin Endocrinol Metab*. 2020;105(8):dgaa346. doi:10.1210/clinem/dgaa346.
14. Simonnet A, Chetboun M, Poissy J, et al. High Prevalence of Obesity in Severe Acute Respiratory Syndrome Coronavirus-2 (SARS-CoV-2) Requiring Invasive Mechanical Ventilation. *Obesity (Silver Spring)*. 2020;28(7):1195-1199. doi:10.1002/oby.22831.
15. NCD Risk Factor Collaboration (NCD-RisC). Worldwide trends in body-mass index, underweight, overweight, and obesity from 1975 to 2016: a pooled analysis of 2416 population-based measurement studies in 128.9 million children, adolescents, and adults. *Lancet*. 2017;390(10113):2627-2642. doi:10.1016/s0140-6736(17)32129-3.
16. GBD 2015 Obesity Collaborators. Health Effects of Overweight and Obesity in 195 Countries over 25 Years. *N Engl J Med*. 2017;377(1):13-27. doi:10.1056/nejmoa1614362.
17. Brasil. Ministério da Saúde. Vigitel Brasil 2018: vigilância de fatores de risco e proteção para doenças crônicas por inquérito telefônico. Brasília, DF: Ministério da Saúde; 2019. https://portalarquivos2.saude.gov.br/images/pdf/2019/julho/25/vigitel-brasil-2018.pdf. Accessed July 10, 2020.

18. GBD 2016 Brazil Collaborators. Burden of disease in Brazil, 1990-2016: a systematic subnational analysis for the Global Burden of Disease Study 2016. *Lancet*. 2018;392(10149):760-775. doi:10.1016/s0140-6736(18)31221-2.
19. Flint SW, Tahrani AA. COVID-19 and obesity-lack of clarity, guidance, and implications for care. *Lancet Diabetes Endocrinol*. 2020;8(6):474-475. doi:10.1016/s2213-8587(20)30156-x.
20. Brasil. Ministério da Saúde. Severe Acute Respiratory Syndrome dataset - including COVID-19 data. 2020. https://opendatasus.saude.gov.br/dataset/bd-srag-2020. Accessed July 11, 2020.
21. World Health Organization. Physical status: the use and interpretation of anthropometry. Geneva: WHO; 1995. https://apps.who.int/iris/handle/10665/37003. Accessed July 11, 2020.
22. Organización Panamericana de la Salud. División de Promoción y Protección de la Salud (HPP). Encuesta sobre Salud, Bienestar y Envejecimiento (SABE) en América Latina el Caribe: Informe Preliminar (Internet). In: XXXVI Reunión del Comité asesor de investigaciones em Salud; 9-11 jun 2001; Kingston: OPAS, 2002. http://envejecimiento.csic.es/documentos/documentos/paho-salud-01.pdf. Accessed July 11, 2020.
23. Brasil. Ministério da Saúde. Guidelines for the Collection and Analysis of Anthropometric Data in Health Services. Technical Standard for the Food and Nutrition Surveillance System. Brasília, DF: Ministério da Saúde; 2011.
24. Cheng FW, Gao X, Mitchell DC, et al. Body mass index and all-cause mortality among older adults. *Obesity (Silver Spring)*. 2016;24(10):2232-39. doi:10.1002/oby.21612.
25. Honce R, Schultz-Cherry S. Impact of Obesity on Influenza A Virus Pathogenesis, Immune Response, and Evolution. *Front Immunol*. 2019;10:1071. doi:10.3389/fimmu.2019.01071.
26. Puig-Domingo M, Marazuela M, Giustina A. COVID-19 and endocrine diseases. A statement from the European Society of Endocrinology. *Endocrine*. 2020;68(1):2-5. doi:10.1007/s12020-020-02294-5.
27. Docherty AB, Harrison EM, Green CA, et al. Features of 20 133 UK patients in hospital with covid-19 using the ISARIC WHO Clinical Characterisation Protocol: prospective observational cohort study. *BMJ*. 2020;369:m1985. doi:10.1136/bmj.m1985.
28. Fernández-Niño JA, Guerra-Gómez JA, Idrovo AJ. Multimorbidity patterns among COVID-19 deaths: proposal for the construction of etiological models. *Rev Panam Salud Publica*. 2020;44:e166. doi:10.26633/RPSP.2020.166.
29. Williamson EJ, Walker AJ, Bhaskaran K, et al. Factors associated with COVID-19-related death using OpenSAFELY. *Nature*. 2020;584(7821):430-436. doi:10.1038/s41586-020-2521-4.
30. Klang E, Kassim G, Soffer S, et al. Morbid Obesity as an Independent Risk Factor for COVID-19 Mortality in Hospitalized Patients Younger than 50. *Obesity (Silver Spring)*. 2020;28(9):1595-1599. doi:10.1002/oby.22913.
31. Cantarino L, Merchan-Hamann E. Influenza in Brazil: surveillance pathways. *J Infect Dev Ctries*. 2016;10(1):13-23. doi:10.3855/jidc.7135.
32. de Souza WM, Buss LF, Candido DDS, et al. Epidemiological and clinical characteristics of the COVID-19 epidemic in Brazil. *Nat Hum Behav*. 2020;4(8):856-865. doi:10.1038/s41562-020-0928-4.
33. Baqui P, Bica I, Marra V, Ercole A, van der Schaar M. Ethnic and regional variations in hospital mortality from COVID-19 in Brazil: a cross-sectional observational study. *Lancet Glob Health*. 2020;8(8):e1018-e1026. doi: 10.1016/S2214-109X(20)30285-0.

For peer review only - http://bmjopen.bmj.com/site/about/guidelines.xhtml

Figure legends

Figure 1. Selection of the study population from SIVEP-Gripe.

SIVEP-Gripe: Influenza Epidemiological Surveillance Information System.

For peer review only

Table 1. Demographic characteristics, comorbidities, hospitalization outcomes and death according to the combined exposure of obesity, diabetes, and/or cardiovascular diseases in adults with severe COVID-19.

	Total		None		OB		OB + DM and/or CVD		DM and/or CVD	
	n	%	n	%	n	%	n	%	n	%
Overall	8848	100.0	3161	35.7	409	4.6	452	5.1	4826	54.6
**Sex**										
Female	3774	42.7	1511	40.0	165	4.4	199	5.3	1899	50.3
Male	5074	57.4	1650	32.5	244	4.8	253	5.0	2927	57.7
**Age**										
< 40 years	1976	22.3	1064	53.9	188	9.5	102	5.2	622	31.5
≥ 40 years	6872	77.7	2097	30.5	221	3.2	350	5.1	4204	61.2
**Chronic pulmonary disease**										
No	8502	96.1	2969	34.9	388	4.6	435	5.1	4710	55.4
Yes	346	3.9	192	55.5	21	6.1	17	4.9	116	33.5
**Asthma**										
No	8184	92.5	2728	33.3	383	4.7	414	5.1	4659	56.9
Yes	664	7.5	433	65.2	26	3.9	38	5.7	167	25.2
**Chronic kidney disease**										
No	8297	93.8	2958	35.7	399	4.8	434	5.2	4506	54.3
Yes	551	6.2	203	36.8	10	1.8	18	3.3	320	58.1
**Chronic hematologic disease**										
No	8710	98.4	3081	35.4	406	4.7	445	5.1	4778	54.9
Yes	138	1.6	80	58.0	3	2.2	7	5.1	48	34.8
**Chronic neurological disease**										
No	8588	97.1	3014	35.1	406	4.7	442	5.2	4726	55.0
Yes	260	2.9	147	56.5	3	1.2	10	3.9	100	38.5
**Chronic liver disease**										
No	8684	98.2	3083	35.5	406	4.7	443	5.1	4752	54.7
Yes	164	1.9	78	47.6	3	1.8	9	5.5	74	45.1
**Immunosuppression**										
No	8276	93.5	2777	33.6	393	4.8	440	5.3	4666	56.4
Yes	572	6.5	384	67.1	16	2.8	12	2.1	160	28.0
**Mechanical ventilation***										
No	2727	33.8	1144	42.0	93	3.4	88	3.2	1402	51.4
Non-invasive	3634	45.0	1178	32.4	192	5.3	190	5.2	2074	57.1
Invasive	1714	21.2	529	30.9	101	5.9	150	8.8	934	54.5
**ICU admission***										
No	5438	64.6	2025	37.2	235	4.3	222	4.1	2956	54.4
Yes	2976	35.4	1007	33.8	163	5.5	216	7.3	1590	53.4
**Death***										
No	4525	68.9	1699	37.6	211	4.7	200	4.4	2415	53.4
Yes	2040	31.1	640	31.4	92	4.5	140	6.9	1168	57.3

OB: obesity (BMI≥30 kg/m²), DM: diabetes mellitus, CVD: cardiovascular disease, ICU: intensive care unit.
* Mechanical ventilation (n=8075), ICU admission (n=8414), and death (n=6565).

For peer review only

Table 2. Demographic characteristics, comorbidities, hospitalization outcomes and death according to the combined exposure of obesity, diabetes, and/or cardiovascular diseases in elders with severe COVID-19.

	Total		None		OB		OB + DM and/or CVD		DM and/or CVD	
	n	%	n	%	n	%	n	%	n	%
Overall	12925	100.0	2837	21.9	91	0.7	358	2.8	9639	74.6
**Sex**										
Female	5968	46.2	1232	20.6	52	0.9	209	3.5	4475	75.0
Male	6957	53.8	1605	23.1	39	0.6	149	2.1	5164	74.2
**Age**										
< 80 years	9355	72.4	2011	21.5	77	0.8	309	3.3	6958	74.4
≥ 80 years	3570	27.6	826	23.1	14	0.4	49	1.4	2681	75.1
**Chronic pulmonary disease**										
No	11885	92.0	2494	21.0	85	0.7	325	2.7	8981	75.6
Yes	1040	8.1	343	33.0	6	0.6	33	3.2	658	63.3
**Asthma**										
No	12474	96.5	2687	21.5	90	0.7	336	2.7	9361	75.0
Yes	451	3.5	150	33.3	1	0.2	22	4.9	278	61.6
**Chronic kidney disease**										
No	11882	91.9	2608	22.0	85	0.7	311	2.6	8878	74.7
Yes	1043	8.1	229	22.0	6	0.6	47	4.5	761	73.0
**Chronic hematologic disease**										
No	12728	98.5	2751	21.6	91	0.7	354	2.8	9532	74.9
Yes	197	1.5	86	43.7	0	0.0	4	2.0	107	54.3
**Chronic neurological disease**										
No	11871	91.9	2511	21.2	89	0.8	338	2.9	8933	75.3
Yes	1054	8.2	326	30.9	2	0.2	20	1.9	706	67.0
**Chronic liver disease**										
No	12734	98.5	2777	21.8	87	0.7	353	2.8	9517	74.7
Yes	191	1.5	60	31.4	4	2.1	5	2.6	122	63.9
**Immunosuppression**										
No	12303	95.2	2558	20.8	87	0.7	342	2.8	9316	75.7
Yes	622	4.8	279	44.9	4	0.6	16	2.6	323	51.9
**Mechanical ventilation***										
No	2725	23.0	626	23.0	18	0.7	70	2.6	2011	73.8
Non-invasive	5557	47.0	1164	21.0	38	0.7	141	2.5	4214	75.8
Invasive	3547	30.0	767	21.6	29	0.8	133	3.8	2618	73.8
**ICU admission***										
No	6898	56.4	1578	22.9	41	0.6	168	2.4	5111	74.1
Yes	5324	43.6	1107	20.8	44	0.8	181	3.4	3992	75.0
**Death***										
No	3684	37.1	823	22.3	21	0.6	95	2.6	2745	74.5
Yes	6259	63.0	1407	22.5	43	0.7	173	2.8	4636	74.1

OB: obesity (BMI≥30 kg/m^2), DM: diabetes mellitus, CVD: cardiovascular disease, ICU: intensive care unit.
* Mechanical ventilation (n=11829), ICU admission (n=12222), and death (n=9943).

For peer review only

Table 3. Combined association of obesity, diabetes, and/or cardiovascular disease with non-invasive and invasive mechanical ventilation use, intensive care unit admission, and death in adult and elderly patients hospitalized with severe COVID-19.

	Main exposure variable	Non-invasive mechanical ventilation*				Invasive mechanical ventilation*			
		Crude model		Adjusted model #		Crude model		Adjusted model #	
		PR	95%CI	PR	95%CI	PR	95%CI	PR	95%CI
**Adults** 20-59 years	None	1.00		1.00		1.00		1.00	
	OB	2.00	1.54-2.60	2.13	1.64-2.78	2.35	1.74-3.17	2.69	1.98-3.65
	OB + DM and/or CVD	2.10	1.61-2.73	2.06	1.58-2.69	3.69	2.78-4.89	3.76	2.82-5.01
	DM and/or CVD	1.44	1.29-1.60	1.35	1.20-1.51	1.44	1.26-1.64	1.32	1.14-1.52
**Elders** ≥ 60 years	None	1.00		1.00		1.00		1.00	
	OB	1.14	0.64-2.01	1.22	0.69-2.16	1.31	0.72-2.39	1.43	0.78-2.61
	OB + DM and/or CVD	1.08	0.80-1.47	1.15	0.84-1.55	1.55	1.14-2.11	1.66	1.22-2.27
	DM and/or CVD	1.13	1.01-1.26	1.14	1.01-1.27	1.06	0.94-1.20	1.10	0.97-1.24
		**ICU admission****				**Death*****			
		Crude model		Adjusted model #		Crude model		Adjusted model #	
		PR	95%CI	PR	95%CI	PR	95%CI	PR	95%CI
**Adults** 20-59 years	None	1.00		1.00		1.00		1.00	
	OB	1.23	1.08-1.40	1.31	1.13-1.53	1.11	0.92-1.33	1.33	1.05-1.69
	OB + DM and/or CVD	1.48	1.33-1.65	1.60	1.40-1.83	1.50	1.30-1.74	1.79	1.45-2.21
	DM and/or CVD	1.05	0.99-1.12	1.03	0.95-1.12	1.19	1.10-1.29	1.16	1.03-1.30
**Elders** ≥ 60 years	None	1.00		1.00		1.00		1.00	
	OB	1.26	1.02-1.55	1.40	1.07-1.82	1.06	0.89-1.27	1.67	1.00-2.80
	OB + DM and/or CVD	1.26	1.13-1.41	1.37	1.19-1.59	1.02	0.93-1.12	1.39	1.07-1.80
	DM and/or CVD	1.06	1.01-1.12	1.11	1.04-1.18	1.00	0.96-1.03	1.05	0.95-1.16

OB: obesity (BMI≥30 kg/m^2), DM: diabetes mellitus, CVD: cardiovascular disease, ICU: intensive care unit, PR: prevalence ratio, 95%CI: 95% confidence interval.
* Crude and adjusted multinomial logistic regression models for mechanical ventilation use in adults (n=8075) and elders (n=11829).
** Crude and adjusted logistic regression models for ICU admission in adults (n= 8414) and elders (n=12222).
*** Crude and adjusted logistic regression models for death in adults (n=6565) and elders (n=9943).
# Adjusted for sex, age in years, pulmonary disease, asthma, kidney disease, hematologic disease, neurological disease, liver disease, and immunosuppression.

For peer review only

Table 4. Independent association of degrees of obesity with non-invasive and invasive mechanical ventilation, intensive care unit admission, and death in hospitalized adults with severe COVID-19.

Main exposure variable	Non-invasive mechanical ventilation*				Invasive mechanical ventilation*			
	Crude model		Adjusted model #		Crude model		Adjusted model #	
	PR	95%CI	PR	95%CI	PR	95%CI	PR	95%CI
No obesity (< 30 kg/m^2)	1.00		1.00		1.00		1.00	
Obesity class I (≥ 30-34.9 kg/m^2)	1.78	1.35-2.33	1.91	1.45-2.51	2.59	1.93-3.47	3.00	2.22-4.05
Obesity class II (≥ 35-39.9 kg/m^2)	1.44	1.04-2.00	1.58	1.14-2.19	2.10	1.47-2.99	2.47	1.72-3.54
Obesity class III (≥ 40 kg/m^2)	1.70	1.19-2.44	1.88	1.31-2.69	2.51	1.71-3.70	3.00	2.03-4.45
	**ICU admission****				**Death****			
	Crude model		Adjusted model #		Crude model		Adjusted model #	
	PR	95%CI	PR	95%CI	PR	95%CI	PR	95%CI
No obesity (< 30 kg/m^2)	1.00		1.00		1.00		1.00	
Obesity class I (≥ 30-34.9 kg/m^2)	1.31	1.17-1.47	1.42	1.23-1.64	1.11	0.94-1.31	1.32	1.05-1.66
Obesity class II (≥ 35-39.9 kg/m^2)	1.34	1.16-1.54	1.46	1.23-1.74	1.16	0.95-1.42	1.41	1.06-1.87
Obesity class III (≥ 40 kg/m^2)	1.32	1.14-1.54	1.45	1.20-1.74	1.33	1.10-1.59	1.77	1.35-2.33

Degrees of obesity defined by the WHO cutoff points.
PR: prevalence ratio, 95%CI: 95% confidence interval.
* Crude and adjusted multinomial logistic regression models for mechanical ventilation use (n=8075).
** Crude and adjusted logistic regression models for ICU admission (n=8414) and mortality (n=6565).
# Adjusted for sex, age in years, diabetes mellitus, cardiovascular disease, pulmonary disease, asthma, kidney disease, hematologic disease, neurological disease, liver disease, and immunosuppression.

For peer review only

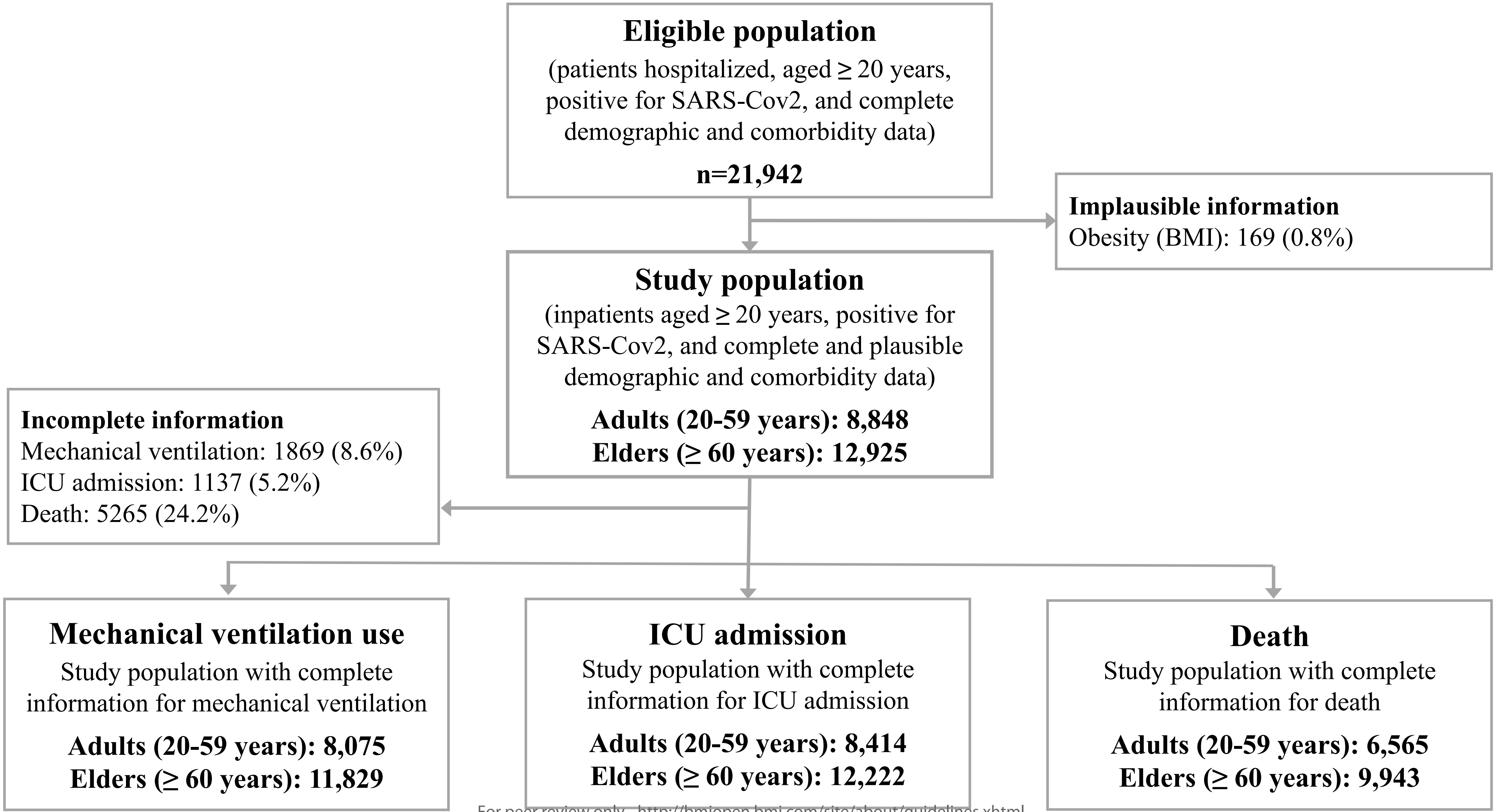
Eligible population
(patients hospitalized, aged ≥ 20 years, positive for SARS-Cov2, and complete demographic and comorbidity data)
n=21,942
Implausible information
Obesity (BMI): 169 (0.8%)
Study population
(inpatients aged ≥ 20 years, positive for SARS-Cov2, and complete and plausible demographic and comorbidity data)
Adults (20-59 years): 8,848
Elders (≥ 60 years): 12,925
Incomplete information
Mechanical ventilation: 1869 (8.6%)
ICU admission: 1137 (5.2%)
Death: 5265 (24.2%)
Mechanical ventilation use
Study population with complete information for mechanical ventilation
Adults (20-59 years): 8,075
Elders (≥ 60 years): 11,829
ICU admission
Study population with complete information for ICU admission
Adults (20-59 years): 8,414
Elders (≥ 60 years): 12,222
Death
Study population with complete information for death
Adults (20-59 years): 6,565
Elders (≥ 60 years): 9,943

Suppl. Table 1. Characteristics of the study population and study samples included and excluded of the analysis for each severe COVID-19 outcome.

	Study population*		Mechanical ventilation				ICU admission				Death			
			Included		Excluded		Included		Excluded		Included		Excluded	
	n	%	n	%	n	%	n	%	n	%	n	%	n	%
Overall	21,773	100.0	19,904	91.4	1,869	8.6	20,636	94.8	1,137	5.2	16,508	75.8	5,265	24.2
**Sex**														
Female	9,742	44.7	8,905	44.7	837	44.8	9,227	44.7	515	45.3	7,379	44.7	2,363	44.9
Male	12,031	55.3	10,999	55.3	1,032	55.2	11,409	55.3	622	54.7	9,129	55.3	2,902	55.1
**Age (years)**														
20-39	1,976	9.1	1,808	9.1	168	9.0	1,890	9.2	86	7.6	1,445	8.8	531	10.1
40-59	6,872	31.6	6,267	31.5	605	32.4	6,524	31.6	348	30.6	5,120	31.0	1,752	33.3
60-79	9,355	43.0	8,546	42.9	809	43.3	8,827	42.8	528	46.4	7,062	42.8	2,293	43.6
>= 80	3,570	16.4	3,283	16.5	287	15.4	3,395	16.5	175	15.4	2,881	17.5	689	13.1
**Obesity**														
No	20,463	94.0	18,661	93.8	1,802	96.4	19,366	93.9	1,097	96.5	15,533	94.1	4,930	93.6
Yes	1,310	6.0	1,243	6.2	67	3.6	1,270	6.2	40	3.5	975	5.9	335	6.4
**Diabetes**														
No	13,058	60.0	11,925	59.9	1,133	60.6	12,401	60.1	657	57.8	9,925	60.1	3,133	59.5
Yes	8,715	40.0	7,979	40.1	736	39.4	8,235	39.9	480	42.2	6,583	39.9	2,132	40.5
**Cardiovascular disease**														
No	10,391	47.7	9,400	47.2	991	53.0	9,858	47.8	533	46.9	7,866	47.7	2,525	48.0
Yes	11,382	52.3	10,504	52.8	878	47.0	10,778	52.2	604	53.1	8,642	52.4	2,740	52.0
**Chronic pulmonary disease**														
No	20,387	93.6	18,630	93.6	1,757	94.0	19,306	93.6	1,081	95.1	15,422	93.4	4,965	94.3
Yes	1,386	6.4	1,274	6.4	112	6.0	1,330	6.5	56	4.9	1,086	6.6	300	5.7
**Asthma**														
No	20,658	94.9	18,897	94.9	1,761	94.2	19,571	94.8	1,087	95.6	15,666	94.9	4,992	94.8
Yes	1,115	5.1	1,007	5.1	108	5.8	1,065	5.2	50	4.4	842	5.1	273	5.2
**Chronic kidney disease**														
No	20,179	92.7	18,420	92.5	1,759	94.1	19,106	92.6	1,073	94.4	15,255	92.4	4,924	93.5
Yes	1,594	7.3	1,484	7.5	110	5.9	1,530	7.4	64	5.6	1,253	7.6	341	6.5
**Chronic hematologic disease**														
No	21,438	98.5	19,597	98.5	1,841	98.5	20,313	98.4	1,125	98.9	16,245	98.4	5,193	98.6
Yes	335	1.5	307	1.5	28	1.5	323	1.6	12	1.1	263	1.6	72	1.4
**Chronic neurological disease**														
No	20,459	94.0	18,700	94.0	1,759	94.1	19,377	93.9	1,082	95.2	15,460	93.7	4,999	95.0
Yes	1,314	6.0	1,204	6.1	110	5.9	1,259	6.1	55	4.8	1,048	6.4	266	5.1

For peer review only

**Chronic liver disease**																
No	21,418	98.4	19,581	98.4	1,837	98.3	20,300	98.4	1,118	98.3	16,218	98.2	5,200	98.8		
Yes	355	1.6	323	1.6	32	1.7	336	1.6	19	1.7	290	1.8	65	1.2		
**Immunosuppression**																
No	20,579	94.5	18,792	94.4	1,787	95.6	19,485	94.4	1,094	96.2	15,571	94.3	5,008	95.1		
Yes	1,194	5.5	1,112	5.6	82	4.4	1,151	5.6	43	3.8	937	5.7	257	4.9		

For peer review only

The RECORD statement – checklist of items, extended from the STROBE statement, that should be reported in observational studies using routinely collected health data.

	Item No.	STROBE items	Location in manuscript where items are reported	RECORD items	Location in manuscript where items are reported
**Title and abstract**					
	1	(a) Indicate the study's design with a commonly used term in the title or the abstract (b) Provide in the abstract an informative and balanced summary of what was done and what was found		RECORD 1.1: The type of data used should be specified in the title or abstract. When possible, the name of the databases used should be included.<br><br>RECORD 1.2: If applicable, the geographic region and timeframe within which the study took place should be reported in the title or abstract.<br><br>RECORD 1.3: If linkage between databases was conducted for the study, this should be clearly stated in the title or abstract.	Page 1-2<br><br>Page 1-2<br><br>N/A
**Introduction**					
Background rationale	2	Explain the scientific background and rationale for the investigation being reported			Pages 4-5
Objectives	3	State specific objectives, including any prespecified hypotheses			Pages 4-5
**Methods**					
Study Design	4	Present key elements of study design early in the paper			Page 5
Setting	5	Describe the setting, locations, and relevant dates, including periods of recruitment, exposure, follow-up, and data collection			Page 5

For peer review only

Participants	6	*(a) Cohort study* - Give the eligibility criteria, and the sources and methods of selection of participants. Describe methods of follow-up<br>*Case-control study* - Give the eligibility criteria, and the sources and methods of case ascertainment and control selection. Give the rationale for the choice of cases and controls<br>*Cross-sectional study* - Give the eligibility criteria, and the sources and methods of selection of participants<br><br>*(b) Cohort study* - For matched studies, give matching criteria and number of exposed and unexposed<br>*Case-control study* - For matched studies, give matching criteria and the number of controls per case		RECORD 6.1: The methods of study population selection (such as codes or algorithms used to identify subjects) should be listed in detail. If this is not possible, an explanation should be provided.<br><br>RECORD 6.2: Any validation studies of the codes or algorithms used to select the population should be referenced. If validation was conducted for this study and not published elsewhere, detailed methods and results should be provided.<br><br>RECORD 6.3: If the study involved linkage of databases, consider use of a flow diagram or other graphical display to demonstrate the data linkage process, including the number of individuals with linked data at each stage.	Page 5
Variables	7	Clearly define all outcomes, exposures, predictors, potential confounders, and effect modifiers. Give diagnostic criteria, if applicable.		RECORD 7.1: A complete list of codes and algorithms used to classify exposures, outcomes, confounders, and effect modifiers should be provided. If these cannot be reported, an explanation should be provided.	Pages 5-6
Data sources/ measurement	8	For each variable of interest, give sources of data and details of methods of assessment (measurement).<br>Describe comparability of assessment methods if there is more than one group			Pages 5-6

For peer review only

Bias	9	Describe any efforts to address potential sources of bias			Pages 11-12
Study size	10	Explain how the study size was arrived at			Page 5
Quantitative variables	11	Explain how quantitative variables were handled in the analyses. If applicable, describe which groupings were chosen, and why			Pages 5-6
Statistical methods	12	(a) Describe all statistical methods, including those used to control for confounding<br>(b) Describe any methods used to examine subgroups and interactions<br>(c) Explain how missing data were addressed<br>(d) *Cohort study* - If applicable, explain how loss to follow-up was addressed<br>*Case-control study* - If applicable, explain how matching of cases and controls was addressed<br>*Cross-sectional study* - If applicable, describe analytical methods taking account of sampling strategy<br>(e) Describe any sensitivity analyses			Pages 6-7
Data access and cleaning methods		..		RECORD 12.1: Authors should describe the extent to which the investigators had access to the database population used to create the study population.	Page 5

For peer review only

				RECORD 12.2: Authors should provide information on the data cleaning methods used in the study.	
Linkage		..		RECORD 12.3: State whether the study included person-level, institutional-level, or other data linkage across two or more databases. The methods of linkage and methods of linkage quality evaluation should be provided.	N/A
**Results**					
Participants	13	(a) Report the numbers of individuals at each stage of the study (*e.g.*, numbers potentially eligible, examined for eligibility, confirmed eligible, included in the study, completing follow-up, and analysed)<br>(b) Give reasons for non-participation at each stage.<br>(c) Consider use of a flow diagram		RECORD 13.1: Describe in detail the selection of the persons included in the study (*i.e.,* study population selection) including filtering based on data quality, data availability and linkage. The selection of included persons can be described in the text and/or by means of the study flow diagram.	Page 7
Descriptive data	14	(a) Give characteristics of study participants (*e.g.*, demographic, clinical, social) and information on exposures and potential confounders<br>(b) Indicate the number of participants with missing data for each variable of interest<br>(c) *Cohort study* - summarise follow-up time (*e.g.*, average and total amount)			Page 7
Outcome data	15	*Cohort study* - Report numbers of outcome events or summary measures over time<br>*Case-control study* - Report numbers in each exposure			Pages 7-8

For peer review only

		category, or summary measures of exposure<br>*Cross-sectional study* - Report numbers of outcome events or summary measures			
Main results	16	(a) Give unadjusted estimates and, if applicable, confounder-adjusted estimates and their precision (e.g., 95% confidence interval). Make clear which confounders were adjusted for and why they were included<br>(b) Report category boundaries when continuous variables were categorized<br>(c) If relevant, consider translating estimates of relative risk into absolute risk for a meaningful time period			Pages 8
Other analyses	17	Report other analyses done—e.g., analyses of subgroups and interactions, and sensitivity analyses			Page 8
**Discussion**					
Key results	18	Summarise key results with reference to study objectives			Page 8-9
Limitations	19	Discuss limitations of the study, taking into account sources of potential bias or imprecision. Discuss both direction and magnitude of any potential bias		RECORD 19.1: Discuss the implications of using data that were not created or collected to answer the specific research question(s). Include discussion of misclassification bias, unmeasured confounding, missing data, and changing eligibility over time, as they pertain to the study being reported.	Page 11-12
Interpretation	20	Give a cautious overall interpretation of results considering objectives,			Pages 9-11

For peer review only

		limitations, multiplicity of analyses, results from similar studies, and other relevant evidence			
Generalisability	21	Discuss the generalisability (external validity) of the study results			Page 12
**Other Information**					
Funding	22	Give the source of funding and the role of the funders for the present study and, if applicable, for the original study on which the present article is based			Page 13
Accessibility of protocol, raw data, and programming code		..		RECORD 22.1: Authors should provide information on how to access any supplemental information such as the study protocol, raw data, or programming code.	Page 13

*Reference: Benchimol EI, Smeeth L, Guttmann A, Harron K, Moher D, Petersen I, Sørensen HT, von Elm E, Langan SM, the RECORD Working Committee. The REporting of studies Conducted using Observational Routinely-collected health Data (RECORD) Statement. *PLoS Medicine* 2015; in press.

*Checklist is protected under Creative Commons Attribution (CC BY) license.

For peer review only

BMJ Open

Combined association of obesity and other cardiometabolic diseases with severe COVID-19 outcomes: a nationwide cross-sectional study of 21,773 Brazilian adult and elderly inpatients

Journal:	*BMJ Open*
Manuscript ID	bmjopen-2021-050739.R2
Article Type:	Original research
Date Submitted by the Author:	24-Jun-2021
Complete List of Authors:	Silva, Natanael; Oswaldo Cruz Foundation, Centre for Data and Knowledge Integration for Health<br>Silva, Rita de Cássia; Federal University of Bahia, School of Nutrition; Oswaldo Cruz Foundation, Centre for Data and Knowledge Integration for Health<br>Ferreira, Andrêa; Federal University of Bahia, Institute of Collective Health; Oswaldo Cruz Foundation, Centre for Data and Knowledge Integration for Health<br>Teixeira, Camila; Federal University of Bahia, Institute of Collective Health; Oswaldo Cruz Foundation, Centre for Data and Knowledge Integration for Health<br>Rocha, Aline; Federal University of Bahia, School of Nutrition; Oswaldo Cruz Foundation, Centre for Data and Knowledge Integration for Health<br>Alves, Flávia Jôse; Federal University of Bahia, Institute of Collective Health; Oswaldo Cruz Foundation, Centre for Data and Knowledge Integration for Health<br>Falcão, Ila; Oswaldo Cruz Foundation, Centre for Data and Knowledge Integration for Health<br>Pinto, Elizabete; Federal University of Reconcavo da Bahia, Center for Health Sciences; Oswaldo Cruz Foundation, Centre for Data and Knowledge Integration for Health<br>Santos, Carlos Antônio; Oswaldo Cruz Foundation, Centre for Data and Knowledge Integration for Health; State University of Feira de Santana, Department of Exact Sciences<br>Fiaccone, Rosemeire; Federal University of Bahia, Institute of Mathematics and Statistics; Oswaldo Cruz Foundation, Centre for Data and Knowledge Integration for Health<br>Ichihara, Maria Yury; Oswaldo Cruz Foundation, Centre for Data and Knowledge Integration for Health<br>Paixão, Enny; London School of Hygiene and Tropical Medicine Faculty of Epidemiology and Population Health; Oswaldo Cruz Foundation, Centre for Data and Knowledge Integration for Health<br>Barreto, Mauricio; Oswaldo Cruz Foundation, Centre for Data and Knowledge Integration for Health; Federal University of Bahia, Institute of Collective Health
**Primary Subject Heading**:	Epidemiology

Secondary Subject Heading:	Infectious diseases, Nutrition and metabolism
Keywords:	COVID-19, INFECTIOUS DISEASES, INTENSIVE & CRITICAL CARE, DIABETES & ENDOCRINOLOGY, NUTRITION & DIETETICS, EPIDEMIOLOGY

SCHOLARONE™ Manuscripts

I, the Submitting Author has the right to grant and does grant on behalf of all authors of the Work (as defined in the below author licence), an exclusive licence and/or a non-exclusive licence for contributions from authors who are: i) UK Crown employees; ii) where BMJ has agreed a CC-BY licence shall apply, and/or iii) in accordance with the terms applicable for US Federal Government officers or employees acting as part of their official duties; on a worldwide, perpetual, irrevocable, royalty-free basis to BMJ Publishing Group Ltd ("BMJ") its licensees and where the relevant Journal is co-owned by BMJ to the co-owners of the Journal, to publish the Work in this journal and any other BMJ products and to exploit all rights, as set out in our licence.

The Submitting Author accepts and understands that any supply made under these terms is made by BMJ to the Submitting Author unless you are acting as an employee on behalf of your employer or a postgraduate student of an affiliated institution which is paying any applicable article publishing charge ("APC") for Open Access articles. Where the Submitting Author wishes to make the Work available on an Open Access basis (and intends to pay the relevant APC), the terms of reuse of such Open Access shall be governed by a Creative Commons licence – details of these licences and which Creative Commons licence will apply to this Work are set out in our licence referred to above.

Other than as permitted in any relevant BMJ Author's Self Archiving Policies, I confirm this Work has not been accepted for publication elsewhere, is not being considered for publication elsewhere and does not duplicate material already published. I confirm all authors consent to publication of this Work and authorise the granting of this licence.

For peer review only

Combined association of obesity and other cardiometabolic diseases with severe COVID-19 outcomes: a nationwide cross-sectional study of 21,773 Brazilian adult and elderly inpatients

Natanael J Silva, MSc[1,2]; Rita C Ribeiro-Silva, PhD[1,2,3]; Andrêa JF Ferreira, MSc[1,2,4]; Camila SS Teixeira, MSc[1,2,4]; Aline S Rocha, MSc[1,2,3]; Flávia Jôse O Alves, MSc[1,2,4]; Ila R Falcão, PhD[2]; Elizabete J Pinto, PhD[2,5]; Carlos Antônio ST Santos, PhD[1,2,6]; Rosemeire L Fiaccone, PhD[1,2,7]; Maria Yury T Ichihara, MD PhD[1,2]; Enny S Paixão, PhD[1,2,8]; Mauricio L Barreto, MD PhD[1,2,4]

[1] Rede CoVida, Salvador, BA, Brazil

[2] Centre for Data and Knowledge Integration for Health, Oswaldo Cruz Foundation, Salvador, BA, Brazil

[3] School of Nutrition, Federal University of Bahia, Salvador, BA, Brazil

[4] Institute of Collective Health, Federal University of Bahia, Salvador, BA, Brazil

[5] Center for Health Sciences, Federal University of Recôncavo da Bahia, Santo Antônio de Jesus, BA, Brazil

[6] Department of Exact Sciences, State University of Feira de Santana, Feira de Santana, BA, Brazil

[7] Institute of Mathematics and Statistics, Federal University of Bahia, Salvador, BA, Brazil

[8] Epidemiology and Population Health, London School of Hygiene and Tropical Medicine, London, UK

Corresponding author: Natanael J Silva. Centre for Data and Knowledge Integration for Health, Oswaldo Cruz Foundation. Edf. Tecnocentro, Sl 315. Rua Mundo, 121. Trobogy, Salvador, BA, 41745-715, Brazil. Phone number: +55 79 99839-3991. Email: <silva_natanael@hotmail.com>.

Keywords: Obesity, COVID-19, SARS-CoV-2, Hospitalization, Mortality.

Word count: 3,349

For peer review only

Abstract

Objectives: To investigate the combined association of obesity, diabetes mellitus (DM), and cardiovascular disease (CVD) with severe COVID-19 outcomes in adult and elderly inpatients.

Design: Cross-sectional study based on registry data from Brazil's influenza surveillance system.

Setting: Public and private hospitals across Brazil.

Participants: Eligible population included 21,942 inpatients aged ≥20 years with positive RT-PCR test for SARS-CoV-2 until Jun 9th, 2020.

Main outcome measures: Severe COVID-19 outcomes were non-invasive and invasive mechanical ventilation use, ICU admission, and death. Multivariate analyses were conducted separately for adults (20-59 years) and elders (≥60 years) to test the combined association of obesity (without and with DM and/or CVD) and degrees of obesity with each outcome.

Results: A sample of 8,848 adults and 12,925 elders were included. Among adults, obesity with DM and/or CVD showed higher prevalence of invasive (PR 3.76, 95%CI 2.82-5.01) and non-invasive mechanical ventilation use (2.06, 1.58-2.69), ICU admission (1.60, 1.40-1.83), and death (1.79, 1.45-2.21) compared with the group without obesity, DM, and CVD. In elders, obesity alone (without DM and CVD) had the highest prevalence of ICU admission (1.40, 1.07-1.82) and death (1.67, 1.00-2.80). In both age groups, obesity alone and combined with DM and/or CVD showed higher prevalence in all outcomes than DM and/or CVD. A dose-response association was observed between obesity and death in adults: class I 1.32 (1.05-1.66), class II 1.41 (1.06-1.87), and class III 1.77 (1.35-2.33).

Conclusions: The combined association of obesity, diabetes, and/or CVD with severe COVID-19 outcomes may be stronger in adults than in elders. Obesity alone and combined with DM and/or CVD had more impact on the risk of COVID-19 severity than DM and/or CVD in both age groups. The study also supports an independent relationship of obesity with severe outcomes, including a dose-response association between degrees of obesity and death in adults.

Article summary

Strengths and limitations of this study:

- This is the first study that describes the independent and combined relationship of obesity with COVID-19 severity in Brazil, one of the biggest epicenters of the pandemic worldwide.
- The study was based on registry data of a large nationwide sample of patients admitted, due to severe SARS-CoV-2 infection, to public and private hospitals across the country.
- The large sample size and data availability allowed us to analyze the combined association of obesity, diabetes and cardiovascular disease with severe COVID-19 outcomes, separately by age groups and controlled by important confounding variables, e.g. underlying comorbidities.
- The cross-sectional study design does not allow causal inference, and generalization of results must be cautious since only hospitalized cases of severe COVID-19 were included.
- As the study used routinely collected data, which has not been designed primarily for research purposes, it may bring well-known limitations related to missing, underestimation, and potential misclassification.

For peer review only

Introduction

The coronavirus disease 2019 (COVID-19) pandemic, caused by the severe acute respiratory syndrome coronavirus 2 (SARS-CoV-2), as of 13 May 2021, has already reached more than 160 million infected people and more than 3.3 million deaths in all continents.[1] Individuals with advanced age and chronic diseases, including cardiometabolic diseases, are considered groups at major risk for complications and severe illness from COVID-19.[2,3] Obesity has been shown as an independent risk factor for COVID-19 disease.[4-6] High body mass index (BMI) has been mentioned as a significant risk factor for COVID-19, according to early clinical reports from China,[7] Italy,[8] France,[9] Mexico,[10] and United States of America.[11] Several studies have demonstrated that obesity is leading to considerably worse COVID-19 outcomes, especially greater risk of hospital and intensive care unit (ICU) admission, invasive mechanical ventilation, and death.[11-14]

The COVID-19 pandemic is rapidly spreading worldwide, especially in the Americas, where obesity is already a prevalent and important public health problem.[15-16] Brazil is currently one of the biggest epicenters of the COVID-19 pandemic worldwide, with more than 15.2 million cases and 425 thousand deaths until May 13, 2021.[1] In 2018, the prevalence of adult overweight and obesity in Brazil was estimated at 55.7% and 19.8%, respectively.[17] This obesogenic profile of the Brazilian population contributes, among other factors, to the high prevalence of obesity-related diseases such as type 2 diabetes mellitus (DM) and cardiovascular diseases (CVD), in the country.[18] The fact that individuals with obesity also have more comorbidity diseases, which are either risk factors for COVID-19 severity and death, makes obesity particularly ominous in COVID-19 disease.[10-13]

Several characteristics that can influence the clinical evolution of individuals infected with COVID-19, such as obesity, have been independently documented.[5-6,19] However, evidence is yet unclear on the combined effect that obesity and obesity-related comorbidities play in COVID-19 severity, especially, in different age groups. We aimed in this study to investigate the combined association of obesity, diabetes, and cardiovascular disease with mechanical ventilation use, ICU admission, and

For peer review only

death in a large sample of adult and elderly patients hospitalized with COVID-19 in Brazil. We also explored the independent association between degrees of obesity and the mentioned outcomes.

Methods

Study Design and Population

This is a cross-sectional study based on registry data from SIVEP-Gripe (Sistema de Informação de Vigilância Epidemiológica da Gripe), an influenza surveillance system of Brazil's Ministry of Health. The study used the publicly available dataset of SIVEP-Gripe, which includes de-identified data on cases of severe acute respiratory syndrome across public and private hospitals in Brazil.[20] These data were obtained through the Rede CoVida's integrated data platform that has been built with official, open, and authorized data for the production of knowledge about the COVID-19 pandemic.

Our study population was composed of patients aged 20 years or older, hospitalized for severe acute respiratory syndrome, with positive RT-PCR test for SARS-CoV-2, and final diagnosis for COVID-19 until Jun 9th, 2020. Only cases with complete data on demographic characteristics and comorbidities and plausible BMI values were included in the study.

Exposure Variable

Obesity was defined as BMI equal to or greater than 30 kg/m^2, according to the cutoff points proposed by the World Health Organization[21] and the Pan American Health Organization[22] for adults and elders, respectively. BMI was calculated by health professionals in the hospital from directly measured or patient self-reported height and weight. Guidelines for the collection and analysis of anthropometric data in health services have been previously standardized by the Ministry of Health.[23] BMI values <12 or >70 kg/m^2 were considered implausible and excluded.[24] Because BMI in the SIVEP-Gripe is a mandatory and complete information for patients diagnosed or self-reported with obesity only, the nutritional status of all patients were confirmed by a dichotomous variable (no/yes) on the existence of obesity available in the dataset. Likewise, information on the existence of diabetes

For peer review only

and any chronic cardiovascular disease was obtained from dichotomous questions (yes/no), which were answered based on patient or family's report or medical diagnosis.

We created a polytomous four-category variable to evaluate the separate and combined exposure of obesity, diabetes and cardiovascular disease: none/reference (no existence of obesity, diabetes and cardiovascular disease), OB (only existence of obesity), OB + DM and/or CVD (existence of obesity with diabetes and/or cardiovascular disease), and DM and/or CVD (existence of diabetes and/or cardiovascular disease). We also analyzed obesity in adults according to the following degrees of severity based on WHO reference[21]: no obesity (<30 kg/m^2), obesity class I (≥ 30-34.9 kg/m^2), obesity class II (≥ 35-39.9 kg/m^2), and obesity class III (≥ 40 kg/m^2). Due to the unavailability of BMI cutoff points to classify the degree of obesity in elders, this analysis was only performed for adults.

Outcome Variables

The severe COVID-19 outcomes were mechanical ventilation use, ICU admission, and death. Information on the use of mechanical ventilation by the patient was obtained and analyzed as a polytomous three-category variable (no use/ use of non-invasive ventilation/ use of invasive ventilation). ICU admission was obtained and analyzed as a dichotomous variable (no/ yes). Death was analyzed as a dichotomous variable based on the patient's endpoint outcome (cure/ death).

Covariates

Demographic and comorbidity information were selected as descriptive and confounding variables.[2] Age in years was calculated from birth and notification dates. Sex was obtained as a dichotomous variable (female/ male). The preexistence of each comorbidity was also obtained as a dichotomous variable (no/ yes): chronic pulmonary disease, asthma, chronic kidney disease, chronic hematologic disease, neurological disease, chronic liver disease, and immunodeficiency/ immunosuppression.

Statistical Analysis

All analyses were subdivided into adults (≥ 20 and < 60 years) and elders (≥ 60 years). For descriptive analyses, absolute and relative frequencies were calculated for the demographic and comorbidity

For peer review only

132 variables according to the main exposure variable. Multinomial logistic regression models were conducted to test the association of obesity (without and with diabetes and/or CVD) with non-invasive and invasive mechanical ventilation use. To test the association of this exposure variable with ICU admission and death, simple logistic regression models were performed. Same models were analyzed considering the degree of obesity as the main exposure variable for adults. Given the high prevalence of the analyzed outcome that could overestimate odds ratio,[25] crude and adjusted estimates were interpreted based on the prevalence ratio (PR) and 95% confidence intervals (95%CI). These estimates were obtained from logistic models using delta method, function 'prLogisticDelta', which is implemented in R and available in the package 'prLogistic'. Adjusted models included a set of confounding variables selected according to the current literature on obesity and severe COVID-9 risk factors:[26,27] sex, age (years), and the preexistence of chronic pulmonary disease, asthma, kidney disease, hematologic disease, neurological disease, liver disease, and immunodeficiency/ immunosuppression. The models that tested the degrees of obesity were also adjusted for DM and CVD. All analyses were performed using Stata version 15.1 (Stata Corporation, College Station, USA) and R version 3.6.1 (R Foundation for Statistical Computing, Austria).

Sensitivity analysis

Because detailed information on the comorbidities were not available (e.g., duration, severity), serious comorbidities such as chronic pulmonary diseases and immunosuppression were tested as exclusion criteria instead confounding variables in a sensitivity analysis. Same multivariate logistic models, using 'death' as outcome variable, were conducted separately for adults and elders, excluding the cases of pulmonary diseases and immunosuppression.

Patient and Public Involvement

As the study exclusively used publicly available de-identified data, it was not possible to involve patients or the public in the design, or conduct, or reporting, or dissemination plans of our research.

Results

For peer review only - http://bmjopen.bmj.com/site/about/guidelines.xhtml

During the study period, 21,942 individuals registered in the SIVEP-Gripe were ≥ 20 years old, hospitalized, tested positive for SARS-CoV-2, and had complete demographic and comorbidity information (**Figure 1**). Of these, 169 (0.8%) were excluded due to implausible values of BMI. Of the 21,773 individuals included in the study, 8,848 (40.3%) were adults aged between 20-59 years, and 12,925 (59.6%) were elders aged 60 years or older. Since some patients were still hospitalized on the study endpoint date, information for some outcomes were incomplete. The study samples included in the analysis of each outcome were 8,075 adults and 11,829 elders for mechanical ventilation, 8,414 adults and 12,222 for ICU admission, and 6,565 adults and 9,943 elders for death. Based on demographic and clinical characteristics, the analytical samples in each outcome were very similar to the overall study population and the excluded samples (**Supplementary Table 1**).

The prevalence of obesity was 9.7% in adults and 3.5% in elders. The frequency of obesity without and with DM and/or CVD was respectively 4.6% and 5.1% in adults and 0.7% and 2.8% in elders. Non-invasive and invasive mechanical ventilation were respectively required by 45.0% and 21.2% of adults and 47.0% and 30.0% of elders. ICU admission was needed by 35.4% of adults and 43.6% of elders. Death occurred in 31.1% of adult and 63.0% of elderly patients (**Table 1-2**).

In the adjusted analyses for adults, obesity alone (without DM and CVD) was associated with an increased prevalence of invasive (PR 2.69, 95%CI 1.98-3.65) and non-invasive mechanical ventilation need (PR 2.13, 95%CI 1.64-2.78), ICU admission (PR 1.31, 95%CI 1.13-1.53), and death (PR 1.33, 95%CI 1.05-1.69) when compared with the group without obesity, DM, and CVD. Obesity with DM and/or CVD was associated with an even higher prevalence of invasive mechanical ventilation (PR 3.76, 95%CI 2.82-5.01) and non-invasive ventilation use (PR 2.06, 95%CI 1.58-2.69), ICU admission (PR 1.60, 95%CI 1.40-1.83), and death in adults (PR 1.79, 95%CI 1.45-2.21). The subgroup of adults with DM and/or CVD showed in general the lowest prevalence ratios for all analyzed outcomes than the subgroups with the presence of obesity alone or combined (**Table 3**).

Among elders, obesity without DM and CVD was independently associated with a higher prevalence of ICU admission (PR 1.40, 95%CI 1.07-1.82) and death (PR 1.67, 95%CI 1.00-2.80). It is worth to

For peer review only

183 note that the association with death was borderline statistically significant. To a lesser extent, obesity
184 with DM and/or CVD was also associated with an increased prevalence of invasive mechanical
185 ventilation need (PR 1.66, 95%CI 1.22-2.27), ICU admission (PR 1.37, 95%CI 1.19-1.59), and death
186 (PR 1.39, 95%CI 1.07-1.80). Elders with DM and/or CVD had the lowest prevalence ratios for the
187 analyzed outcomes than the subgroups of elders with obesity alone or combined (**Table 3**).

188 In the analyses by the degree of obesity, we did not observe much difference in the prevalence of
189 adverse outcomes, except for the prevalence of death that increased with the severity of obesity: Class
190 I 1.32 (95%CI 1.05-1.66), Class II 1.41 (1.06-1.87), and Class III 1.77 (1.35-2.33) (**Table 4**).

191 The sensitivity analysis, excluding the cases of chronic pulmonary diseases and immunosuppression
192 (**Supplementary Tables 2-3**), showed no difference in the results when compared with the estimates
193 described above which were instead adjusted for these comorbidities. Only small differences in the
194 magnitude of the associations, but not in the direction and significance, were observed.

195 Discussion

196 This is the first study that describe the relationship of obesity and COVID-19 in Brazil, based on a
197 large nationwide sample of adults and elders tested positive for SARS-CoV-2 and admitted to public
198 and private hospitals. Our results highlights that obesity with DM and/or CVD was associated with
199 higher rates of invasive mechanical ventilation use, ICU admission, and death in adults, while obesity
200 alone (without DM and CVD) was associated with higher rates of ICU admission and death among
201 elders. In both age groups, obesity alone and obesity combined with DM and/or CVD had more
202 impact on the risk of all severe COVID-19 outcomes than the subgroup with DM and/or CVD. The
203 study also supports the independent association of obesity with the analyzed outcomes and a dose-
204 response association between degrees of obesity and death in adults.

205 Some mechanisms related to the role of obesity and related diseases in worsening the clinical
206 condition of patients affected by SARS-CoV-2 have been pointed out: i) greater body weight causes
207 less elasticity of the chest wall and less total compliance of the respiratory system, leading to a

For peer review only

restriction of the ventilation and the excursion of the diaphragm, making difficult the airway management in patients with obesity;[28] ii) obesity is associated with sleep apnea syndrome and chronic obstructive pulmonary disease, which lead to surfactant dysfunction and impede the proper functioning of the airways;[29] iii) obesity is a metabolic and inflammatory disease, which is associated with the development or worsening of other chronic and endocrine comorbidities (e.g. type 2 diabetes, hypertension, dyslipidemia and CVD) that can modify innate and adaptive immune responses, making the immune system more vulnerable to infections and less responsive to antivirals and antimicrobial drugs;[16] iv) glycemic decompensation, common in patients with obesity, is associated with impaired ventilation function.[29]

It is important to note that the COVID-19 pandemic imposes a double burden of disease, especially among the elderly individuals, since the prevalence of diabetes, hypertension, cardiovascular diseases, and other comorbidities associated with COVID-19 severity increases with age.[3,30] However, our study suggests that obesity combined with diabetes and/or cardiovascular disease may offer higher risk of COVID-19 severity for adults although the overall prevalence of diseases and rates of ICU admission and mortality were higher in elders. Obesity alone seemed to provide higher risk of severe outcomes, especially death, in elders.

Few studies to-date have explored the combined and additional effect of obesity on COVID-19 severity.[13,31] A study investigated the patterns of multimorbidity among fatal cases of COVID-19 in Colombia.[31] Similar to our study, the authors found that obesity alone or with other diseases was associated with a higher risk of COVID-19 fatality among young people. Furthermore, a population-based study in Mexico observed that the addition of obesity to any number of comorbidities significantly increased the risk of COVID-19 lethality.[13] Using a causally ordered mediation analysis, this study also found that 49.5% of the effect of diabetes on COVID-19 lethality was mediated by obesity, particularly in early-onset cases < 40 years of age.

Other studies also suggest that obesity is independently associated with severe outcomes of COVID-19, regardless of age and other associated comorbidities.[11-14] A large study in Mexico[13] showed that

For peer review only

patients with obesity had higher rates of ICU admission and were more likely to be intubated in relation to patients without obesity. This study also found a five-fold increased risk of mortality due to COVID-19 in patients with obesity.[13] In a hospital-based study in France, it was observed that BMI > 35 kg/m^2 was associated with the need for invasive mechanical ventilation.[14]

Few studies to-date have similarly found a dose-response association between degrees of obesity and COVID-19 death.[32] Based on care records of 17,278,392 UK adults, the study showed that the risk of COVID-19 death increases independently with the degree of obesity: 30-34.9 kg/m^2 (HR 1.05), 35-39.9 kg/m^2 (1.40), and ≥40 kg/m^2 (2.66).[32] Other studies have evidenced the association of obesity with COVID-19 complications and death among adults.[12,33] A hospital-based study in New York City showed that morbid obesity (BMI ≥ 40 kg/m^2) is strongly and independently associated with death in hospitalized patients younger than 50 years.[33] Another study in New York City found a similar dose-response relationship between degrees of obesity and acute and critical care.[12] Patients less than 60 years old with BMI between 30 and 34.9 kg/m^2 (obesity class I) were 2.0 and 1.8 times more likely to be respectively admitted for acute care (general hospital admission) and critical care (ICU admission or invasive ventilator) compared to individuals with BMI < 30 kg/m^2. Patients of the same age group with BMI ≥ 35 kg/m^2 (obesity class II and III) showed 2.2 and 3.6 more chances of being hospitalized for acute and critical care, respectively.[12]

Strengths and limitations

One of the greatest strengths of the study was the use of SIVEP-Gripe dataset. Because severe acute respiratory syndrome is a condition of compulsory notification in both public and private hospitals,[34] we have a nationwide representative sample of patients hospitalized for severe COVID-19 in Brazil. In addition, the large sample sizes allowed us to analyze adults and elders separately, as well as the degrees of obesity which dose-response association with death was evidenced. The availability of important confounding variables (sex, age, and preexisting comorbidities) to control the estimated associations, as well as hospital outcomes and mortality of COVID-19, was another differential of the study. Only patients with positive RT-PCR test for SARS-CoV-2 and final diagnosis for COVID-

For peer review only

19 were included which gives greater precision on the studied population. The availability and use of data from health surveillance systems may be a lesson from Brazil that other countries can learn for obtaining routine and timely data to guide health systems and research in preparing and responding to pandemics before and during their course.

The study also has some limitations that must be considered. Because this is a cross-sectional study, a causal association cannot be inferred. As we used routinely collected data, which has not been designed primarily for research purposes, it may bring well-known limitations related to missing, underestimation, and potential misclassification. Obesity prevalence may have been underestimated due to the completeness of obesity and BMI data. Previous studies using SIVEP-Gripe data have also found a low prevalence of obesity in this population.[35,36] Better routine collection of height and weight data is still needed in clinical practice. Also, we believe that health professionals have adopted more the one method to collect weight and height information for BMI calculation, such as the patient's self-report and direct measure. Therefore, in addition to BMI which implausible values were checked and excluded, the classification of obesity was also confirmed from a dichotomous variable on the presence of obesity (no/yes). Although it is known that BMI does not distinguish between fat and lean body mass, and thus may lead to misclassification bias, BMI has been shown as a strong predictor of excess body fat and has been widely used in epidemiological studies.[15] Information for some outcomes were incomplete because some patients were still hospitalized on the study endpoint date. However, that did not represent a potential selection bias to our study. The analytical samples in each outcome had similar demographic and clinical characteristics than the overall study population and the excluded samples (Supplementary Table 1). Data on ethnicity/race was very incomplete, and thus was not included in the analysis. Additional studies are needed to further explore the relationship between socioeconomic characteristics and obesity in severe disease. Finally, the generalization of results must be cautious since the study included only hospitalized cases of COVID-19.

Conclusions

The combined association of obesity, diabetes, and/or cardiovascular disease with severe COVID-19 outcomes, especially ICU admission and death, may be stronger in adult than in elderly inpatients. In both age groups, obesity alone and obesity combined with DM and/or CVD had more impact on the risk of all severe COVID-19 outcomes than the subgroup with DM and/or CVD. The study also supports an independent relationship of obesity with the severe outcomes, including a dose-response association between degrees of obesity and death in adults. These findings suggest important implications for the clinical care of patients with obesity and severe COVID-19, such as the increased need of critical care and higher risk of death among these patients. Our study also supports the inclusion of people with obesity, independently of other preexisting comorbidities and age, in the high-risk and vaccine priority groups for protection from SARS-CoV-2 infection.

Acknowledgments

The authors thank the members of Rede CoVida's Epidemiology & Information Group for the work of identifying and collecting data related to COVID-19.

Author Contributions

NJS, RCRS, and RLF designed the study and analysis strategy. NJS, CASTS, and MYTI obtained, documented, and described the data. AJFF, CSST, ASR, FJOA, and IRF carried out the literature search. NJS and EJP performed the data analysis. NJS, RCRS, AJFF, CSST, ASR, FJOA, IRF, ESP and MLB contributed to data interpretation. NJS, AJFF, CSST, ASR, FJOA, and IRF drafted the manuscript. RCRS, ESP, MYTI, and MLB critically revised the manuscript. All authors read and approved the final manuscript.

Funding

All authors are affiliated to the Centre for Data and Knowledge Integration for Health (CIDACS) that is funded and supported by MCTI/ CNPq/ MS/ SCTIE/ Decit/ Bill & Melinda Gates Foundation's GCE Brazil (OPP1142172), Wellcome Trust (202912/Z/16/Z), the Brazilian Health Surveillance Secretariat, Ministry of Health, Bahia State, Research Support Foundation of the State of Bahia (FAPESB), the

For peer review only

Research and Project Funding Agency (FINEP), and the Secretariat of Science and Technology of the State of Bahia (SECTI). Dr Paixão is a fellow supported by the Wellcome Trust (213589/Z/18/Z).

Competing Interests

None declared.

Patient Consent for Publication

Not required.

Data Availability Statement

Data is freely available without restriction at https://opendatasus.saude.gov.br/dataset/bd-srag-2020.

Code book and analytic code will be made available upon request from the corresponding author.

Ethics Statement

The study was conducted according to the guidelines laid down in the Declaration of Helsinki. As the study exclusively used publicly available de-identified data, ethics approval by a research ethics committee and informed consent are waived per Resolution n. 466/2012 of the National Health Council of Brazil's Commission of Ethics in Research.

For peer review only

References

1. World Health Organization. WHO Coronavirus Disease (COVID-19) Dashboard. https://covid19.who.int/. Accessed May 13, 2020.
2. Centers for Disease Control and Prevention. Coronavirus Disease 2019 (COVID-19). People Who Are at Increased Risk for Severe Illness. https://www.cdc.gov/coronavirus/2019-ncov/need-extra-precautions/people-at-increased-risk.html. Accessed July 21, 2020.
3. Liu H, Chen S, Liu M, Nie H, Lu H. Comorbid Chronic Diseases are Strongly Correlated with Disease Severity among COVID-19 Patients: A Systematic Review and Meta-Analysis. *Aging Dis*. 2020;11(3):668-678. doi:10.14336/ad.2020.0502.
4. Alberca RW, Oliveira LM, Branco ACCC, Pereira NZ, Sato MN. Obesity as a risk factor for COVID-19: an overview [published online June 15, 2020]. *Crit Rev Food Sci Nutr*. doi:10.1080/10408398.2020.1775546.
5. Sattar N, McInnes IB, McMurray JJV. Obesity Is a Risk Factor for Severe COVID-19 Infection: Multiple Potential Mechanisms. *Circulation*. 2020;142(1):4-6. doi:10.1161/circulationaha.120.047659.
6. Caussy C, Wallet F, Laville M, Disse E. Obesity is Associated with Severe Forms of COVID-19. *Obesity (Silver Spring)*. 2020;28(7):1175. doi:10.1002/oby.22842.
7. Li Q, Guan X, Wu P, et al. Early Transmission Dynamics in Wuhan, China, of Novel Coronavirus-Infected Pneumonia. *N Engl J Med*. 2020;382(13):1199-1207. doi:10.1056/nejmoa2001316.
8. Grasselli G, Zangrillo A, Zanella A, et al. Baseline Characteristics and Outcomes of 1591 Patients Infected with SARS-CoV-2 Admitted to ICUs of the Lombardy Region, Italy. *JAMA*. 2020;323(16):1574-1581. doi:10.1001/jama.2020.5394.
9. Caussy C, Pattou F, Wallet F, et al. Prevalence of obesity among adult inpatients with COVID-19 in France. *Lancet Diabetes Endocrinol*. 2020;8(7):562-564. doi:10.1016/s2213-8587(20)30160-1.
10. Hernández-Garduño E. Obesity is the comorbidity more strongly associated for Covid-19 in Mexico. A case-control study. *Obes Res Clin Pract*. 2020;14(4):375-379. doi:10.1016/j.orcp.2020.06.001.
11. Hajifathalian K, Kumar S, Newberry C, et al. Obesity is associated with worse outcomes in COVID-19: Analysis of Early Data From New York City. *Obesity (Silver Spring)*. 2020;28(9):1606-1612. doi:10.1002/oby.22923.
12. Lighter J, Phillips M, Hochman S, et al. Obesity in patients younger than 60 years is a risk factor for Covid-19 hospital admission. *Clin Infect Dis*. 2020;71(15):896-897. doi:10.1093/cid/ciaa415.
13. Bello-Chavolla OY, Bahena-López JP, Antonio-Villa NE, et al. Predicting Mortality Due to SARS-CoV-2: A Mechanistic Score Relating Obesity and Diabetes to COVID-19 Outcomes in Mexico. *J Clin Endocrinol Metab*. 2020;105(8):dgaa346. doi:10.1210/clinem/dgaa346.
14. Simonnet A, Chetboun M, Poissy J, et al. High Prevalence of Obesity in Severe Acute Respiratory Syndrome Coronavirus-2 (SARS-CoV-2) Requiring Invasive Mechanical Ventilation. *Obesity (Silver Spring)*. 2020;28(7):1195-1199. doi:10.1002/oby.22831.
15. NCD Risk Factor Collaboration (NCD-RisC). Worldwide trends in body-mass index, underweight, overweight, and obesity from 1975 to 2016: a pooled analysis of 2416 population-based measurement studies in 128.9 million children, adolescents, and adults. *Lancet*. 2017;390(10113):2627-2642. doi:10.1016/s0140-6736(17)32129-3.
16. GBD 2015 Obesity Collaborators. Health Effects of Overweight and Obesity in 195 Countries over 25 Years. *N Engl J Med*. 2017;377(1):13-27. doi:10.1056/nejmoa1614362.
17. Brasil. Ministério da Saúde. Vigitel Brasil 2018: vigilância de fatores de risco e proteção para doenças crônicas por inquérito telefônico. Brasília, DF: Ministério da Saúde; 2019. https://portalarquivos2.saude.gov.br/images/pdf/2019/julho/25/vigitel-brasil-2018.pdf. Accessed July 10, 2020.

For peer review only - http://bmjopen.bmj.com/site/about/guidelines.xhtml

18. GBD 2016 Brazil Collaborators. Burden of disease in Brazil, 1990-2016: a systematic subnational analysis for the Global Burden of Disease Study 2016. *Lancet*. 2018;392(10149):760-775. doi:10.1016/s0140-6736(18)31221-2.
19. Flint SW, Tahrani AA. COVID-19 and obesity-lack of clarity, guidance, and implications for care. *Lancet Diabetes Endocrinol*. 2020;8(6):474-475. doi:10.1016/s2213-8587(20)30156-x.
20. Brasil. Ministério da Saúde. Severe Acute Respiratory Syndrome dataset - including COVID-19 data. 2020. https://opendatasus.saude.gov.br/dataset/bd-srag-2020. Accessed July 11, 2020.
21. World Health Organization. Physical status: the use and interpretation of anthropometry. Geneva: WHO; 1995. https://apps.who.int/iris/handle/10665/37003. Accessed July 11, 2020.
22. Organización Panamericana de la Salud. División de Promoción y Protección de la Salud (HPP). Encuesta sobre Salud, Bienestar y Envejecimiento (SABE) en América Latina el Caribe: Informe Preliminar (Internet). In: XXXVI Reunión del Comité asesor de investigaciones em Salud; 9-11 jun 2001; Kingston: OPAS, 2002. http://envejecimiento.csic.es/documentos/documentos/paho-salud-01.pdf. Accessed July 11, 2020.
23. Brasil. Ministério da Saúde. Guidelines for the Collection and Analysis of Anthropometric Data in Health Services. Technical Standard for the Food and Nutrition Surveillance System. Brasília, DF: Ministério da Saúde; 2011.
24. Cheng FW, Gao X, Mitchell DC, et al. Body mass index and all-cause mortality among older adults. *Obesity (Silver Spring)*. 2016;24(10):2232-39. doi:10.1002/oby.21612.
25. Bruce N, Pope D, Stanistreet D. Quantitative methods for health research: a practical interactive guide to epidemiology and statistics (Second ed.). Hoboken, NJ. p. 16. ISBN 978-1-118-66526-8. OCLC 992438133.
26. VanderWeele TJ. Principles of confounder selection. Eur J Epidemiol. 2019;34(3):211-219. doi: 10.1007/s10654-019-00494-6.
27. Popkin BM, Du S, Green WD, et al. Individuals with obesity and COVID-19: A global perspective on the epidemiology and biological relationships. Obes Rev. 2020;21(11):e13128. doi: 10.1111/obr.13128.
28. Honce R, Schultz-Cherry S. Impact of Obesity on Influenza A Virus Pathogenesis, Immune Response, and Evolution. *Front Immunol*. 2019;10:1071. doi:10.3389/fimmu.2019.01071.
29. Puig-Domingo M, Marazuela M, Giustina A. COVID-19 and endocrine diseases. A statement from the European Society of Endocrinology. *Endocrine*. 2020;68(1):2-5. doi:10.1007/s12020-020-02294-5.
30. Docherty AB, Harrison EM, Green CA, et al. Features of 20 133 UK patients in hospital with covid-19 using the ISARIC WHO Clinical Characterisation Protocol: prospective observational cohort study. *BMJ*. 2020;369:m1985. doi:10.1136/bmj.m1985.
31. Fernández-Niño JA, Guerra-Gómez JA, Idrovo AJ. Multimorbidity patterns among COVID-19 deaths: proposal for the construction of etiological models. *Rev Panam Salud Publica*. 2020;44:e166. doi:10.26633/RPSP.2020.166.
32. Williamson EJ, Walker AJ, Bhaskaran K, et al. Factors associated with COVID-19-related death using OpenSAFELY. *Nature*. 2020;584(7821):430-436. doi:10.1038/s41586-020-2521-4.
33. Klang E, Kassim G, Soffer S, et al. Morbid Obesity as an Independent Risk Factor for COVID-19 Mortality in Hospitalized Patients Younger than 50. *Obesity (Silver Spring)*. 2020;28(9):1595-1599. doi:10.1002/oby.22913.
34. Cantarino L, Merchan-Hamann E. Influenza in Brazil: surveillance pathways. *J Infect Dev Ctries*. 2016;10(1):13-23. doi:10.3855/jidc.7135.
35. de Souza WM, Buss LF, Candido DDS, et al. Epidemiological and clinical characteristics of the COVID-19 epidemic in Brazil. *Nat Hum Behav*. 2020;4(8):856-865. doi:10.1038/s41562-020-0928-4.
36. Baqui P, Bica I, Marra V, Ercole A, van der Schaar M. Ethnic and regional variations in hospital mortality from COVID-19 in Brazil: a cross-sectional observational study. *Lancet Glob Health*. 2020;8(8):e1018-e1026. doi: 10.1016/S2214-109X(20)30285-0.

For peer review only - http://bmjopen.bmj.com/site/about/guidelines.xhtml

Figure legends

Figure 1. Selection of the study population from SIVEP-Gripe.

SIVEP-Gripe: Influenza Epidemiological Surveillance Information System.

For peer review only

Table 1. Demographic characteristics, comorbidities, hospitalization outcomes and death according to the combined exposure of obesity, diabetes, and/or cardiovascular diseases in adults with severe COVID-19.

	Total		None		OB		OB + DM and/or CVD		DM and/or CVD	
	n	%	n	%	n	%	n	%	n	%
Overall	8848	100.0	3161	35.7	409	4.6	452	5.1	4826	54.6
**Sex**										
Female	3774	42.7	1511	40.0	165	4.4	199	5.3	1899	50.3
Male	5074	57.4	1650	32.5	244	4.8	253	5.0	2927	57.7
**Age**										
< 40 years	1976	22.3	1064	53.9	188	9.5	102	5.2	622	31.5
≥ 40 years	6872	77.7	2097	30.5	221	3.2	350	5.1	4204	61.2
**Chronic pulmonary disease**										
No	8502	96.1	2969	34.9	388	4.6	435	5.1	4710	55.4
Yes	346	3.9	192	55.5	21	6.1	17	4.9	116	33.5
**Asthma**										
No	8184	92.5	2728	33.3	383	4.7	414	5.1	4659	56.9
Yes	664	7.5	433	65.2	26	3.9	38	5.7	167	25.2
**Chronic kidney disease**										
No	8297	93.8	2958	35.7	399	4.8	434	5.2	4506	54.3
Yes	551	6.2	203	36.8	10	1.8	18	3.3	320	58.1
**Chronic hematologic disease**										
No	8710	98.4	3081	35.4	406	4.7	445	5.1	4778	54.9
Yes	138	1.6	80	58.0	3	2.2	7	5.1	48	34.8
**Chronic neurological disease**										
No	8588	97.1	3014	35.1	406	4.7	442	5.2	4726	55.0
Yes	260	2.9	147	56.5	3	1.2	10	3.9	100	38.5
**Chronic liver disease**										
No	8684	98.2	3083	35.5	406	4.7	443	5.1	4752	54.7
Yes	164	1.9	78	47.6	3	1.8	9	5.5	74	45.1
**Immunosuppression**										
No	8276	93.5	2777	33.6	393	4.8	440	5.3	4666	56.4
Yes	572	6.5	384	67.1	16	2.8	12	2.1	160	28.0
**Mechanical ventilation***										
No	2727	33.8	1144	42.0	93	3.4	88	3.2	1402	51.4
Non-invasive	3634	45.0	1178	32.4	192	5.3	190	5.2	2074	57.1
Invasive	1714	21.2	529	30.9	101	5.9	150	8.8	934	54.5
**ICU admission***										
No	5438	64.6	2025	37.2	235	4.3	222	4.1	2956	54.4
Yes	2976	35.4	1007	33.8	163	5.5	216	7.3	1590	53.4
**Death***										
No	4525	68.9	1699	37.6	211	4.7	200	4.4	2415	53.4
Yes	2040	31.1	640	31.4	92	4.5	140	6.9	1168	57.3

OB: obesity (BMI≥30 kg/m^2), DM: diabetes mellitus, CVD: cardiovascular disease, ICU: intensive care unit.
* Mechanical ventilation (n=8075), ICU admission (n=8414), and death (n=6565).

For peer review only

Table 2. Demographic characteristics, comorbidities, hospitalization outcomes and death according to the combined exposure of obesity, diabetes, and/or cardiovascular diseases in elders with severe COVID-19.

	**Total**		**None**		**OB**		**OB + DM and/or CVD**		**DM and/or CVD**	
	**n**	**%**	**n**	**%**	**n**	**%**	**n**	**%**	**n**	**%**
Overall	12925	100.0	2837	21.9	91	0.7	358	2.8	9639	74.6
**Sex**										
Female	5968	46.2	1232	20.6	52	0.9	209	3.5	4475	75.0
Male	6957	53.8	1605	23.1	39	0.6	149	2.1	5164	74.2
**Age**										
< 80 years	9355	72.4	2011	21.5	77	0.8	309	3.3	6958	74.4
≥ 80 years	3570	27.6	826	23.1	14	0.4	49	1.4	2681	75.1
**Chronic pulmonary disease**										
No	11885	92.0	2494	21.0	85	0.7	325	2.7	8981	75.6
Yes	1040	8.1	343	33.0	6	0.6	33	3.2	658	63.3
**Asthma**										
No	12474	96.5	2687	21.5	90	0.7	336	2.7	9361	75.0
Yes	451	3.5	150	33.3	1	0.2	22	4.9	278	61.6
**Chronic kidney disease**										
No	11882	91.9	2608	22.0	85	0.7	311	2.6	8878	74.7
Yes	1043	8.1	229	22.0	6	0.6	47	4.5	761	73.0
**Chronic hematologic disease**										
No	12728	98.5	2751	21.6	91	0.7	354	2.8	9532	74.9
Yes	197	1.5	86	43.7	0	0.0	4	2.0	107	54.3
**Chronic neurological disease**										
No	11871	91.9	2511	21.2	89	0.8	338	2.9	8933	75.3
Yes	1054	8.2	326	30.9	2	0.2	20	1.9	706	67.0
**Chronic liver disease**										
No	12734	98.5	2777	21.8	87	0.7	353	2.8	9517	74.7
Yes	191	1.5	60	31.4	4	2.1	5	2.6	122	63.9
**Immunosuppression**										
No	12303	95.2	2558	20.8	87	0.7	342	2.8	9316	75.7
Yes	622	4.8	279	44.9	4	0.6	16	2.6	323	51.9
**Mechanical ventilation***										
No	2725	23.0	626	23.0	18	0.7	70	2.6	2011	73.8
Non-invasive	5557	47.0	1164	21.0	38	0.7	141	2.5	4214	75.8
Invasive	3547	30.0	767	21.6	29	0.8	133	3.8	2618	73.8
**ICU admission***										
No	6898	56.4	1578	22.9	41	0.6	168	2.4	5111	74.1
Yes	5324	43.6	1107	20.8	44	0.8	181	3.4	3992	75.0
**Death***										
No	3684	37.1	823	22.3	21	0.6	95	2.6	2745	74.5
Yes	6259	63.0	1407	22.5	43	0.7	173	2.8	4636	74.1

OB: obesity (BMI≥30 kg/m^2), DM: diabetes mellitus, CVD: cardiovascular disease, ICU: intensive care unit.
* Mechanical ventilation (n=11829), ICU admission (n=12222), and death (n=9943).

For peer review only

Table 3. Combined association of obesity, diabetes, and/or cardiovascular disease with non-invasive and invasive mechanical ventilation use, intensive care unit admission, and death in adult and elderly patients hospitalized with severe COVID-19.

	Main exposure variable	Non-invasive mechanical ventilation*				Invasive mechanical ventilation*			
		Crude model		Adjusted model #		Crude model		Adjusted model #	
		PR	95%CI	PR	95%CI	PR	95%CI	PR	95%CI
	None	1.00		1.00		1.00		1.00	
**Adults**	OB	2.00	1.54-2.60	2.13	1.64-2.78	2.35	1.74-3.17	2.69	1.98-3.65
20-59 years	OB + DM and/or CVD	2.10	1.61-2.73	2.06	1.58-2.69	3.69	2.78-4.89	3.76	2.82-5.01
	DM and/or CVD	1.44	1.29-1.60	1.35	1.20-1.51	1.44	1.26-1.64	1.32	1.14-1.52
	None	1.00		1.00		1.00		1.00	
**Elders**	OB	1.14	0.64-2.01	1.22	0.69-2.16	1.31	0.72-2.39	1.43	0.78-2.61
≥ 60 years	OB + DM and/or CVD	1.08	0.80-1.47	1.15	0.84-1.55	1.55	1.14-2.11	1.66	1.22-2.27
	DM and/or CVD	1.13	1.01-1.26	1.14	1.01-1.27	1.06	0.94-1.20	1.10	0.97-1.24
		**ICU admission****				**Death*****			
		Crude model		Adjusted model #		Crude model		Adjusted model #	
		PR	95%CI	PR	95%CI	PR	95%CI	PR	95%CI
	None	1.00		1.00		1.00		1.00	
**Adults**	OB	1.23	1.08-1.40	1.31	1.13-1.53	1.11	0.92-1.33	1.33	1.05-1.69
20-59 years	OB + DM and/or CVD	1.48	1.33-1.65	1.60	1.40-1.83	1.50	1.30-1.74	1.79	1.45-2.21
	DM and/or CVD	1.05	0.99-1.12	1.03	0.95-1.12	1.19	1.10-1.29	1.16	1.03-1.30
	None	1.00		1.00		1.00		1.00	
**Elders**	OB	1.26	1.02-1.55	1.40	1.07-1.82	1.06	0.89-1.27	1.67	1.00-2.80
≥ 60 years	OB + DM and/or CVD	1.26	1.13-1.41	1.37	1.19-1.59	1.02	0.93-1.12	1.39	1.07-1.80
	DM and/or CVD	1.06	1.01-1.12	1.11	1.04-1.18	1.00	0.96-1.03	1.05	0.95-1.16

OB: obesity (BMI≥30 kg/m^2), DM: diabetes mellitus, CVD: cardiovascular disease, ICU: intensive care unit, PR: prevalence ratio, 95%CI: 95% confidence interval.
* Crude and adjusted multinomial logistic regression models for mechanical ventilation use in adults (n=8075) and elders (n=11829).
** Crude and adjusted logistic regression models for ICU admission in adults (n= 8414) and elders (n=12222).
*** Crude and adjusted logistic regression models for death in adults (n=6565) and elders (n=9943).
# Adjusted for sex, age in years, pulmonary disease, asthma, kidney disease, hematologic disease, neurological disease, liver disease, and immunosuppression.

For peer review only

Table 4. Independent association of degrees of obesity with non-invasive and invasive mechanical ventilation, intensive care unit admission, and death in hospitalized adults with severe COVID-19.

Main exposure variable	Non-invasive mechanical ventilation*				Invasive mechanical ventilation*			
	Crude model		Adjusted model #		Crude model		Adjusted model #	
	PR	95%CI	PR	95%CI	PR	95%CI	PR	95%CI
No obesity (< 30 kg/m^2)	1.00		1.00		1.00		1.00	
Obesity class I (≥ 30-34.9 kg/m^2)	1.78	1.35-2.33	1.91	1.45-2.51	2.59	1.93-3.47	3.00	2.22-4.05
Obesity class II (≥ 35-39.9 kg/m^2)	1.44	1.04-2.00	1.58	1.14-2.19	2.10	1.47-2.99	2.47	1.72-3.54
Obesity class III (≥ 40 kg/m^2)	1.70	1.19-2.44	1.88	1.31-2.69	2.51	1.71-3.70	3.00	2.03-4.45
	ICU admission**				Death**			
	Crude model		Adjusted model #		Crude model		Adjusted model #	
	PR	95%CI	PR	95%CI	PR	95%CI	PR	95%CI
No obesity (< 30 kg/m^2)	1.00		1.00		1.00		1.00	
Obesity class I (≥ 30-34.9 kg/m^2)	1.31	1.17-1.47	1.42	1.23-1.64	1.11	0.94-1.31	1.32	1.05-1.66
Obesity class II (≥ 35-39.9 kg/m^2)	1.34	1.16-1.54	1.46	1.23-1.74	1.16	0.95-1.42	1.41	1.06-1.87
Obesity class III (≥ 40 kg/m^2)	1.32	1.14-1.54	1.45	1.20-1.74	1.33	1.10-1.59	1.77	1.35-2.33

Degrees of obesity defined by the WHO cutoff points.
PR: prevalence ratio, 95%CI: 95% confidence interval.
* Crude and adjusted multinomial logistic regression models for mechanical ventilation use (n=8075).
** Crude and adjusted logistic regression models for ICU admission (n=8414) and mortality (n=6565).
# Adjusted for sex, age in years, diabetes mellitus, cardiovascular disease, pulmonary disease, asthma, kidney disease, hematologic disease, neurological disease, liver disease, and immunosuppression.

For peer review only

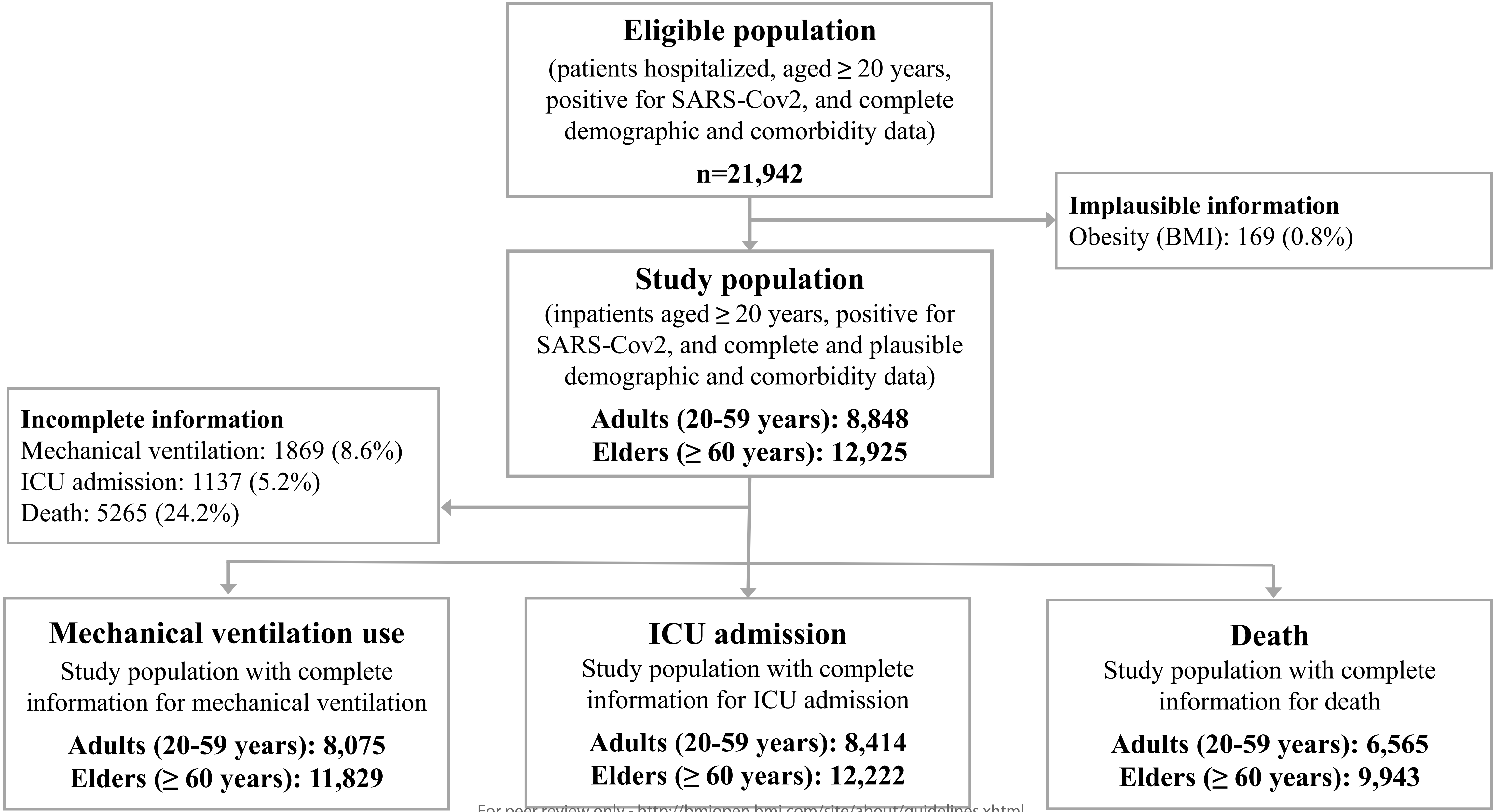

For peer review only - http://bmjopen.bmj.com/site/about/guidelines.xhtml

Suppl. Table 1. Characteristics of the study population and study samples included and excluded of the analysis for each severe COVID-19 outcome.

	Study population*		Mechanical ventilation				ICU admission				Death			
			Included		Excluded		Included		Excluded		Included		Excluded	
	n	%	n	%	n	%	n	%	n	%	n	%	n	%
Overall	21,773	100.0	19,904	91.4	1,869	8.6	20,636	94.8	1,137	5.2	16,508	75.8	5,265	24.2
**Sex**														
Female	9,742	44.7	8,905	44.7	837	44.8	9,227	44.7	515	45.3	7,379	44.7	2,363	44.9
Male	12,031	55.3	10,999	55.3	1,032	55.2	11,409	55.3	622	54.7	9,129	55.3	2,902	55.1
**Age (years)**														
20-39	1,976	9.1	1,808	9.1	168	9.0	1,890	9.2	86	7.6	1,445	8.8	531	10.1
40-59	6,872	31.6	6,267	31.5	605	32.4	6,524	31.6	348	30.6	5,120	31.0	1,752	33.3
60-79	9,355	43.0	8,546	42.9	809	43.3	8,827	42.8	528	46.4	7,062	42.8	2,293	43.6
>= 80	3,570	16.4	3,283	16.5	287	15.4	3,395	16.5	175	15.4	2,881	17.5	689	13.1
**Obesity**														
No	20,463	94.0	18,661	93.8	1,802	96.4	19,366	93.9	1,097	96.5	15,533	94.1	4,930	93.6
Yes	1,310	6.0	1,243	6.2	67	3.6	1,270	6.2	40	3.5	975	5.9	335	6.4
**Diabetes**														
No	13,058	60.0	11,925	59.9	1,133	60.6	12,401	60.1	657	57.8	9,925	60.1	3,133	59.5
Yes	8,715	40.0	7,979	40.1	736	39.4	8,235	39.9	480	42.2	6,583	39.9	2,132	40.5
**Cardiovascular disease**														
No	10,391	47.7	9,400	47.2	991	53.0	9,858	47.8	533	46.9	7,866	47.7	2,525	48.0
Yes	11,382	52.3	10,504	52.8	878	47.0	10,778	52.2	604	53.1	8,642	52.4	2,740	52.0
**Chronic pulmonary disease**														
No	20,387	93.6	18,630	93.6	1,757	94.0	19,306	93.6	1,081	95.1	15,422	93.4	4,965	94.3
Yes	1,386	6.4	1,274	6.4	112	6.0	1,330	6.5	56	4.9	1,086	6.6	300	5.7
**Asthma**														
No	20,658	94.9	18,897	94.9	1,761	94.2	19,571	94.8	1,087	95.6	15,666	94.9	4,992	94.8
Yes	1,115	5.1	1,007	5.1	108	5.8	1,065	5.2	50	4.4	842	5.1	273	5.2
**Chronic kidney disease**														
No	20,179	92.7	18,420	92.5	1,759	94.1	19,106	92.6	1,073	94.4	15,255	92.4	4,924	93.5
Yes	1,594	7.3	1,484	7.5	110	5.9	1,530	7.4	64	5.6	1,253	7.6	341	6.5
**Chronic hematologic disease**														
No	21,438	98.5	19,597	98.5	1,841	98.5	20,313	98.4	1,125	98.9	16,245	98.4	5,193	98.6
Yes	335	1.5	307	1.5	28	1.5	323	1.6	12	1.1	263	1.6	72	1.4
**Chronic neurological disease**														
No	20,459	94.0	18,700	94.0	1,759	94.1	19,377	93.9	1,082	95.2	15,460	93.7	4,999	95.0
Yes	1,314	6.0	1,204	6.1	110	5.9	1,259	6.1	55	4.8	1,048	6.4	266	5.1

For peer review only

**Chronic liver disease**																
No	21,418	98.4	19,581	98.4	1,837	98.3	20,300	98.4	1,118	98.3	16,218	98.2	5,200	98.8
Yes	355	1.6	323	1.6	32	1.7	336	1.6	19	1.7	290	1.8	65	1.2
**Immunosuppression**														
No	20,579	94.5	18,792	94.4	1,787	95.6	19,485	94.4	1,094	96.2	15,571	94.3	5,008	95.1
Yes	1,194	5.5	1,112	5.6	82	4.4	1,151	5.6	43	3.8	937	5.7	257	4.9

For peer review only

Suppl. Table 2. Combined association of obesity, diabetes, and/or cardiovascular disease with death in adult and elderly patients hospitalized with severe COVID-19, excluding the cases of chronic pulmonary diseases and immunosuppression.

	**Main exposure variable**	**Death***	
		PR	95%CI
	None	1.00	
**Adults** 20-59 years	OB	1.38	1.07-1.79
	OB + DM and/or CVD	1.86	1.49-2.32
	DM and/or CVD	1.16	1.03-1.32
	None	1.00	
**Elders** ≥ 60 years	OB	1.64	0.99-2.82
	OB + DM and/or CVD	1.43	1.08-1.90
	DM and/or CVD	1.05	0.94-1.17

OB: obesity (BMI≥30 kg/m^2), DM: diabetes mellitus, CVD: cardiovascular disease, ICU: intensive care unit, PR: prevalence ratio, 95%CI: 95% confidence interval.
[#] Adjusted for sex, age in years, asthma, kidney disease, hematologic disease, neurological disease, and liver disease.
* Adjusted logistic regression model for death in adults (n=5889) and elders (n=8676).

For peer review only

Suppl. Table 3. Independent association of degrees of obesity with death in hospitalized adults with severe COVID-19, excluding the cases of chronic pulmonary disease and immunosuppression.

Main exposure variable	Death*	
	PR	95%CI
No obesity (< 30 kg/m^2)	1.00	
Obesity class I (≥ 30-34.9 kg/m^2)	1.39	1.09-1.77
Obesity class II (≥ 35-39.9 kg/m^2)	1.48	1.10-1.99
Obesity class III (≥ 40 kg/m^2)	1.79	1.34-2.41

Degrees of obesity defined by the WHO cutoff points.
PR: prevalence ratio, 95%CI: 95% confidence interval.
* Adjusted logistic regression model for death (n=5889).
[#] Adjusted for sex, age in years, diabetes mellitus, cardiovascular disease, asthma, kidney disease, hematologic disease, neurological disease, and liver disease.

For peer review only

The RECORD statement – checklist of items, extended from the STROBE statement, that should be reported in observational studies using routinely collected health data.

	Item No.	STROBE items	Location in manuscript where items are reported	RECORD items	Location in manuscript where items are reported
**Title and abstract**					
	1	(a) Indicate the study's design with a commonly used term in the title or the abstract (b) Provide in the abstract an informative and balanced summary of what was done and what was found		RECORD 1.1: The type of data used should be specified in the title or abstract. When possible, the name of the databases used should be included.<br><br>RECORD 1.2: If applicable, the geographic region and timeframe within which the study took place should be reported in the title or abstract.<br><br>RECORD 1.3: If linkage between databases was conducted for the study, this should be clearly stated in the title or abstract.	Page 1-2<br><br>Page 1-2<br><br>N/A
**Introduction**					
Background rationale	2	Explain the scientific background and rationale for the investigation being reported			Pages 4-5
Objectives	3	State specific objectives, including any prespecified hypotheses			Pages 4-5
**Methods**					
Study Design	4	Present key elements of study design early in the paper			Page 5
Setting	5	Describe the setting, locations, and relevant dates, including periods of recruitment, exposure, follow-up, and data collection			Page 5

For peer review only

Participants	6	*(a) Cohort study* - Give the eligibility criteria, and the sources and methods of selection of participants. Describe methods of follow-up<br>*Case-control study* - Give the eligibility criteria, and the sources and methods of case ascertainment and control selection. Give the rationale for the choice of cases and controls<br>*Cross-sectional study* - Give the eligibility criteria, and the sources and methods of selection of participants<br><br>*(b) Cohort study* - For matched studies, give matching criteria and number of exposed and unexposed<br>*Case-control study* - For matched studies, give matching criteria and the number of controls per case		RECORD 6.1: The methods of study population selection (such as codes or algorithms used to identify subjects) should be listed in detail. If this is not possible, an explanation should be provided.<br><br>RECORD 6.2: Any validation studies of the codes or algorithms used to select the population should be referenced. If validation was conducted for this study and not published elsewhere, detailed methods and results should be provided.<br><br>RECORD 6.3: If the study involved linkage of databases, consider use of a flow diagram or other graphical display to demonstrate the data linkage process, including the number of individuals with linked data at each stage.	Page 5
Variables	7	Clearly define all outcomes, exposures, predictors, potential confounders, and effect modifiers. Give diagnostic criteria, if applicable.		RECORD 7.1: A complete list of codes and algorithms used to classify exposures, outcomes, confounders, and effect modifiers should be provided. If these cannot be reported, an explanation should be provided.	Pages 5-6
Data sources/ measurement	8	For each variable of interest, give sources of data and details of methods of assessment (measurement).<br>Describe comparability of assessment methods if there is more than one group			Pages 5-6

For peer review only

Bias	9	Describe any efforts to address potential sources of bias			Pages 11-12
Study size	10	Explain how the study size was arrived at			Page 5
Quantitative variables	11	Explain how quantitative variables were handled in the analyses. If applicable, describe which groupings were chosen, and why			Pages 5-6
Statistical methods	12	(a) Describe all statistical methods, including those used to control for confounding<br>(b) Describe any methods used to examine subgroups and interactions<br>(c) Explain how missing data were addressed<br>(d) *Cohort study* - If applicable, explain how loss to follow-up was addressed<br>*Case-control study* - If applicable, explain how matching of cases and controls was addressed<br>*Cross-sectional study* - If applicable, describe analytical methods taking account of sampling strategy<br>(e) Describe any sensitivity analyses			Pages 6-7
Data access and cleaning methods		..		RECORD 12.1: Authors should describe the extent to which the investigators had access to the database population used to create the study population.	Page 5

For peer review only

				RECORD 12.2: Authors should provide information on the data cleaning methods used in the study.	
Linkage		..		RECORD 12.3: State whether the study included person-level, institutional-level, or other data linkage across two or more databases. The methods of linkage and methods of linkage quality evaluation should be provided.	N/A
**Results**					
Participants	13	(a) Report the numbers of individuals at each stage of the study (*e.g.*, numbers potentially eligible, examined for eligibility, confirmed eligible, included in the study, completing follow-up, and analysed)<br>(b) Give reasons for non-participation at each stage.<br>(c) Consider use of a flow diagram		RECORD 13.1: Describe in detail the selection of the persons included in the study (*i.e.,* study population selection) including filtering based on data quality, data availability and linkage. The selection of included persons can be described in the text and/or by means of the study flow diagram.	Page 8
Descriptive data	14	(a) Give characteristics of study participants (*e.g.*, demographic, clinical, social) and information on exposures and potential confounders<br>(b) Indicate the number of participants with missing data for each variable of interest<br>(c) *Cohort study* - summarise follow-up time (*e.g.*, average and total amount)			Page 8
Outcome data	15	*Cohort study* - Report numbers of outcome events or summary measures over time<br>*Case-control study* - Report numbers in each exposure			Page 8

For peer review only

		category, or summary measures of exposure<br>*Cross-sectional study* - Report numbers of outcome events or summary measures			
Main results	16	(a) Give unadjusted estimates and, if applicable, confounder-adjusted estimates and their precision (e.g., 95% confidence interval). Make clear which confounders were adjusted for and why they were included<br>(b) Report category boundaries when continuous variables were categorized<br>(c) If relevant, consider translating estimates of relative risk into absolute risk for a meaningful time period			Pages 8-9
Other analyses	17	Report other analyses done—e.g., analyses of subgroups and interactions, and sensitivity analyses			Page 9
**Discussion**					
Key results	18	Summarise key results with reference to study objectives			Page 9
Limitations	19	Discuss limitations of the study, taking into account sources of potential bias or imprecision. Discuss both direction and magnitude of any potential bias		RECORD 19.1: Discuss the implications of using data that were not created or collected to answer the specific research question(s). Include discussion of misclassification bias, unmeasured confounding, missing data, and changing eligibility over time, as they pertain to the study being reported.	Page 11-12
Interpretation	20	Give a cautious overall interpretation of results considering objectives,			Pages 9-11

		limitations, multiplicity of analyses, results from similar studies, and other relevant evidence			
Generalisability	21	Discuss the generalisability (external validity) of the study results			Page 12
**Other Information**					
Funding	22	Give the source of funding and the role of the funders for the present study and, if applicable, for the original study on which the present article is based			Page 13
Accessibility of protocol, raw data, and programming code		..		RECORD 22.1: Authors should provide information on how to access any supplemental information such as the study protocol, raw data, or programming code.	Page 14

*Reference: Benchimol EI, Smeeth L, Guttmann A, Harron K, Moher D, Petersen I, Sørensen HT, von Elm E, Langan SM, the RECORD Working Committee. The REporting of studies Conducted using Observational Routinely-collected health Data (RECORD) Statement. *PLoS Medicine* 2015; in press.

*Checklist is protected under Creative Commons Attribution (CC BY) license.

For peer review only

BMJ Open

Combined association of obesity and other cardiometabolic diseases with severe COVID-19 outcomes: a nationwide cross-sectional study of 21,773 Brazilian adult and elderly inpatients

Journal:	*BMJ Open*
Manuscript ID	bmjopen-2021-050739.R3
Article Type:	Original research
Date Submitted by the Author:	13-Jul-2021

Complete List of Authors:	Silva, Natanael; Oswaldo Cruz Foundation, Centre for Data and Knowledge Integration for Health<br>Silva, Rita de Cássia; Federal University of Bahia, School of Nutrition; Oswaldo Cruz Foundation, Centre for Data and Knowledge Integration for Health<br>Ferreira, Andrêa; Federal University of Bahia, Institute of Collective Health; Oswaldo Cruz Foundation, Centre for Data and Knowledge Integration for Health<br>Teixeira, Camila; Federal University of Bahia, Institute of Collective Health; Oswaldo Cruz Foundation, Centre for Data and Knowledge Integration for Health<br>Rocha, Aline; Federal University of Bahia, School of Nutrition; Oswaldo Cruz Foundation, Centre for Data and Knowledge Integration for Health<br>Alves, Flávia Jôse; Federal University of Bahia, Institute of Collective Health; Oswaldo Cruz Foundation, Centre for Data and Knowledge Integration for Health<br>Falcão, Ila; Oswaldo Cruz Foundation, Centre for Data and Knowledge Integration for Health<br>Pinto, Elizabete; Federal University of Reconcavo da Bahia, Center for Health Sciences; Oswaldo Cruz Foundation, Centre for Data and Knowledge Integration for Health<br>Santos, Carlos Antônio; Oswaldo Cruz Foundation, Centre for Data and Knowledge Integration for Health; State University of Feira de Santana, Department of Exact Sciences<br>Fiaccone, Rosemeire; Federal University of Bahia, Institute of Mathematics and Statistics; Oswaldo Cruz Foundation, Centre for Data and Knowledge Integration for Health<br>Ichihara, Maria Yury; Oswaldo Cruz Foundation, Centre for Data and Knowledge Integration for Health<br>Paixão, Enny; London School of Hygiene and Tropical Medicine Faculty of Epidemiology and Population Health; Oswaldo Cruz Foundation, Centre for Data and Knowledge Integration for Health<br>Barreto, Mauricio; Oswaldo Cruz Foundation, Centre for Data and Knowledge Integration for Health; Federal University of Bahia, Institute of Collective Health

**Primary Subject Heading**:	Epidemiology

Secondary Subject Heading:	Infectious diseases, Nutrition and metabolism
Keywords:	COVID-19, INFECTIOUS DISEASES, INTENSIVE & CRITICAL CARE, DIABETES & ENDOCRINOLOGY, NUTRITION & DIETETICS, EPIDEMIOLOGY

SCHOLARONE™
Manuscripts

I, the Submitting Author has the right to grant and does grant on behalf of all authors of the Work (as defined in the below author licence), an exclusive licence and/or a non-exclusive licence for contributions from authors who are: i) UK Crown employees; ii) where BMJ has agreed a CC-BY licence shall apply, and/or iii) in accordance with the terms applicable for US Federal Government officers or employees acting as part of their official duties; on a worldwide, perpetual, irrevocable, royalty-free basis to BMJ Publishing Group Ltd ("BMJ") its licensees and where the relevant Journal is co-owned by BMJ to the co-owners of the Journal, to publish the Work in this journal and any other BMJ products and to exploit all rights, as set out in our licence.

The Submitting Author accepts and understands that any supply made under these terms is made by BMJ to the Submitting Author unless you are acting as an employee on behalf of your employer or a postgraduate student of an affiliated institution which is paying any applicable article publishing charge ("APC") for Open Access articles. Where the Submitting Author wishes to make the Work available on an Open Access basis (and intends to pay the relevant APC), the terms of reuse of such Open Access shall be governed by a Creative Commons licence – details of these licences and which Creative Commons licence will apply to this Work are set out in our licence referred to above.

Other than as permitted in any relevant BMJ Author's Self Archiving Policies, I confirm this Work has not been accepted for publication elsewhere, is not being considered for publication elsewhere and does not duplicate material already published. I confirm all authors consent to publication of this Work and authorise the granting of this licence.

For peer review only

Combined association of obesity and other cardiometabolic diseases with severe COVID-19 outcomes: a nationwide cross-sectional study of 21,773 Brazilian adult and elderly inpatients

Natanael J Silva, MSc[1,2]; Rita C Ribeiro-Silva, PhD[1,2,3]; Andrêa JF Ferreira, MSc[1,2,4]; Camila SS Teixeira, MSc[1,2,4]; Aline S Rocha, MSc[1,2,3]; Flávia Jôse O Alves, MSc[1,2,4]; Ila R Falcão, PhD[2]; Elizabete J Pinto, PhD[2,5]; Carlos Antônio ST Santos, PhD[1,2,6]; Rosemeire L Fiaccone, PhD[1,2,7]; Maria Yury T Ichihara, MD PhD[1,2]; Enny S Paixão, PhD[1,2,8]; Mauricio L Barreto, MD PhD[1,2,4]

[1] Rede CoVida, Salvador, BA, Brazil

[2] Centre for Data and Knowledge Integration for Health, Oswaldo Cruz Foundation, Salvador, BA, Brazil

[3] School of Nutrition, Federal University of Bahia, Salvador, BA, Brazil

[4] Institute of Collective Health, Federal University of Bahia, Salvador, BA, Brazil

[5] Center for Health Sciences, Federal University of Recôncavo da Bahia, Santo Antônio de Jesus, BA, Brazil

[6] Department of Exact Sciences, State University of Feira de Santana, Feira de Santana, BA, Brazil

[7] Institute of Mathematics and Statistics, Federal University of Bahia, Salvador, BA, Brazil

[8] Epidemiology and Population Health, London School of Hygiene and Tropical Medicine, London, UK

Corresponding author: Natanael J Silva. Centre for Data and Knowledge Integration for Health, Oswaldo Cruz Foundation. Edf. Tecnocentro, Sl 315. Rua Mundo, 121. Trobogy, Salvador, BA, 41745-715, Brazil. Phone number: +55 79 99839-3991. Email: <silva_natanael@hotmail.com>.

Keywords: Obesity, COVID-19, SARS-CoV-2, Hospitalization, Mortality.

Word count: 3,389

For peer review only

Abstract

Objectives: To investigate the combined association of obesity, diabetes mellitus (DM), and cardiovascular disease (CVD) with severe COVID-19 outcomes in adult and elderly inpatients.

Design: Cross-sectional study based on registry data from Brazil's influenza surveillance system.

Setting: Public and private hospitals across Brazil.

Participants: Eligible population included 21,942 inpatients aged ≥20 years with positive RT-PCR test for SARS-CoV-2 until Jun 9th, 2020.

Main outcome measures: Severe COVID-19 outcomes were non-invasive and invasive mechanical ventilation use, ICU admission, and death. Multivariate analyses were conducted separately for adults (20-59 years) and elders (≥60 years) to test the combined association of obesity (without and with DM and/or CVD) and degrees of obesity with each outcome.

Results: A sample of 8,848 adults and 12,925 elders were included. Among adults, obesity with DM and/or CVD showed higher prevalence of invasive (PR 3.76, 95%CI 2.82-5.01) and non-invasive mechanical ventilation use (2.06, 1.58-2.69), ICU admission (1.60, 1.40-1.83), and death (1.79, 1.45-2.21) compared with the group without obesity, DM, and CVD. In elders, obesity alone (without DM and CVD) had the highest prevalence of ICU admission (1.40, 1.07-1.82) and death (1.67, 1.00-2.80). In both age groups, obesity alone and combined with DM and/or CVD showed higher prevalence in all outcomes than DM and/or CVD. A dose-response association was observed between obesity and death in adults: class I 1.32 (1.05-1.66), class II 1.41 (1.06-1.87), and class III 1.77 (1.35-2.33).

Conclusions: The combined association of obesity, diabetes, and/or CVD with severe COVID-19 outcomes may be stronger in adults than in elders. Obesity alone and combined with DM and/or CVD had more impact on the risk of COVID-19 severity than DM and/or CVD in both age groups. The study also supports an independent relationship of obesity with severe outcomes, including a dose-response association between degrees of obesity and death in adults.

For peer review only

Article summary

Strengths and limitations of this study:

- This is the first study that describes the independent and combined relationship of obesity with COVID-19 severity in Brazil, one of the biggest epicenters of the pandemic worldwide.
- The study was based on registry data of a large nationwide sample of patients admitted, due to severe SARS-CoV-2 infection, to public and private hospitals across the country.
- The large sample size and data availability allowed us to analyze the combined association of obesity, diabetes and cardiovascular disease with severe COVID-19 outcomes, separately by age groups and controlled by important confounding variables, e.g. underlying comorbidities.
- The cross-sectional study design does not allow causal inference, and generalization of results must be cautious since only hospitalized cases of severe COVID-19 were included.
- As the study used routinely collected data, which has not been designed primarily for research purposes, it may bring well-known limitations related to missing, underestimation, and potential misclassification.

For peer review only

Introduction

The coronavirus disease 2019 (COVID-19) pandemic, caused by the severe acute respiratory syndrome coronavirus 2 (SARS-CoV-2), as of 11 July 2021, has already reached more than 185 million infected people and more than 4 million deaths in all continents.[1] Individuals with advanced age and chronic diseases, including cardiometabolic diseases, are considered groups at major risk for complications and severe illness from COVID-19.[2,3] Obesity has been shown as an independent risk factor for COVID-19 disease.[4-6] High body mass index (BMI) has been mentioned as a significant risk factor for COVID-19, according to early clinical reports from China,[7] Italy,[8] France,[9] Mexico,[10] and United States of America.[11] Several studies have demonstrated that obesity is leading to considerably worse COVID-19 outcomes, especially greater risk of hospital and intensive care unit (ICU) admission, invasive mechanical ventilation, and death.[11-14]

The COVID-19 pandemic is rapidly spreading worldwide, especially in the Americas, where obesity is already a prevalent and important public health problem.[15-16] Brazil is currently one of the biggest epicenters of the COVID-19 pandemic worldwide, with more than 18.9 million cases and 528 thousand deaths until July 11, 2021.[1] In 2018, the prevalence of adult overweight and obesity in Brazil was estimated at 55.7% and 19.8%, respectively.[17] This obesogenic profile of the Brazilian population contributes, among other factors, to the high prevalence of obesity-related diseases, such as type 2 diabetes mellitus (DM) and cardiovascular diseases (CVD), in the country.[18] The fact that individuals with obesity also have more comorbidity diseases, which are either risk factors for COVID-19 severity and death, makes obesity particularly ominous in COVID-19 disease.[10-13]

Several characteristics that can influence the clinical evolution of individuals infected with COVID-19, such as obesity, have been independently documented.[5-6,19] However, evidence is yet unclear on the combined effect that obesity and obesity-related comorbidities play in COVID-19 severity, especially, in different age groups. We aimed in this study to investigate the combined association of obesity, diabetes, and cardiovascular disease with mechanical ventilation use, ICU admission, and

For peer review only

For peer review only - http://bmjopen.bmj.com/site/about/guidelines.xhtml

death in a large sample of adult and elderly patients hospitalized with COVID-19 in Brazil. We also explored the independent association between degrees of obesity and the mentioned outcomes.

Methods

Study Design and Population

This is a cross-sectional study based on registry data from SIVEP-Gripe (Sistema de Informação de Vigilância Epidemiológica da Gripe), an influenza surveillance system of Brazil's Ministry of Health. The study used the publicly available dataset of SIVEP-Gripe, which includes de-identified data on cases of severe acute respiratory syndrome across public and private hospitals in Brazil.[20] These data were obtained through the Rede CoVida's integrated data platform that has been built with official, open, and authorized data for the production of knowledge about the COVID-19 pandemic.

Our study population was composed of patients aged 20 years or older, hospitalized for severe acute respiratory syndrome, with positive RT-PCR test for SARS-CoV-2, and final diagnosis for COVID-19 until Jun 9th, 2020. Only cases with complete data on demographic characteristics and comorbidities and plausible BMI values were included in the study.

Exposure Variable

Obesity was defined as BMI equal to or greater than 30 kg/m^2, according to the cutoff points proposed by the World Health Organization[21] and the Pan American Health Organization[22] for adults and elders, respectively. BMI was calculated by health professionals in the hospital from directly measured or patient self-reported height and weight. Guidelines for the collection and analysis of anthropometric data in health services have been previously standardized by the Ministry of Health.[23] BMI values <12 or >70 kg/m^2 were considered implausible and excluded.[24] Because BMI in the SIVEP-Gripe is a mandatory and complete information for patients diagnosed or self-reported with obesity only, the nutritional status of all patients were confirmed by a dichotomous variable (no/yes) on the existence of obesity available in the dataset. Likewise, information on the existence of diabetes

For peer review only

and any chronic cardiovascular disease was obtained from dichotomous questions (yes/no), which were answered based on patient or family's report or medical diagnosis.

We created a polytomous four-category variable to evaluate the separate and combined exposure of obesity, diabetes and cardiovascular disease: none/reference (no existence of obesity, diabetes and cardiovascular disease), OB (only existence of obesity), OB + DM and/or CVD (existence of obesity with diabetes and/or cardiovascular disease), and DM and/or CVD (existence of diabetes and/or cardiovascular disease). We also analyzed obesity in adults according to the following degrees of severity based on WHO reference[21]: no obesity (<30 kg/m^2), obesity class I (≥ 30-34.9 kg/m^2), obesity class II (≥ 35-39.9 kg/m^2), and obesity class III (≥ 40 kg/m^2). Due to the unavailability of BMI cutoff points to classify the degree of obesity in elders, this analysis was only performed for adults.

Outcome Variables

The severe COVID-19 outcomes were mechanical ventilation use, ICU admission, and death. Information on the use of mechanical ventilation by the patient was obtained and analyzed as a polytomous three-category variable (no use/ use of non-invasive ventilation/ use of invasive ventilation). ICU admission was obtained and analyzed as a dichotomous variable (no/ yes). Death was analyzed as a dichotomous variable based on the patient's endpoint outcome (cure/ death).

Covariates

Demographic and comorbidity information were selected as descriptive and confounding variables.[2] Age in years was calculated from birth and notification dates. Sex was obtained as a dichotomous variable (female/ male). The preexistence of each comorbidity was also obtained as a dichotomous variable (no/ yes): chronic pulmonary disease, asthma, chronic kidney disease, chronic hematologic disease, neurological disease, chronic liver disease, and immunodeficiency/ immunosuppression.

Statistical Analysis

All analyses were subdivided into adults (≥ 20 and < 60 years) and elders (≥ 60 years). For descriptive analyses, absolute and relative frequencies were calculated for the demographic and comorbidity

For peer review only

variables according to the main exposure variable. Multinomial logistic regression models were conducted to test the association of obesity (without and with diabetes and/or CVD) with non-invasive and invasive mechanical ventilation use. To test the association of this exposure variable with ICU admission and death, simple logistic regression models were performed. Same models were analyzed considering the degree of obesity as the main exposure variable for adults. Given the high prevalence of the analyzed outcome that could overestimate odds ratio,[25] crude and adjusted estimates were interpreted based on the prevalence ratio (PR) and 95% confidence intervals (95%CI). These estimates were obtained from logistic models using delta method, function 'prLogisticDelta', which is implemented in R and available in the package 'prLogistic'. Adjusted models included a set of confounding variables selected according to the current literature on obesity and severe COVID-9 risk factors:[26,27] sex, age (years), and the preexistence of chronic pulmonary disease, asthma, kidney disease, hematologic disease, neurological disease, liver disease, and immunodeficiency/ immunosuppression. The models that tested the degrees of obesity were also adjusted for DM and CVD. All analyses were performed using Stata version 15.1 (Stata Corporation, College Station, USA) and R version 3.6.1 (R Foundation for Statistical Computing, Austria).

Sensitivity analysis

Because detailed information on the comorbidities were not available (e.g., duration, severity), serious comorbidities such as chronic pulmonary diseases and immunosuppression were tested as exclusion criteria instead confounding variables in a sensitivity analysis. Same multivariate logistic models, using 'death' as outcome variable, were conducted separately for adults and elders, excluding the cases of pulmonary diseases and immunosuppression.

Patient and Public Involvement

As the study exclusively used publicly available de-identified data, it was not possible to involve patients or the public in the design, or conduct, or reporting, or dissemination plans of our research.

Results

For peer review only

During the study period, 21,942 individuals registered in the SIVEP-Gripe were ≥ 20 years old, hospitalized, tested positive for SARS-CoV-2, and had complete demographic and comorbidity information (**Figure 1**). Of these, 169 (0.8%) were excluded due to implausible values of BMI. Of the 21,773 individuals included in the study, 8,848 (40.3%) were adults aged between 20-59 years, and 12,925 (59.6%) were elders aged 60 years or older. Since some patients were still hospitalized on the study endpoint date, information for some outcomes were incomplete. The study samples included in the analysis of each outcome were 8,075 adults and 11,829 elders for mechanical ventilation, 8,414 adults and 12,222 for ICU admission, and 6,565 adults and 9,943 elders for death. Based on demographic and clinical characteristics, the analytical samples in each outcome were very similar to the overall study population and the excluded samples (**Supplementary Table 1**).

The prevalence of obesity was 9.7% in adults and 3.5% in elders. The frequency of obesity without and with DM and/or CVD was respectively 4.6% and 5.1% in adults and 0.7% and 2.8% in elders. Non-invasive and invasive mechanical ventilation were respectively required by 45.0% and 21.2% of adults and 47.0% and 30.0% of elders. ICU admission was needed by 35.4% of adults and 43.6% of elders. Death occurred in 31.1% of adult and 63.0% of elderly patients (**Table 1-2**).

In the adjusted analyses for adults, obesity alone (without DM and CVD) was associated with an increased prevalence of invasive (PR 2.69, 95%CI 1.98-3.65) and non-invasive mechanical ventilation need (PR 2.13, 95%CI 1.64-2.78), ICU admission (PR 1.31, 95%CI 1.13-1.53), and death (PR 1.33, 95%CI 1.05-1.69) when compared with the group without obesity, DM, and CVD. Obesity with DM and/or CVD was associated with an even higher prevalence of invasive mechanical ventilation (PR 3.76, 95%CI 2.82-5.01) and non-invasive ventilation use (PR 2.06, 95%CI 1.58-2.69), ICU admission (PR 1.60, 95%CI 1.40-1.83), and death in adults (PR 1.79, 95%CI 1.45-2.21). The subgroup of adults with DM and/or CVD showed in general the lowest prevalence ratios for all analyzed outcomes than the subgroups with the presence of obesity alone or combined (**Table 3**).

Among elders, obesity without DM and CVD was independently associated with a higher prevalence of ICU admission (PR 1.40, 95%CI 1.07-1.82) and death (PR 1.67, 95%CI 1.00-2.80). To a lesser

For peer review only

extent, obesity with DM and/or CVD was also associated with an increased prevalence of invasive mechanical ventilation need (PR 1.66, 95%CI 1.22-2.27), ICU admission (PR 1.37, 95%CI 1.19-1.59), and death (PR 1.39, 95%CI 1.07-1.80). Elders with DM and/or CVD had the lowest prevalence ratios for the analyzed outcomes than the group of elders with obesity alone or combined (**Table 3**).

In the analyses by the degree of obesity, we did not observe much difference in the prevalence of adverse outcomes, except for the prevalence of death that increased with the severity of obesity: Class I 1.32 (95%CI 1.05-1.66), Class II 1.41 (1.06-1.87), and Class III 1.77 (1.35-2.33) (**Table 4**).

The sensitivity analysis, excluding the cases of chronic pulmonary diseases and immunosuppression (**Supplementary Tables 2-3**), showed no difference in the results when compared with the estimates described above which were instead adjusted for these comorbidities. Only small differences in the magnitude of the associations, but not in the direction and significance, were observed.

Discussion

This is the first study that describe the relationship of obesity and COVID-19 in Brazil, based on a large nationwide sample of adults and elders tested positive for SARS-CoV-2 and admitted to public and private hospitals. Our results highlights that obesity with DM and/or CVD was associated with higher rates of invasive mechanical ventilation use, ICU admission, and death in adults, while obesity alone (without DM and CVD) was associated with higher rates of ICU admission and death among elders. In both age groups, obesity alone and obesity combined with DM and/or CVD had more impact on the risk of all severe COVID-19 outcomes than the subgroup with DM and/or CVD. The study also supports the independent association of obesity with the analyzed outcomes and a dose-response association between degrees of obesity and death in adults.

Some mechanisms related to the role of obesity and related diseases in worsening the clinical condition of patients affected by SARS-CoV-2 have been pointed out: i) greater body weight causes less elasticity of the chest wall and less total compliance of the respiratory system, leading to a restriction of the ventilation and the excursion of the diaphragm, making difficult the airway

For peer review only

management in patients with obesity;[28] ii) obesity is associated with sleep apnea syndrome and chronic obstructive pulmonary disease, which lead to surfactant dysfunction and impede the proper functioning of the airways;[29] iii) obesity is a metabolic and inflammatory disease, which is associated with the development or worsening of other chronic and endocrine comorbidities (e.g. type 2 diabetes, hypertension, dyslipidemia and CVD) that can modify innate and adaptive immune responses, making the immune system more vulnerable to infections and less responsive to antivirals and antimicrobial drugs;[16] iv) glycemic decompensation, common in patients with obesity, is associated with impaired ventilation function.[29]

It is important to note that the COVID-19 pandemic imposes a double burden of disease, especially among the elderly individuals, since the prevalence of diabetes, hypertension, cardiovascular diseases, and other comorbidities associated with COVID-19 severity increases with age.[3,30] However, our study suggests that obesity combined with diabetes and/or cardiovascular disease may offer higher risk of COVID-19 severity for adults although the overall prevalence of diseases and rates of ICU admission and mortality were higher in elders. Obesity alone seemed to provide higher risk of severe outcomes, especially death, in elders. As 1.00 remains a plausible value for the PR, according to its confidence intervals, we cannot conclude that the association of obesity alone with death in elders is clinically relevant. A larger sample would be needed to more fully address this.

Few studies to-date have explored the combined and additional effect of obesity on COVID-19 severity.[13,31] A study investigated the patterns of multimorbidity among fatal cases of COVID-19 in Colombia.[31] Similar to our study, the authors found that obesity alone or with other diseases was associated with a higher risk of COVID-19 fatality among young people. Furthermore, a population-based study in Mexico observed that the addition of obesity to any number of comorbidities significantly increased the risk of COVID-19 lethality.[13] Using a causally ordered mediation analysis, this study also found that 49.5% of the effect of diabetes on COVID-19 lethality was mediated by obesity, particularly in early-onset cases < 40 years of age.

For peer review only

Other studies also suggest that obesity is independently associated with severe outcomes of COVID-19, regardless of age and other associated comorbidities.[11-14] A large study in Mexico[13] showed that patients with obesity had higher rates of ICU admission and were more likely to be intubated in relation to patients without obesity. This study also found a five-fold increased risk of mortality due to COVID-19 in patients with obesity.[13] In a hospital-based study in France, it was observed that BMI > 35 kg/m^2 was associated with the need for invasive mechanical ventilation.[14]

Few studies to-date have similarly found a dose-response association between degrees of obesity and COVID-19 death.[32] Based on care records of 17,278,392 UK adults, the study showed that the risk of COVID-19 death increases independently with the degree of obesity: 30-34.9 kg/m^2 (HR 1.05), 35-39.9 kg/m^2 (1.40), and ≥40 kg/m^2 (2.66).[32] Other studies have evidenced the association of obesity with COVID-19 complications and death among adults.[12,33] A hospital-based study in New York City showed that morbid obesity (BMI ≥ 40 kg/m^2) is strongly and independently associated with death in hospitalized patients younger than 50 years.[33] Another study in New York City found a similar dose-response relationship between degrees of obesity and acute and critical care.[12] Patients less than 60 years old with BMI between 30 and 34.9 kg/m^2 (obesity class I) were 2.0 and 1.8 times more likely to be respectively admitted for acute care (general hospital admission) and critical care (ICU admission or invasive ventilator) compared to individuals with BMI < 30 kg/m^2. Patients of the same age group with BMI ≥ 35 kg/m^2 (obesity class II and III) showed 2.2 and 3.6 more chances of being hospitalized for acute and critical care, respectively.[12]

Strengths and limitations

One of the greatest strengths of the study was the use of SIVEP-Gripe dataset. Because severe acute respiratory syndrome is a condition of compulsory notification in both public and private hospitals,[34] we have a nationwide representative sample of patients hospitalized for severe COVID-19 in Brazil. In addition, the large sample sizes allowed us to analyze adults and elders separately, as well as the degrees of obesity which dose-response association with death was evidenced. The availability of important confounding variables (sex, age, and preexisting comorbidities) to control the estimated

For peer review only

associations, as well as hospital outcomes and mortality of COVID-19, was another differential of the study. Only patients with positive RT-PCR test for SARS-CoV-2 and final diagnosis for COVID-19 were included which gives greater precision on the studied population. The availability and use of data from health surveillance systems may be a lesson from Brazil that other countries can learn for obtaining routine and timely data to guide health systems and research in preparing and responding to pandemics before and during their course.

The study also has some limitations that must be considered. Because this is a cross-sectional study, a causal association cannot be inferred. As we used routinely collected data, which has not been designed primarily for research purposes, it may bring well-known limitations related to missing, underestimation, and potential misclassification. Obesity prevalence may have been underestimated due to the completeness of obesity and BMI data. Previous studies using SIVEP-Gripe data have also found a low prevalence of obesity in this population.[35,36] Better routine collection of height and weight data is still needed in clinical practice. Also, we believe that health professionals have adopted more the one method to collect weight and height information for BMI calculation, such as the patient's self-report and direct measure. Therefore, in addition to BMI which implausible values were checked and excluded, the classification of obesity was also confirmed from a dichotomous variable on the presence of obesity (no/yes). Although it is known that BMI does not distinguish between fat and lean body mass, and thus may lead to misclassification bias, BMI has been shown as a strong predictor of excess body fat and has been widely used in epidemiological studies.[15] Information for some outcomes were incomplete because some patients were still hospitalized on the study endpoint date. However, that did not represent a potential selection bias to our study. The analytical samples in each outcome had similar demographic and clinical characteristics than the overall study population and the excluded samples (Supplementary Table 1). As information on smoking was not available and ethnicity/race was very incomplete in the SIVEP-Gripe dataset, they were not included in the analysis. Additional studies are needed to further explore the relationship between obesity and severe COVID-

For peer review only

19, considering risk health behaviors and socioeconomic characteristics. Finally, the generalization of results must be cautious since the study included only hospitalized cases of COVID-19.

Conclusions

The combined association of obesity, diabetes, and/or cardiovascular disease with severe COVID-19 outcomes, especially ICU admission and death, may be stronger in adult than in elderly inpatients. In both age groups, obesity alone and obesity combined with DM and/or CVD had more impact on the risk of all severe COVID-19 outcomes than the subgroup with DM and/or CVD. The study also supports an independent relationship of obesity with the severe outcomes, including a dose-response association between degrees of obesity and death in adults. These findings suggest important implications for the clinical care of patients with obesity and severe COVID-19, such as the increased need of critical care and higher risk of death among these patients. Our study also supports the inclusion of people with obesity, independently of other preexisting comorbidities and age, in the high-risk and vaccine priority groups for protection from SARS-CoV-2 infection.

Acknowledgments

The authors thank the members of Rede CoVida's Epidemiology & Information Group for the work of identifying and collecting data related to COVID-19.

Author Contributions

NJS, RCRS, and RLF designed the study and analysis strategy. NJS, CASTS, and MYTI obtained, documented, and described the data. AJFF, CSST, ASR, FJOA, and IRF carried out the literature search. NJS and EJP performed the data analysis. NJS, RCRS, AJFF, CSST, ASR, FJOA, IRF, ESP and MLB contributed to data interpretation. NJS, AJFF, CSST, ASR, FJOA, and IRF drafted the manuscript. RCRS, ESP, MYTI, and MLB critically revised the manuscript. All authors read and approved the final manuscript.

Funding

For peer review only - http://bmjopen.bmj.com/site/about/guidelines.xhtml

All authors are affiliated to the Centre for Data and Knowledge Integration for Health (CIDACS) that is funded and supported by MCTI/ CNPq/ MS/ SCTIE/ Decit/ Bill & Melinda Gates Foundation's GCE Brazil (OPP1142172), Wellcome Trust (202912/Z/16/Z), the Brazilian Health Surveillance Secretariat, Ministry of Health, Bahia State, Research Support Foundation of the State of Bahia (FAPESB), the Research and Project Funding Agency (FINEP), and the Secretariat of Science and Technology of the State of Bahia (SECTI). Dr Paixão is a fellow supported by the Wellcome Trust (213589/Z/18/Z).

Competing Interests

None declared.

Patient Consent for Publication

Not required.

Data Availability Statement

Data is freely available without restriction at https://opendatasus.saude.gov.br/dataset/bd-srag-2020.

Code book and analytic code will be made available upon request from the corresponding author.

Ethics Statement

The study was conducted according to the guidelines laid down in the Declaration of Helsinki. As the study exclusively used publicly available de-identified data, ethics approval by a research ethics committee and informed consent are waived per Resolution n. 466/2012 of the National Health Council of Brazil's Commission of Ethics in Research.

For peer review only

References

1. World Health Organization. WHO Coronavirus Disease (COVID-19) Dashboard. https://covid19.who.int/. Accessed Jul 11, 2021.
2. Centers for Disease Control and Prevention. Coronavirus Disease 2019 (COVID-19). People Who Are at Increased Risk for Severe Illness. https://www.cdc.gov/coronavirus/2019-ncov/need-extra-precautions/people-at-increased-risk.html. Accessed July 21, 2020.
3. Liu H, Chen S, Liu M, Nie H, Lu H. Comorbid Chronic Diseases are Strongly Correlated with Disease Severity among COVID-19 Patients: A Systematic Review and Meta-Analysis. *Aging Dis*. 2020;11(3):668-678. doi:10.14336/ad.2020.0502.
4. Alberca RW, Oliveira LM, Branco ACCC, Pereira NZ, Sato MN. Obesity as a risk factor for COVID-19: an overview [published online June 15, 2020]. *Crit Rev Food Sci Nutr*. doi:10.1080/10408398.2020.1775546.
5. Sattar N, McInnes IB, McMurray JJV. Obesity Is a Risk Factor for Severe COVID-19 Infection: Multiple Potential Mechanisms. *Circulation*. 2020;142(1):4-6. doi:10.1161/circulationaha.120.047659.
6. Caussy C, Wallet F, Laville M, Disse E. Obesity is Associated with Severe Forms of COVID-19. *Obesity (Silver Spring)*. 2020;28(7):1175. doi:10.1002/oby.22842.
7. Li Q, Guan X, Wu P, et al. Early Transmission Dynamics in Wuhan, China, of Novel Coronavirus-Infected Pneumonia. *N Engl J Med*. 2020;382(13):1199-1207. doi:10.1056/nejmoa2001316.
8. Grasselli G, Zangrillo A, Zanella A, et al. Baseline Characteristics and Outcomes of 1591 Patients Infected with SARS-CoV-2 Admitted to ICUs of the Lombardy Region, Italy. *JAMA*. 2020;323(16):1574-1581. doi:10.1001/jama.2020.5394.
9. Caussy C, Pattou F, Wallet F, et al. Prevalence of obesity among adult inpatients with COVID-19 in France. *Lancet Diabetes Endocrinol*. 2020;8(7):562-564. doi:10.1016/s2213-8587(20)30160-1.
10. Hernández-Garduño E. Obesity is the comorbidity more strongly associated for Covid-19 in Mexico. A case-control study. *Obes Res Clin Pract*. 2020;14(4):375-379. doi:10.1016/j.orcp.2020.06.001.
11. Hajifathalian K, Kumar S, Newberry C, et al. Obesity is associated with worse outcomes in COVID-19: Analysis of Early Data From New York City. *Obesity (Silver Spring)*. 2020;28(9):1606-1612. doi:10.1002/oby.22923.
12. Lighter J, Phillips M, Hochman S, et al. Obesity in patients younger than 60 years is a risk factor for Covid-19 hospital admission. *Clin Infect Dis*. 2020;71(15):896-897. doi:10.1093/cid/ciaa415.
13. Bello-Chavolla OY, Bahena-López JP, Antonio-Villa NE, et al. Predicting Mortality Due to SARS-CoV-2: A Mechanistic Score Relating Obesity and Diabetes to COVID-19 Outcomes in Mexico. *J Clin Endocrinol Metab*. 2020;105(8):dgaa346. doi:10.1210/clinem/dgaa346.
14. Simonnet A, Chetboun M, Poissy J, et al. High Prevalence of Obesity in Severe Acute Respiratory Syndrome Coronavirus-2 (SARS-CoV-2) Requiring Invasive Mechanical Ventilation. *Obesity (Silver Spring)*. 2020;28(7):1195-1199. doi:10.1002/oby.22831.
15. NCD Risk Factor Collaboration (NCD-RisC). Worldwide trends in body-mass index, underweight, overweight, and obesity from 1975 to 2016: a pooled analysis of 2416 population-based measurement studies in 128.9 million children, adolescents, and adults. *Lancet*. 2017;390(10113):2627-2642. doi:10.1016/s0140-6736(17)32129-3.
16. GBD 2015 Obesity Collaborators. Health Effects of Overweight and Obesity in 195 Countries over 25 Years. *N Engl J Med*. 2017;377(1):13-27. doi:10.1056/nejmoa1614362.
17. Brasil. Ministério da Saúde. Vigitel Brasil 2018: vigilância de fatores de risco e proteção para doenças crônicas por inquérito telefônico. Brasília, DF: Ministério da Saúde; 2019. https://portalarquivos2.saude.gov.br/images/pdf/2019/julho/25/vigitel-brasil-2018.pdf. Accessed July 10, 2020.

18. GBD 2016 Brazil Collaborators. Burden of disease in Brazil, 1990-2016: a systematic subnational analysis for the Global Burden of Disease Study 2016. *Lancet*. 2018;392(10149):760-775. doi:10.1016/s0140-6736(18)31221-2.
19. Flint SW, Tahrani AA. COVID-19 and obesity-lack of clarity, guidance, and implications for care. *Lancet Diabetes Endocrinol*. 2020;8(6):474-475. doi:10.1016/s2213-8587(20)30156-x.
20. Brasil. Ministério da Saúde. Severe Acute Respiratory Syndrome dataset - including COVID-19 data. 2020. https://opendatasus.saude.gov.br/dataset/bd-srag-2020. Accessed July 11, 2020.
21. World Health Organization. Physical status: the use and interpretation of anthropometry. Geneva: WHO; 1995. https://apps.who.int/iris/handle/10665/37003. Accessed July 11, 2020.
22. Organización Panamericana de la Salud. División de Promoción y Protección de la Salud (HPP). Encuesta sobre Salud, Bienestar y Envejecimiento (SABE) en América Latina el Caribe: Informe Preliminar (Internet). In: XXXVI Reunión del Comité asesor de investigaciones em Salud; 9-11 jun 2001; Kingston: OPAS, 2002. http://envejecimiento.csic.es/documentos/documentos/paho-salud-01.pdf. Accessed July 11, 2020.
23. Brasil. Ministério da Saúde. Guidelines for the Collection and Analysis of Anthropometric Data in Health Services. Technical Standard for the Food and Nutrition Surveillance System. Brasília, DF: Ministério da Saúde; 2011.
24. Cheng FW, Gao X, Mitchell DC, et al. Body mass index and all-cause mortality among older adults. *Obesity (Silver Spring)*. 2016;24(10):2232-39. doi:10.1002/oby.21612.
25. Bruce N, Pope D, Stanistreet D. Quantitative methods for health research: a practical interactive guide to epidemiology and statistics (Second ed.). Hoboken, NJ. p. 16. ISBN 978-1-118-66526-8. OCLC 992438133.
26. VanderWeele TJ. Principles of confounder selection. Eur J Epidemiol. 2019;34(3):211-219. doi: 10.1007/s10654-019-00494-6.
27. Popkin BM, Du S, Green WD, et al. Individuals with obesity and COVID-19: A global perspective on the epidemiology and biological relationships. Obes Rev. 2020;21(11):e13128. doi: 10.1111/obr.13128.
28. Honce R, Schultz-Cherry S. Impact of Obesity on Influenza A Virus Pathogenesis, Immune Response, and Evolution. *Front Immunol*. 2019;10:1071. doi:10.3389/fimmu.2019.01071.
29. Puig-Domingo M, Marazuela M, Giustina A. COVID-19 and endocrine diseases. A statement from the European Society of Endocrinology. *Endocrine*. 2020;68(1):2-5. doi:10.1007/s12020-020-02294-5.
30. Docherty AB, Harrison EM, Green CA, et al. Features of 20 133 UK patients in hospital with covid-19 using the ISARIC WHO Clinical Characterisation Protocol: prospective observational cohort study. *BMJ*. 2020;369:m1985. doi:10.1136/bmj.m1985.
31. Fernández-Niño JA, Guerra-Gómez JA, Idrovo AJ. Multimorbidity patterns among COVID-19 deaths: proposal for the construction of etiological models. *Rev Panam Salud Publica*. 2020;44:e166. doi:10.26633/RPSP.2020.166.
32. Williamson EJ, Walker AJ, Bhaskaran K, et al. Factors associated with COVID-19-related death using OpenSAFELY. *Nature*. 2020;584(7821):430-436. doi:10.1038/s41586-020-2521-4.
33. Klang E, Kassim G, Soffer S, et al. Morbid Obesity as an Independent Risk Factor for COVID-19 Mortality in Hospitalized Patients Younger than 50. *Obesity (Silver Spring)*. 2020;28(9):1595-1599. doi:10.1002/oby.22913.
34. Cantarino L, Merchan-Hamann E. Influenza in Brazil: surveillance pathways. *J Infect Dev Ctries*. 2016;10(1):13-23. doi:10.3855/jidc.7135.
35. de Souza WM, Buss LF, Candido DDS, et al. Epidemiological and clinical characteristics of the COVID-19 epidemic in Brazil. *Nat Hum Behav*. 2020;4(8):856-865. doi:10.1038/s41562-020-0928-4.
36. Baqui P, Bica I, Marra V, Ercole A, van der Schaar M. Ethnic and regional variations in hospital mortality from COVID-19 in Brazil: a cross-sectional observational study. *Lancet Glob Health*. 2020;8(8):e1018-e1026. doi: 10.1016/S2214-109X(20)30285-0.

Figure legends

Figure 1. Selection of the study population from SIVEP-Gripe.

SIVEP-Gripe: Influenza Epidemiological Surveillance Information System.

For peer review only

Table 1. Demographic characteristics, comorbidities, hospitalization outcomes and death according to the combined exposure of obesity, diabetes, and/or cardiovascular diseases in adults with severe COVID-19.

	Total		None		OB		OB + DM and/or CVD		DM and/or CVD	
	n	%	n	%	n	%	n	%	n	%
Overall	8848	100.0	3161	35.7	409	4.6	452	5.1	4826	54.6
**Sex**										
Female	3774	42.7	1511	40.0	165	4.4	199	5.3	1899	50.3
Male	5074	57.4	1650	32.5	244	4.8	253	5.0	2927	57.7
**Age**										
< 40 years	1976	22.3	1064	53.9	188	9.5	102	5.2	622	31.5
≥ 40 years	6872	77.7	2097	30.5	221	3.2	350	5.1	4204	61.2
**Chronic pulmonary disease**										
No	8502	96.1	2969	34.9	388	4.6	435	5.1	4710	55.4
Yes	346	3.9	192	55.5	21	6.1	17	4.9	116	33.5
**Asthma**										
No	8184	92.5	2728	33.3	383	4.7	414	5.1	4659	56.9
Yes	664	7.5	433	65.2	26	3.9	38	5.7	167	25.2
**Chronic kidney disease**										
No	8297	93.8	2958	35.7	399	4.8	434	5.2	4506	54.3
Yes	551	6.2	203	36.8	10	1.8	18	3.3	320	58.1
**Chronic hematologic disease**										
No	8710	98.4	3081	35.4	406	4.7	445	5.1	4778	54.9
Yes	138	1.6	80	58.0	3	2.2	7	5.1	48	34.8
**Chronic neurological disease**										
No	8588	97.1	3014	35.1	406	4.7	442	5.2	4726	55.0
Yes	260	2.9	147	56.5	3	1.2	10	3.9	100	38.5
**Chronic liver disease**										
No	8684	98.2	3083	35.5	406	4.7	443	5.1	4752	54.7
Yes	164	1.9	78	47.6	3	1.8	9	5.5	74	45.1
**Immunosuppression**										
No	8276	93.5	2777	33.6	393	4.8	440	5.3	4666	56.4
Yes	572	6.5	384	67.1	16	2.8	12	2.1	160	28.0
**Mechanical ventilation***										
No	2727	33.8	1144	42.0	93	3.4	88	3.2	1402	51.4
Non-invasive	3634	45.0	1178	32.4	192	5.3	190	5.2	2074	57.1
Invasive	1714	21.2	529	30.9	101	5.9	150	8.8	934	54.5
**ICU admission***										
No	5438	64.6	2025	37.2	235	4.3	222	4.1	2956	54.4
Yes	2976	35.4	1007	33.8	163	5.5	216	7.3	1590	53.4
**Death***										
No	4525	68.9	1699	37.6	211	4.7	200	4.4	2415	53.4
Yes	2040	31.1	640	31.4	92	4.5	140	6.9	1168	57.3

OB: obesity (BMI≥30 kg/m^2), DM: diabetes mellitus, CVD: cardiovascular disease, ICU: intensive care unit.
* Mechanical ventilation (n=8075), ICU admission (n=8414), and death (n=6565).

For peer review only

Table 2. Demographic characteristics, comorbidities, hospitalization outcomes and death according to the combined exposure of obesity, diabetes, and/or cardiovascular diseases in elders with severe COVID-19.

	**Total**		**None**		**OB**		**OB + DM and/or CVD**		**DM and/or CVD**	
	**n**	**%**	**n**	**%**	**n**	**%**	**n**	**%**	**n**	**%**
Overall	12925	100.0	2837	21.9	91	0.7	358	2.8	9639	74.6
**Sex**										
Female	5968	46.2	1232	20.6	52	0.9	209	3.5	4475	75.0
Male	6957	53.8	1605	23.1	39	0.6	149	2.1	5164	74.2
**Age**										
< 80 years	9355	72.4	2011	21.5	77	0.8	309	3.3	6958	74.4
≥ 80 years	3570	27.6	826	23.1	14	0.4	49	1.4	2681	75.1
**Chronic pulmonary disease**										
No	11885	92.0	2494	21.0	85	0.7	325	2.7	8981	75.6
Yes	1040	8.1	343	33.0	6	0.6	33	3.2	658	63.3
**Asthma**										
No	12474	96.5	2687	21.5	90	0.7	336	2.7	9361	75.0
Yes	451	3.5	150	33.3	1	0.2	22	4.9	278	61.6
**Chronic kidney disease**										
No	11882	91.9	2608	22.0	85	0.7	311	2.6	8878	74.7
Yes	1043	8.1	229	22.0	6	0.6	47	4.5	761	73.0
**Chronic hematologic disease**										
No	12728	98.5	2751	21.6	91	0.7	354	2.8	9532	74.9
Yes	197	1.5	86	43.7	0	0.0	4	2.0	107	54.3
**Chronic neurological disease**										
No	11871	91.9	2511	21.2	89	0.8	338	2.9	8933	75.3
Yes	1054	8.2	326	30.9	2	0.2	20	1.9	706	67.0
**Chronic liver disease**										
No	12734	98.5	2777	21.8	87	0.7	353	2.8	9517	74.7
Yes	191	1.5	60	31.4	4	2.1	5	2.6	122	63.9
**Immunosuppression**										
No	12303	95.2	2558	20.8	87	0.7	342	2.8	9316	75.7
Yes	622	4.8	279	44.9	4	0.6	16	2.6	323	51.9
**Mechanical ventilation***										
No	2725	23.0	626	23.0	18	0.7	70	2.6	2011	73.8
Non-invasive	5557	47.0	1164	21.0	38	0.7	141	2.5	4214	75.8
Invasive	3547	30.0	767	21.6	29	0.8	133	3.8	2618	73.8
**ICU admission***										
No	6898	56.4	1578	22.9	41	0.6	168	2.4	5111	74.1
Yes	5324	43.6	1107	20.8	44	0.8	181	3.4	3992	75.0
**Death***										
No	3684	37.1	823	22.3	21	0.6	95	2.6	2745	74.5
Yes	6259	63.0	1407	22.5	43	0.7	173	2.8	4636	74.1

OB: obesity (BMI≥30 kg/m^2), DM: diabetes mellitus, CVD: cardiovascular disease, ICU: intensive care unit.
* Mechanical ventilation (n=11829), ICU admission (n=12222), and death (n=9943).

For peer review only

Table 3. Combined association of obesity, diabetes, and/or cardiovascular disease with non-invasive and invasive mechanical ventilation use, intensive care unit admission, and death in adult and elderly patients hospitalized with severe COVID-19.

	Main exposure variable	Non-invasive mechanical ventilation*				Invasive mechanical ventilation*			
		Crude model		Adjusted model #		Crude model		Adjusted model #	
		PR	95%CI	PR	95%CI	PR	95%CI	PR	95%CI
	None	1.00		1.00		1.00		1.00	
**Adults**	OB	2.00	1.54-2.60	2.13	1.64-2.78	2.35	1.74-3.17	2.69	1.98-3.65
20-59 years	OB + DM and/or CVD	2.10	1.61-2.73	2.06	1.58-2.69	3.69	2.78-4.89	3.76	2.82-5.01
	DM and/or CVD	1.44	1.29-1.60	1.35	1.20-1.51	1.44	1.26-1.64	1.32	1.14-1.52
	None	1.00		1.00		1.00		1.00	
**Elders**	OB	1.14	0.64-2.01	1.22	0.69-2.16	1.31	0.72-2.39	1.43	0.78-2.61
≥ 60 years	OB + DM and/or CVD	1.08	0.80-1.47	1.15	0.84-1.55	1.55	1.14-2.11	1.66	1.22-2.27
	DM and/or CVD	1.13	1.01-1.26	1.14	1.01-1.27	1.06	0.94-1.20	1.10	0.97-1.24
		**ICU admission****				**Death*****			
		Crude model		Adjusted model #		Crude model		Adjusted model #	
		PR	95%CI	PR	95%CI	PR	95%CI	PR	95%CI
	None	1.00		1.00		1.00		1.00	
**Adults**	OB	1.23	1.08-1.40	1.31	1.13-1.53	1.11	0.92-1.33	1.33	1.05-1.69
20-59 years	OB + DM and/or CVD	1.48	1.33-1.65	1.60	1.40-1.83	1.50	1.30-1.74	1.79	1.45-2.21
	DM and/or CVD	1.05	0.99-1.12	1.03	0.95-1.12	1.19	1.10-1.29	1.16	1.03-1.30
	None	1.00		1.00		1.00		1.00	
**Elders**	OB	1.26	1.02-1.55	1.40	1.07-1.82	1.06	0.89-1.27	1.67	1.00-2.80
≥ 60 years	OB + DM and/or CVD	1.26	1.13-1.41	1.37	1.19-1.59	1.02	0.93-1.12	1.39	1.07-1.80
	DM and/or CVD	1.06	1.01-1.12	1.11	1.04-1.18	1.00	0.96-1.03	1.05	0.95-1.16

OB: obesity (BMI≥30 kg/m^2), DM: diabetes mellitus, CVD: cardiovascular disease, ICU: intensive care unit, PR: prevalence ratio, 95%CI: 95% confidence interval.
* Crude and adjusted multinomial logistic regression models for mechanical ventilation use in adults (n=8075) and elders (n=11829).
** Crude and adjusted logistic regression models for ICU admission in adults (n= 8414) and elders (n=12222).
*** Crude and adjusted logistic regression models for death in adults (n=6565) and elders (n=9943).
# Adjusted for sex, age in years, pulmonary disease, asthma, kidney disease, hematologic disease, neurological disease, liver disease, and immunosuppression.

For peer review only

Table 4. Independent association of degrees of obesity with non-invasive and invasive mechanical ventilation, intensive care unit admission, and death in hospitalized adults with severe COVID-19.

Main exposure variable	Non-invasive mechanical ventilation*				Invasive mechanical ventilation*			
	Crude model		Adjusted model #		Crude model		Adjusted model #	
	PR	95%CI	PR	95%CI	PR	95%CI	PR	95%CI
No obesity (< 30 kg/m^2)	1.00		1.00		1.00		1.00	
Obesity class I (≥ 30-34.9 kg/m^2)	1.78	1.35-2.33	1.91	1.45-2.51	2.59	1.93-3.47	3.00	2.22-4.05
Obesity class II (≥ 35-39.9 kg/m^2)	1.44	1.04-2.00	1.58	1.14-2.19	2.10	1.47-2.99	2.47	1.72-3.54
Obesity class III (≥ 40 kg/m^2)	1.70	1.19-2.44	1.88	1.31-2.69	2.51	1.71-3.70	3.00	2.03-4.45
	ICU admission**				Death**			
	Crude model		Adjusted model #		Crude model		Adjusted model #	
	PR	95%CI	PR	95%CI	PR	95%CI	PR	95%CI
No obesity (< 30 kg/m^2)	1.00		1.00		1.00		1.00	
Obesity class I (≥ 30-34.9 kg/m^2)	1.31	1.17-1.47	1.42	1.23-1.64	1.11	0.94-1.31	1.32	1.05-1.66
Obesity class II (≥ 35-39.9 kg/m^2)	1.34	1.16-1.54	1.46	1.23-1.74	1.16	0.95-1.42	1.41	1.06-1.87
Obesity class III (≥ 40 kg/m^2)	1.32	1.14-1.54	1.45	1.20-1.74	1.33	1.10-1.59	1.77	1.35-2.33

Degrees of obesity defined by the WHO cutoff points.
PR: prevalence ratio, 95%CI: 95% confidence interval.
* Crude and adjusted multinomial logistic regression models for mechanical ventilation use (n=8075).
** Crude and adjusted logistic regression models for ICU admission (n=8414) and mortality (n=6565).
# Adjusted for sex, age in years, diabetes mellitus, cardiovascular disease, pulmonary disease, asthma, kidney disease, hematologic disease, neurological disease, liver disease, and immunosuppression.

For peer review only

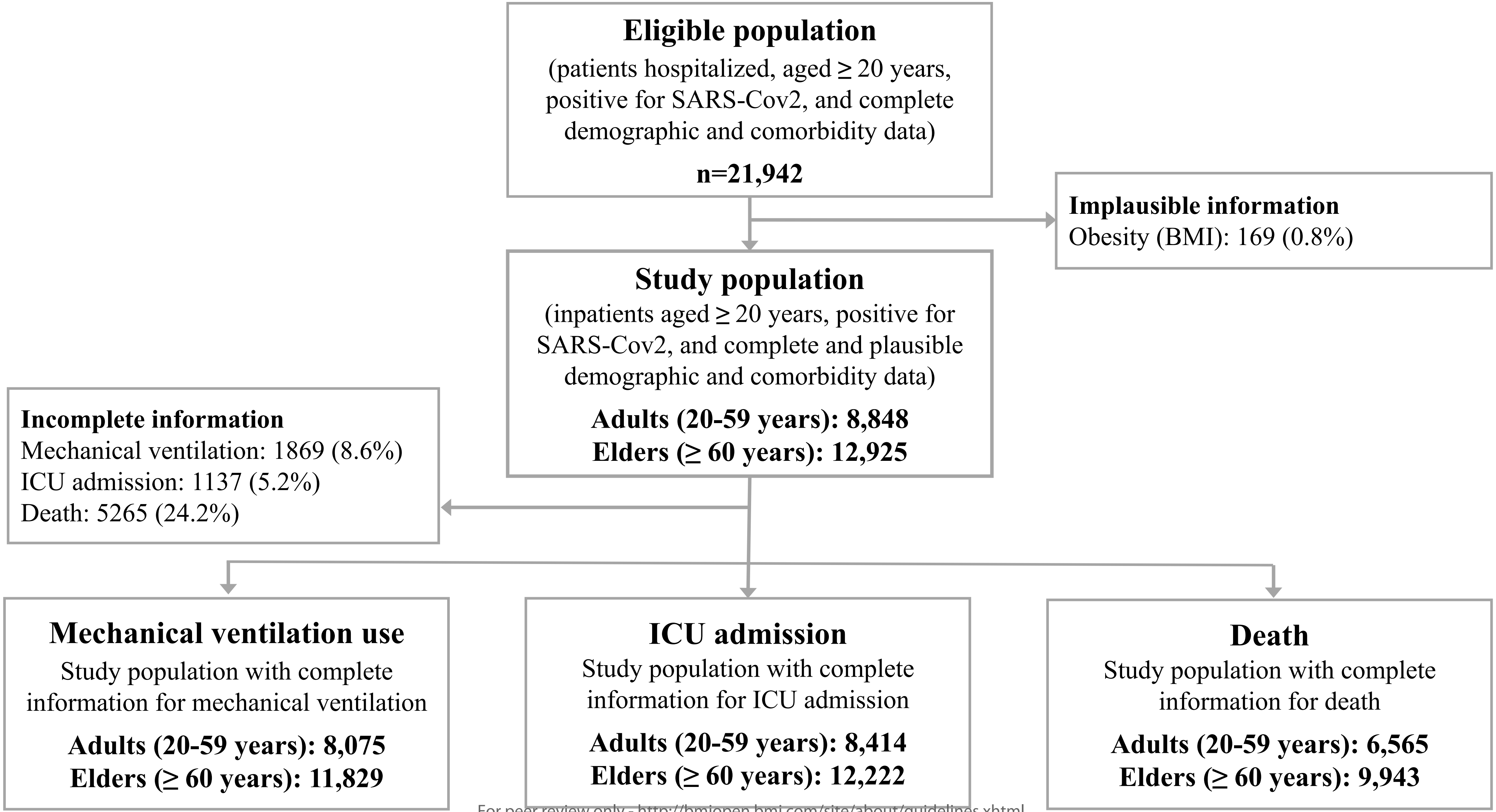
Eligible population
(patients hospitalized, aged ≥ 20 years, positive for SARS-Cov2, and complete demographic and comorbidity data)
n=21,942
Implausible information
Obesity (BMI): 169 (0.8%)
Study population
(inpatients aged ≥ 20 years, positive for SARS-Cov2, and complete and plausible demographic and comorbidity data)
Adults (20-59 years): 8,848
Elders (≥ 60 years): 12,925
Incomplete information
Mechanical ventilation: 1869 (8.6%)
ICU admission: 1137 (5.2%)
Death: 5265 (24.2%)
Mechanical ventilation use
Study population with complete information for mechanical ventilation
Adults (20-59 years): 8,075
Elders (≥ 60 years): 11,829
ICU admission
Study population with complete information for ICU admission
Adults (20-59 years): 8,414
Elders (≥ 60 years): 12,222
Death
Study population with complete information for death
Adults (20-59 years): 6,565
Elders (≥ 60 years): 9,943

Suppl. Table 1. Characteristics of the study population and study samples included and excluded of the analysis for each severe COVID-19 outcome.

	Study population*		Mechanical ventilation				ICU admission				Death			
			Included		Excluded		Included		Excluded		Included		Excluded	
	n	%	n	%	n	%	n	%	n	%	n	%	n	%
Overall	21,773	100.0	19,904	91.4	1,869	8.6	20,636	94.8	1,137	5.2	16,508	75.8	5,265	24.2
**Sex**														
Female	9,742	44.7	8,905	44.7	837	44.8	9,227	44.7	515	45.3	7,379	44.7	2,363	44.9
Male	12,031	55.3	10,999	55.3	1,032	55.2	11,409	55.3	622	54.7	9,129	55.3	2,902	55.1
**Age (years)**														
20-39	1,976	9.1	1,808	9.1	168	9.0	1,890	9.2	86	7.6	1,445	8.8	531	10.1
40-59	6,872	31.6	6,267	31.5	605	32.4	6,524	31.6	348	30.6	5,120	31.0	1,752	33.3
60-79	9,355	43.0	8,546	42.9	809	43.3	8,827	42.8	528	46.4	7,062	42.8	2,293	43.6
>= 80	3,570	16.4	3,283	16.5	287	15.4	3,395	16.5	175	15.4	2,881	17.5	689	13.1
**Obesity**														
No	20,463	94.0	18,661	93.8	1,802	96.4	19,366	93.9	1,097	96.5	15,533	94.1	4,930	93.6
Yes	1,310	6.0	1,243	6.2	67	3.6	1,270	6.2	40	3.5	975	5.9	335	6.4
**Diabetes**														
No	13,058	60.0	11,925	59.9	1,133	60.6	12,401	60.1	657	57.8	9,925	60.1	3,133	59.5
Yes	8,715	40.0	7,979	40.1	736	39.4	8,235	39.9	480	42.2	6,583	39.9	2,132	40.5
**Cardiovascular disease**														
No	10,391	47.7	9,400	47.2	991	53.0	9,858	47.8	533	46.9	7,866	47.7	2,525	48.0
Yes	11,382	52.3	10,504	52.8	878	47.0	10,778	52.2	604	53.1	8,642	52.4	2,740	52.0
**Chronic pulmonary disease**														
No	20,387	93.6	18,630	93.6	1,757	94.0	19,306	93.6	1,081	95.1	15,422	93.4	4,965	94.3
Yes	1,386	6.4	1,274	6.4	112	6.0	1,330	6.5	56	4.9	1,086	6.6	300	5.7
**Asthma**														
No	20,658	94.9	18,897	94.9	1,761	94.2	19,571	94.8	1,087	95.6	15,666	94.9	4,992	94.8
Yes	1,115	5.1	1,007	5.1	108	5.8	1,065	5.2	50	4.4	842	5.1	273	5.2
**Chronic kidney disease**														
No	20,179	92.7	18,420	92.5	1,759	94.1	19,106	92.6	1,073	94.4	15,255	92.4	4,924	93.5
Yes	1,594	7.3	1,484	7.5	110	5.9	1,530	7.4	64	5.6	1,253	7.6	341	6.5
**Chronic hematologic disease**														
No	21,438	98.5	19,597	98.5	1,841	98.5	20,313	98.4	1,125	98.9	16,245	98.4	5,193	98.6
Yes	335	1.5	307	1.5	28	1.5	323	1.6	12	1.1	263	1.6	72	1.4
**Chronic neurological disease**														
No	20,459	94.0	18,700	94.0	1,759	94.1	19,377	93.9	1,082	95.2	15,460	93.7	4,999	95.0
Yes	1,314	6.0	1,204	6.1	110	5.9	1,259	6.1	55	4.8	1,048	6.4	266	5.1

For peer review only

**Chronic liver disease**																
No	21,418	98.4	19,581	98.4	1,837	98.3	20,300	98.4	1,118	98.3	16,218	98.2	5,200	98.8
Yes	355	1.6	323	1.6	32	1.7	336	1.6	19	1.7	290	1.8	65	1.2
**Immunosuppression**														
No	20,579	94.5	18,792	94.4	1,787	95.6	19,485	94.4	1,094	96.2	15,571	94.3	5,008	95.1
Yes	1,194	5.5	1,112	5.6	82	4.4	1,151	5.6	43	3.8	937	5.7	257	4.9

For peer review only

Suppl. Table 2. Combined association of obesity, diabetes, and/or cardiovascular disease with death in adult and elderly patients hospitalized with severe COVID-19, excluding the cases of chronic pulmonary diseases and immunosuppression.

	**Main exposure variable**	**Death***	
		PR	95%CI
	None	1.00	
**Adults**	OB	1.38	1.07-1.79
20-59 years	OB + DM and/or CVD	1.86	1.49-2.32
	DM and/or CVD	1.16	1.03-1.32
	None	1.00	
**Elders**	OB	1.64	0.99-2.82
≥ 60 years	OB + DM and/or CVD	1.43	1.08-1.90
	DM and/or CVD	1.05	0.94-1.17

OB: obesity (BMI≥30 kg/m^2), DM: diabetes mellitus, CVD: cardiovascular disease, ICU: intensive care unit, PR: prevalence ratio, 95%CI: 95% confidence interval.
[#] Adjusted for sex, age in years, asthma, kidney disease, hematologic disease, neurological disease, and liver disease.
* Adjusted logistic regression model for death in adults (n=5889) and elders (n=8676).

For peer review only

Suppl. Table 3. Independent association of degrees of obesity with death in hospitalized adults with severe COVID-19, excluding the cases of chronic pulmonary disease and immunosuppression.

**Main exposure variable**	Death*	
	PR	95%CI
No obesity (< 30 kg/m^2)	1.00	
Obesity class I (≥ 30-34.9 kg/m^2)	1.39	1.09-1.77
Obesity class II (≥ 35-39.9 kg/m^2)	1.48	1.10-1.99
Obesity class III (≥ 40 kg/m^2)	1.79	1.34-2.41

Degrees of obesity defined by the WHO cutoff points.
PR: prevalence ratio, 95%CI: 95% confidence interval.
* Adjusted logistic regression model for death (n=5889).
[#] Adjusted for sex, age in years, diabetes mellitus, cardiovascular disease, asthma, kidney disease, hematologic disease, neurological disease, and liver disease.

For peer review only

The RECORD statement – checklist of items, extended from the STROBE statement, that should be reported in observational studies using routinely collected health data.

	Item No.	STROBE items	Location in manuscript where items are reported	RECORD items	Location in manuscript where items are reported
**Title and abstract**					
	1	(a) Indicate the study's design with a commonly used term in the title or the abstract (b) Provide in the abstract an informative and balanced summary of what was done and what was found		RECORD 1.1: The type of data used should be specified in the title or abstract. When possible, the name of the databases used should be included.<br><br>RECORD 1.2: If applicable, the geographic region and timeframe within which the study took place should be reported in the title or abstract.<br><br>RECORD 1.3: If linkage between databases was conducted for the study, this should be clearly stated in the title or abstract.	Page 1-2<br><br>Page 1-2<br><br>N/A
**Introduction**					
Background rationale	2	Explain the scientific background and rationale for the investigation being reported			Pages 4-5
Objectives	3	State specific objectives, including any prespecified hypotheses			Pages 4-5
**Methods**					
Study Design	4	Present key elements of study design early in the paper			Page 5
Setting	5	Describe the setting, locations, and relevant dates, including periods of recruitment, exposure, follow-up, and data collection			Page 5

For peer review only

Participants	6	*(a) Cohort study* - Give the eligibility criteria, and the sources and methods of selection of participants. Describe methods of follow-up<br>*Case-control study* - Give the eligibility criteria, and the sources and methods of case ascertainment and control selection. Give the rationale for the choice of cases and controls<br>*Cross-sectional study* - Give the eligibility criteria, and the sources and methods of selection of participants<br><br>*(b) Cohort study* - For matched studies, give matching criteria and number of exposed and unexposed<br>*Case-control study* - For matched studies, give matching criteria and the number of controls per case		RECORD 6.1: The methods of study population selection (such as codes or algorithms used to identify subjects) should be listed in detail. If this is not possible, an explanation should be provided.<br><br>RECORD 6.2: Any validation studies of the codes or algorithms used to select the population should be referenced. If validation was conducted for this study and not published elsewhere, detailed methods and results should be provided.<br><br>RECORD 6.3: If the study involved linkage of databases, consider use of a flow diagram or other graphical display to demonstrate the data linkage process, including the number of individuals with linked data at each stage.	Page 5
Variables	7	Clearly define all outcomes, exposures, predictors, potential confounders, and effect modifiers. Give diagnostic criteria, if applicable.		RECORD 7.1: A complete list of codes and algorithms used to classify exposures, outcomes, confounders, and effect modifiers should be provided. If these cannot be reported, an explanation should be provided.	Pages 5-6
Data sources/ measurement	8	For each variable of interest, give sources of data and details of methods of assessment (measurement).<br>Describe comparability of assessment methods if there is more than one group			Pages 5-6

For peer review only

Bias	9	Describe any efforts to address potential sources of bias			Pages 11-12
Study size	10	Explain how the study size was arrived at			Page 5
Quantitative variables	11	Explain how quantitative variables were handled in the analyses. If applicable, describe which groupings were chosen, and why			Pages 5-6
Statistical methods	12	(a) Describe all statistical methods, including those used to control for confounding<br>(b) Describe any methods used to examine subgroups and interactions<br>(c) Explain how missing data were addressed<br>(d) *Cohort study* - If applicable, explain how loss to follow-up was addressed<br>*Case-control study* - If applicable, explain how matching of cases and controls was addressed<br>*Cross-sectional study* - If applicable, describe analytical methods taking account of sampling strategy<br>(e) Describe any sensitivity analyses			Pages 6-7
Data access and cleaning methods		..		RECORD 12.1: Authors should describe the extent to which the investigators had access to the database population used to create the study population.	Page 5

For peer review only

				RECORD 12.2: Authors should provide information on the data cleaning methods used in the study.	
Linkage		..		RECORD 12.3: State whether the study included person-level, institutional-level, or other data linkage across two or more databases. The methods of linkage and methods of linkage quality evaluation should be provided.	N/A
**Results**					
Participants	13	(a) Report the numbers of individuals at each stage of the study (*e.g.*, numbers potentially eligible, examined for eligibility, confirmed eligible, included in the study, completing follow-up, and analysed)<br>(b) Give reasons for non-participation at each stage.<br>(c) Consider use of a flow diagram		RECORD 13.1: Describe in detail the selection of the persons included in the study (*i.e.,* study population selection) including filtering based on data quality, data availability and linkage. The selection of included persons can be described in the text and/or by means of the study flow diagram.	Page 8
Descriptive data	14	(a) Give characteristics of study participants (*e.g.*, demographic, clinical, social) and information on exposures and potential confounders<br>(b) Indicate the number of participants with missing data for each variable of interest<br>(c) *Cohort study* - summarise follow-up time (*e.g.*, average and total amount)			Page 8
Outcome data	15	*Cohort study* - Report numbers of outcome events or summary measures over time<br>*Case-control study* - Report numbers in each exposure			Page 8

For peer review only

		category, or summary measures of exposure<br>*Cross-sectional study* - Report numbers of outcome events or summary measures			
Main results	16	(a) Give unadjusted estimates and, if applicable, confounder-adjusted estimates and their precision (e.g., 95% confidence interval). Make clear which confounders were adjusted for and why they were included<br>(b) Report category boundaries when continuous variables were categorized<br>(c) If relevant, consider translating estimates of relative risk into absolute risk for a meaningful time period			Pages 8-9
Other analyses	17	Report other analyses done—e.g., analyses of subgroups and interactions, and sensitivity analyses			Page 9
**Discussion**					
Key results	18	Summarise key results with reference to study objectives			Page 9
Limitations	19	Discuss limitations of the study, taking into account sources of potential bias or imprecision. Discuss both direction and magnitude of any potential bias		RECORD 19.1: Discuss the implications of using data that were not created or collected to answer the specific research question(s). Include discussion of misclassification bias, unmeasured confounding, missing data, and changing eligibility over time, as they pertain to the study being reported.	Page 11-12
Interpretation	20	Give a cautious overall interpretation of results considering objectives,			Pages 9-11

		limitations, multiplicity of analyses, results from similar studies, and other relevant evidence			
Generalisability	21	Discuss the generalisability (external validity) of the study results			Page 12
**Other Information**					
Funding	22	Give the source of funding and the role of the funders for the present study and, if applicable, for the original study on which the present article is based			Page 13
Accessibility of protocol, raw data, and programming code		..		RECORD 22.1: Authors should provide information on how to access any supplemental information such as the study protocol, raw data, or programming code.	Page 14

*Reference: Benchimol EI, Smeeth L, Guttmann A, Harron K, Moher D, Petersen I, Sørensen HT, von Elm E, Langan SM, the RECORD Working Committee. The REporting of studies Conducted using Observational Routinely-collected health Data (RECORD) Statement. *PLoS Medicine* 2015; in press.

*Checklist is protected under Creative Commons Attribution (CC BY) license.

For peer review only
